# Innovative regioselective synthesis of dispiro[fluorene-9,3′-pyrazole-5′,4″-pyrazolidines]: experimental and computational study

**DOI:** 10.1039/d6ra01559j

**Published:** 2026-05-11

**Authors:** Essam M. Hussein, Ziad Moussa, Munirah M. Al-Rooqi, Saeed S. Samman, Abdulrahman A. Alsimaree, Rabab S. Jassas, Saleh A. Ahmed

**Affiliations:** a Chemistry Chemistry, Faculty of Science, Assiut University 71516 Assiut Egypt essam.hussein78@aun.edu.eg essam.hussein78@yahoo.com; b Department of Chemistry, College of Science, United Arab Emirates University P. O. Box 15551 Al Ain United Arab Emirates zmoussa@uaeu.ac.ae; c Department of Chemistry, Faculty of Science, Umm Al-Qura University 21955 Makkah Saudi Arabia saahmed@uqu.edu.sa saleh_63@hotmail.com; d Department of Chemistry, Faculty of Science, Taibah University Madina Saudi Arabia; e Department of Chemistry, College of Science and Humanities, Shaqra University Shaqra Saudi Arabia; f Department of Chemistry, Jamoum University College, Umm Al-Qura University 21955 Makkah Saudi Arabia

## Abstract

A simple one-pot protocol is described for the synthesis of dispiro[fluorene-9,3′-pyrazole-5′,4″-pyrazolidines] *via* a [3 + 2] cycloaddition reaction between 9-diazo-9*H*-fluorene (DF) and a series of (*E*/*Z*)-4-arylidene-1-phenylpyrazolidine-3,5-diones (APPs). In all cases, the cycloaddition proceeds with complete regioselectivity, affording a single regioisomeric framework as a pair of diastereomers through an *endo* approach. The structures and regiochemical outcomes of the cycloadducts were established by comprehensive 1D and 2D NMR spectroscopic analyses (^1^H, ^13^C, DEPT-135, COSY, ^1^H-HSQC, HMBC, and ROESY). The regiochemistry and mechanism of the cycloaddition reaction were investigated using density functional theory (DFT) calculations at the B3LYP/cc-pVTZ level of theory, supported by analysis of global and dual local electrophilicity and nucleophilicity descriptors. To rationalize the observed stereoselectivity, the relevant transition-state structures were located and optimized using a QST3-based transition-state search at the same level of theory. Global electron density transfer (GEDT) analysis revealed that the cycloaddition reactions are highly polar, with electron density flowing from 9-diazo-9*H*-fluorene (DF) toward the (*E*/*Z*)-4-arylidene-1-phenylpyrazolidine-3,5-dione (APP) framework. Consistently, molecular electrostatic potential surface (MESP) analysis showed that, in the energetically favored transition states, the reacting partners approach through regions of opposite electrostatic potential, leading to stabilizing electrostatic interactions between the two fragments. The computational results are consistent with the experimental observations and support a polar, synchronous one-step cycloaddition mechanism. The developed protocol affords the desired dispiro compounds in good to excellent yields (59–91%) with complete regioselectivity, providing a single regioisomeric framework as a pair of diastereomers. This work provides valuable insights into diazo-based cycloaddition chemistry and is expected to stimulate further research in the synthesis of structurally complex spiroheterocycles. Compared to previously reported approaches, the present method offers a simple one-pot strategy with high efficiency, complete regioselectivity, and operational simplicity.

## Abstract 2

One-pot synthesis of dispiro[fluorene-9,3′-pyrazole-5′,4″-pyrazolidines] *via* a [3 + 2] cycloaddition reaction between 9-diazo-9*H*-fluorene (DF) and a series of (*E*/*Z*)-4-arylidene-1-phenylpyrazolidine-3,5-diones (APPs).

## Graphical abstract

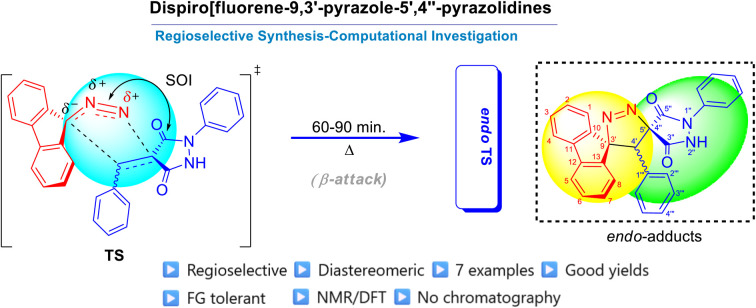

## Introduction

**Fig. 1:**
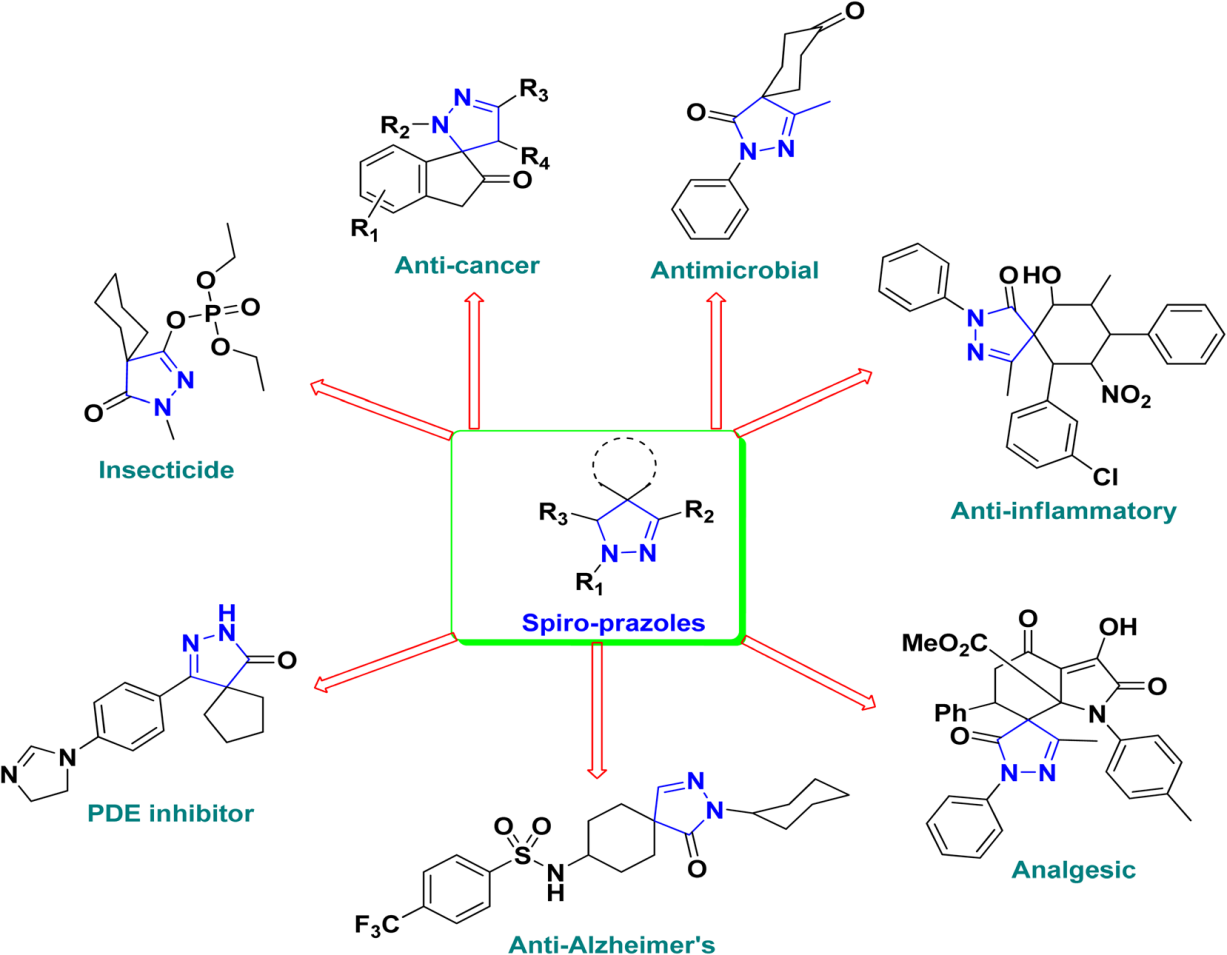
Representative examples of bioactive spiro-pyrazole derivatives reported in the literature.

Over the past decades, the development of efficient and selective synthetic methods for the construction of structurally complex heterocycles has attracted considerable attention in organic and medicinal chemistry.^1^ In this context, spiroheterocycles have emerged as valuable scaffolds in drug discovery owing to their rigid three-dimensional architecture, which can enhance molecular recognition and target selectivity.^2^ Polyfunctionalized pyrazoles represent a privileged class of heterocycles with diverse biological activities, prompting extensive efforts toward their synthesis and functionalization.^3,4^ Spiro-pyrazoles constitute an important subclass of bioactive spiroheterocycles (Fig. 1) and have been reported to exhibit a broad range of pharmacological properties, including antifungal,^5^ antibacterial,^6^ antiviral,^7^ anti-Alzheimer's,^8^ anti-inflammatory and analgesic,^9^ antidepressant,^10^ and antitumor activities.^11^ In addition to their established relevance in medicinal chemistry, dispiro frameworks also hold potential in materials science due to their rigid three-dimensional structures, which can influence electronic properties, molecular recognition, and supramolecular organization.

The [3 + 2] cycloaddition (32CA) reaction between a three-atom component (TAC) and an unsaturated dipolarophile represents a powerful and versatile strategy for the construction of five-membered heterocyclic frameworks with high levels of regio- and stereocontrol.^12^ These multi-component reactions (MCRs) represent an efficient synthetic strategy for the rapid assembly of structurally complex molecules from simple starting materials, often with high atom economy and operational simplicity. In this context, the present one-pot protocol can be viewed as a multi-component transformation that integrates *in situ* generation of the dipolarophile with subsequent cycloaddition. TACs are commonly classified according to their electronic structure and geometry into allylic-type (A-TACs), which typically exhibit a bent configuration (*e.g.*, nitrones), and propargylic-type (P-TACs), which are often described as more linear systems such as nitrile oxides.^12^ Diazo compounds constitute an important and distinct class of 1,3-dipoles that readily participate in 32CA reactions with olefinic and acetylenic dipolarophiles, providing efficient access to pyrazole and pyrazoline derivatives.^13^ Despite this established reactivity, examples involving the cycloaddition of diazo compounds to exocyclic olefinic double bonds for the construction of mono- and dispiro-pyrazole architectures remain relatively scarce.^14^ Although diazo compounds are among the classical 1,3-dipoles employed in [3 + 2] cycloaddition chemistry, their application to exocyclic olefinic dipolarophiles remains comparatively less developed than analogous reactions involving more conventional activated alkenes or alkynes. In exocyclic systems, control of regioselectivity, stereochemical outcome, and substrate-dependent reactivity can be more demanding because the double bond is embedded within a constrained molecular framework, which can limit the approach of the reacting partners and modify both steric and electronic interactions. For this reason, additional well-characterized examples are still valuable for defining the synthetic scope and mechanistic behavior of this class of transformations.

Establishing meaningful relationships between molecular electronic structure and chemical reactivity remains a central objective of theoretical organic chemistry. In recent years, significant progress has been made toward elucidating the mechanisms and selectivity patterns of [3 + 2] cycloaddition (32CA) reactions through combined theoretical and experimental studies. These investigations have contributed to a more detailed understanding of the factors governing regio- and stereochemical outcomes. In this context, Domingo introduced Molecular Electron Density Theory (MEDT) as a complementary conceptual framework, emphasizing the role of electron density reorganization along the reaction pathway in rationalizing chemical reactivity and selectivity.^15^ Nevertheless, despite these advances, the mechanistic features of 32CA reactions involving diazo compounds remain incompletely understood, particularly in structurally constrained systems, where combined experimental and computational investigations are essential for a comprehensive understanding of reactivity and selectivity. Notably, Domingo and co-workers have applied MEDT to the theoretical analysis of the domino 32CA reaction of 1-diazopropan-2-one with 1,1-dinitroethene, providing valuable insights into the polar nature and selectivity of such processes.^16^ While MEDT provides a valuable framework for interpreting polar cycloaddition reactions, its predictive capability may be limited in systems involving multiple competing interactions or weakly polar transition states.

**Scheme 1:**
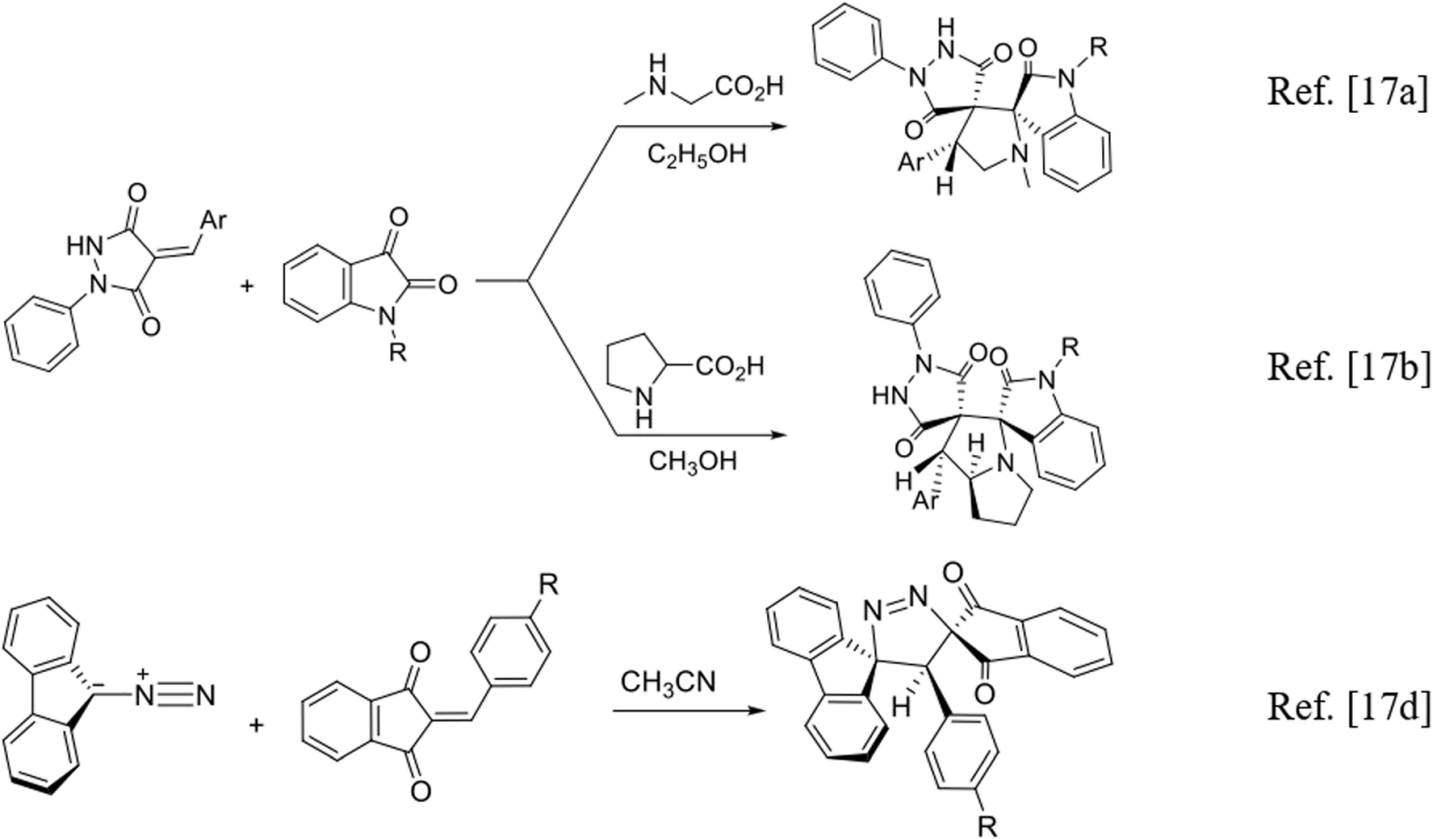
Representative [3 + 2] cycloaddition strategies previously developed by our group for the synthesis of spiro-pyrazole derivatives.

As part of our ongoing investigation of [3 + 2] cycloaddition reactions (Scheme 1),^17^ we report an efficient and straightforward approach to the synthesis of dispiro[fluorene-9,3′-pyrazole-5′,4″-pyrazolidines]. The transformation proceeds through a simple one-pot process involving 9-diazo-9*H*-fluorene (DF) and a series of (*E*/*Z*)-4-arylidene-1-phenylpyrazolidine-3,5-diones (APPs), in which the dipolarophile is formed *in situ* prior to cycloaddition under the reaction conditions (Scheme 3). This [3 + 2] cycloaddition furnishes the target dispiro frameworks in good yields and with complete regioselectivity. The structures of all cycloadducts and the observed regiochemical outcome were established unambiguously by comprehensive 1D and 2D homonuclear and heteronuclear NMR spectroscopic analyses.

To gain deeper insight into the origin of the regio- and stereoselectivity, all feasible cycloaddition pathways were examined computationally through analyses of global and local electrophilicity and nucleophilicity descriptors, together with transition-state calculations, using density functional theory (DFT) at the B3LYP/cc-pVTZ level. Overall, the present study establishes a simple one-pot route to dispiro[fluorene-9,3′-pyrazole-5′,4′′-pyrazolidines] in good yields and with complete regioselectivity. Compared to the limited reported examples of diazo-mediated cycloadditions to exocyclic olefins, the present protocol offers several advantages, including a one-pot procedure, relatively mild conditions, consistently good to excellent yields, and complete regioselectivity. The structural assignment is supported by comprehensive 1D/2D NMR analysis, while the computational results explain the origin of the observed regio- and stereochemical outcome. We anticipate that these findings will be useful both for synthetic access to new spiropyrazole architectures and for the broader mechanistic understanding of diazo-based cycloadditions to exocyclic olefins.

## Results and discussion

### Synthetic and spectroscopic approaches

**Scheme 2:**
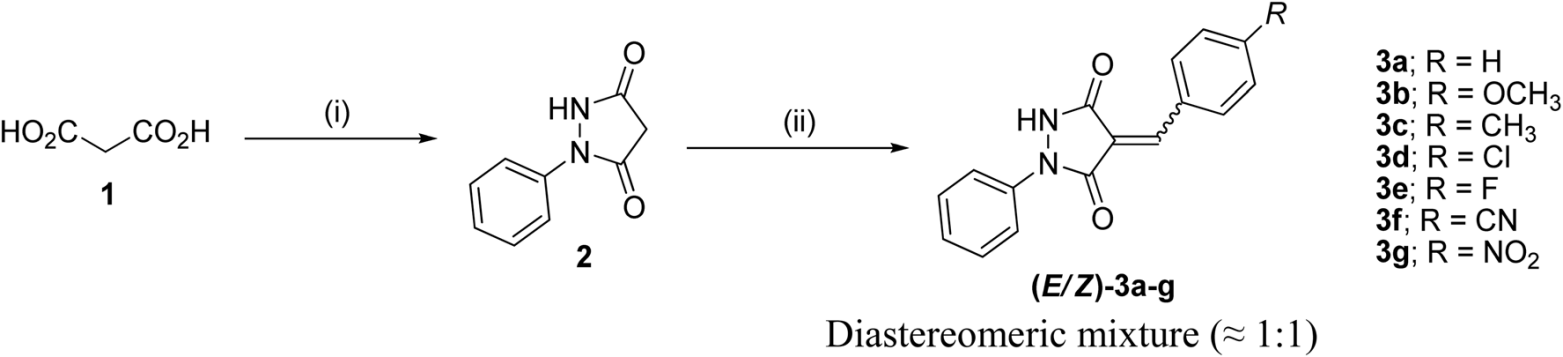
Condensation of (*E*/*Z*)-4-arylidene-1-phenylpyrazolidine-3,5-dione derivatives (APPs) 3a–g. Reagents and conditions: (i) phenylhydrazine, POCl_3_, CHCl_3_, reflux 2 h, (ii) aromatic aldehydes, 1,4-dioxane, reflux 30 min.

1-Phenylpyrazolidine-3,5-dione (2) was prepared from malonic acid (1) and phenylhydrazine using POCl_3_ in chloroform according to a reported procedure.^17*a*^ Condensation of compound 2 with a series of aromatic aldehydes in 1,4-dioxane afforded the corresponding 4-arylidene-1-phenylpyrazolidine-3,5-dione derivatives (APPs) 3a–g as (*E*/*Z*) isomeric mixtures in approximately 1 : 1 ratios, as determined by ^1^H NMR analysis. The individual diastereomers were inseparable owing to their very similar *R*_f_ values and were therefore used directly in the subsequent [3 + 2] cycloaddition reactions without further purification (Scheme 2).

**Scheme 3:**
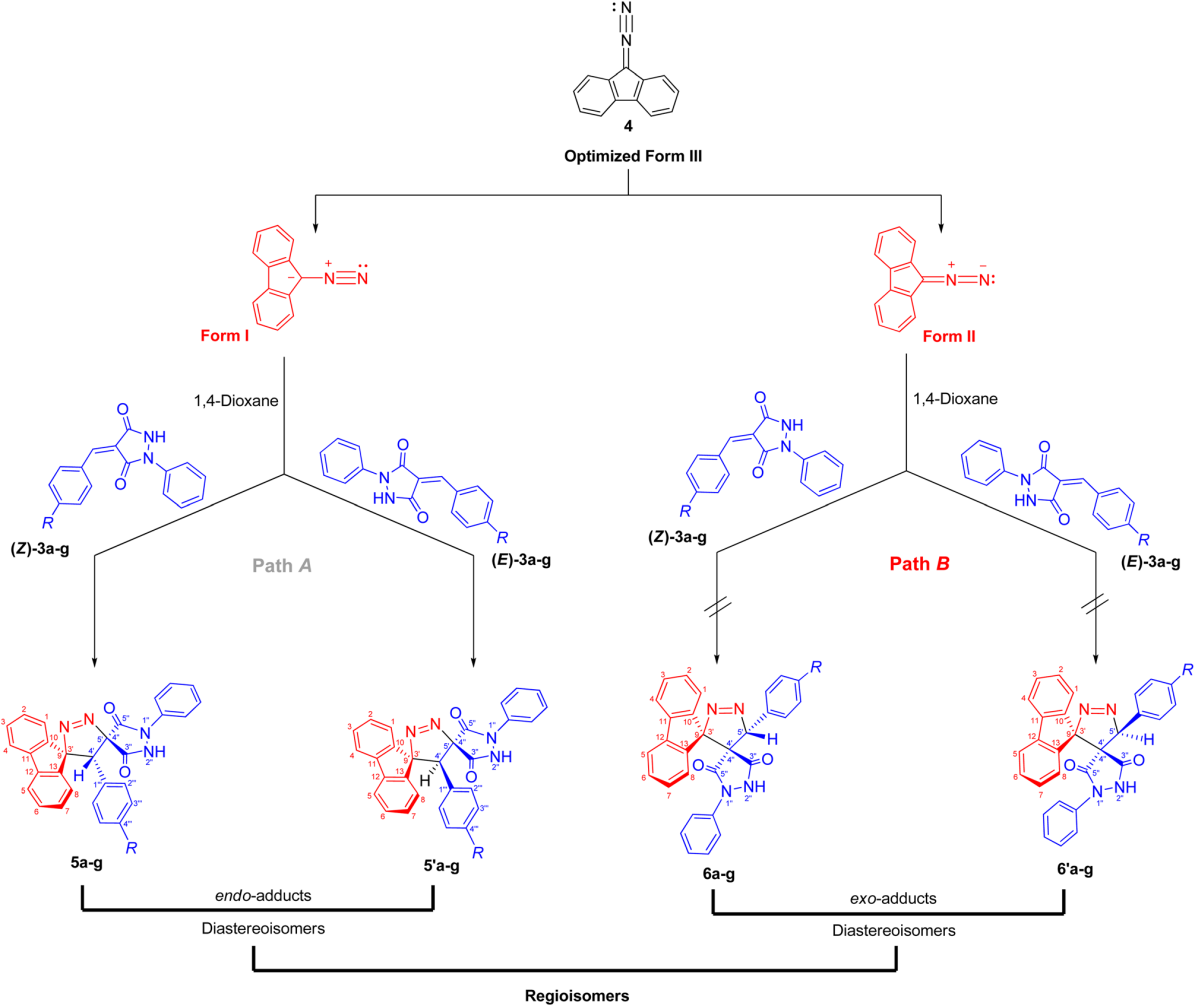
Regioselective synthesis of diastereomeric mixture of 4′-(aryl)-1″-phenyl-4′*H*-dispiro[fluorene-9,3′-pyrazole-5′,4″-pyrazolidine]-3″,5″-diones 5 and 5′.

To evaluate the effect of solvent polarity on the [3 + 2] cycloaddition, the reaction between a diastereomeric mixture of (*E*/*Z*)-4-benzylidene-1-phenylpyrazolidine-3,5-dione (3a) and 9-diazo-9*H*-fluorene (DF, 4) was selected as a representative model system (Scheme 3). A range of solvents of differing polarity and protic character, including methanol, ethanol, acetonitrile, chloroform, tetrahydrofuran (THF), and 1,4-dioxane, were evaluated under reflux conditions (Table 1).

**Table 1:** Effect of solvent polarities on the yield and reaction time of the model [3 + 2] cycloaddition reaction^a^

Entry	Solvent	Time (min)	Yield^b^ (%)	Entry	Solvent	Time (min)	Yield^b^ (%)
1	MeOH	180	42	4	CHCl_3_	180	40
2	EtOH	180	48	5	THF	60	70
3	MeCN	120	66	6	**1,4-Dioxane**	**60**	**78**

a Reaction conditions: DF (4, 1.0 mmol), (*E*/*Z*)-4-benzylidene-1-phenylpyrazolidine-3,5-dione (3a, 1.0 mmol), solvent 10 mL, reflux.

b Isolated yield.

The solvent was found to exert a pronounced influence on both the reaction rate and isolated yield of the cycloaddition. When the model reaction was conducted in aprotic ether solvents, tetrahydrofuran (THF) and 1,4-dioxane afforded the desired cycloadduct in 70% and 78% isolated yields, respectively, after 60 min of reflux (Table 1, entries 5 and 6), with 1,4-dioxane providing the optimal outcome. In acetonitrile, the reaction proceeded more slowly, requiring 120 min to reach completion and furnishing the product in a moderate 66% yield (Table 1, entry 3). In contrast, reactions performed in protic solvents such as methanol and ethanol resulted in substantially lower yields of 42% and 48%, respectively, even after extended heating for 180 min (Table 1, entries 1 and 2). Similarly, chloroform proved ineffective as a reaction medium, affording the cycloadduct in only 40% yield after 180 min of reflux (Table 1, entry 4). These results indicate that aprotic ether solvents favor both the efficiency and rate of the cycloaddition, whereas protic and halogenated solvents are detrimental to cycloadduct formation under the examined conditions. A plausible explanation for the inferior performance of protic solvents is that they may facilitate competitive decomposition or deactivation of the diazo component under reflux conditions, thereby reducing the effective concentration of the reactive 1,3-dipole available for cycloaddition. In addition, hydrogen-bonding interactions in protic media may alter the reactivity of the dipolarophile or disfavor the optimal approach geometry required for productive cycloaddition. By contrast, aprotic ether solvents such as THF and 1,4-dioxane provide a less interfering reaction environment, allowing more efficient preservation of the diazo species and more favorable formation of the cycloadduct. Thus, lower yields are attributed to reduced reaction efficiency under certain solvent conditions rather than recovery of starting materials. Notably, the cycloaddition of 9-diazo-9*H*-fluorene (DF) with diastereomeric mixtures of (*E*/*Z*)-4-arylidene-1-phenylpyrazolidine-3,5-diones (APPs) (≈1 : 1 ratio) proceeded with complete regioselectivity, affording exclusively one regioisomeric framework (5/5′, path A) as a pair of diastereomers, while the alternative regioisomeric products (6/6′, path B) were not detected under the examined conditions (Scheme 3 and Table 2).

**Table 2:** Diastereomeric ratios (*E*/*Z*) of the alkenes 3a–g and the corresponding [3 + 2] cycloadducts as measured by ^1^H-NMR integration^a^

Entry	APP (*E*/*Z*)	R	(*E* : *Z*) Diastereomeric ratios (%)	CA products	(5 : 5′) Diastereomeric ratios (%)	Yield (%)
1	3a	–H	b51 : 49	5a + 5′a	i52 : 48	78
2	3b	–OCH_3_	c51 : 49	5b + 5′b	j50 : 50	65
3	3c	–CH_3_	d49 : 51	5c + 5′c	k61 : 39	79
4	3d	–Cl	e50 : 50	5d + 5′d	l68 : 32	82
5	3e	–F	f52 : 48	5e + 5′e	m55 : 45	59
6	3f	–CN	g49 : 51	5f + 5′f	n51 : 49	91
7	3g	−NO_2_	h50 : 50	5g + 5′g	o51 : 49	89

a Ratio based on the integration of ^1^H-NMR signals at.

b *δ* 8.57 & 8.51.

c *δ* 8.67 & 8.63.

d *δ* 8.51 & 8.46.

e *δ* 8.62 & 8.56.

f *δ* 8.74–8.70 & 8.67.

g *δ* 8.63 & 8.57.

h *δ* 8.68 & 8.61.

i *δ* 8.13 & 8.11.

j *δ* 6.91–6.86 & 6.82–6.75.

k *δ* 8.11 & 8.09.

l *δ* 8.11 & 8.09 and 7.17 & 7.10.

m *δ* 8.11 & 8.09.

n *δ* 8.12 & 8.10.

o *δ* 8.21 & 8.20.

Although the reactions were conducted on a 1 mmol scale, no significant changes in reactivity or selectivity are expected upon scale-up, given the operational simplicity of the protocol and the absence of highly sensitive intermediates. Gram-scale validation will be explored in future studies.

The diastereomeric ratios of both the starting alkenes (3a–g) and the corresponding cycloadducts (5/5′a–g) were determined by integration of diagnostic signals in the ^1^H NMR spectra (Table 2). While the (*E*/*Z*) ratios of the APPs were consistently close to 1 : 1, the isolated cycloadducts displayed modest deviations from an ideal 1 : 1 diastereomeric ratio in some cases (Table 2, entries 3–5). These variations are attributed to differential solubility of the diastereomers during recrystallization, whereby one diastereomer preferentially crystallized while the other remained partially in solution. Indeed, analysis of the crude reaction mixtures by ^1^H NMR prior to purification revealed diastereomeric ratios close to 1 : 1 in all cases, indicating that the observed deviations arise from isolation effects rather than intrinsic diastereoselectivity of the cycloaddition process. Although the present study demonstrates the generality of the cycloaddition across a representative series of arylidene substrates bearing H, OCH_3_, CH_3_, Cl, F, CN, and NO_2_ substituents, it does not yet encompass the full range of substrate compatibility. In particular, strongly electron-donating and strongly electron-withdrawing substituents, as well as sterically demanding aryl groups, may alter the electrophilicity of the exocyclic double bond and affect the *E*/*Z* distribution of the precursor alkene. For example, strongly electron-donating substituents such as NMe_2_ may reduce the electrophilic character of the dipolarophile, while sterically demanding groups may hinder optimal orbital overlap, potentially influencing both reactivity and diastereoselectivity. Such changes could influence the diastereomeric outcome of the cycloaddition, and in cases where one geometric isomer is favored, will lead to preferential formation of a single diastereomer. Accordingly, a broader evaluation of substrate scope and functional-group tolerance will be an important objective of future work.

To fully confirm the chemical structures of the cycloadducts, including the regiochemical outcome and the relative positions of the nitrogen atoms, comprehensive 1D (^1^H, ^13^C, ^13^C-DEPT-135 NMR) and 2D homonuclear and heteronuclear NMR experiments (^1^H–^1^H-DQF-COSY, ^1^H–^13^C-HSQC, ^1^H–^13^C-HMBC, ^1^H–^1^H-ROESY) were performed in DMSO-d_6_ for all compounds (see SI). Using (4′*R*,5′*R*)-4′-(4-nitrophenyl)-1″-phenyl-4′*H*-dispiro[fluorene-9,3′-pyrazole-5′,4″-pyrazolidine]-3″,5″-dione (5g) and (4′*S*,5′*R*)-4′-(4-nitrophenyl)-1″-phenyl-4′*H*-dispiro[fluorene-9,3′-pyrazole-5′,4″-pyrazolidine]-3″,5″-dione (5′g) as a representative example, the regiochemistry and stereochemistry were assigned based on extensive NMR analysis.

**Fig. 2:**
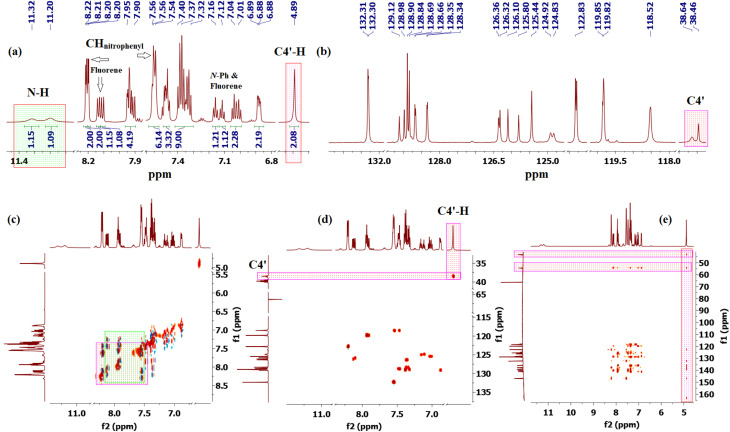
1D and 2D NMR spectra of 4′-(4-nitrophenyl)-1″-phenyl-4′*H*-dispiro[fluorene-9,3′-pyrazole-5′,4″-pyrazolidine]-3″,5″-dione (5g/5′g). (a) Truncated ^1^H-NMR spectrum (b) Truncated ^13^C CRAPT-NMR spectrum; (c) ^1^H–^1^H-COSY-NMR spectrum; (d) ^1^H–^13^C-HSQC-NMR spectrum; (e) ^1^H–^13^C-HMBC-NMR spectrum.

The most diagnostic signal for distinguishing the regioisomers was the methine group (C4′–H), where the proton resonates at *δ* 4.89 ppm (Fig. 2a) and correlates with the C4′ carbon at 38.64/38.46 ppm as confirmed by a cross peak in the ^1^H–^13^C-HSQC spectrum (Fig. 2d).

**Fig. 3:**
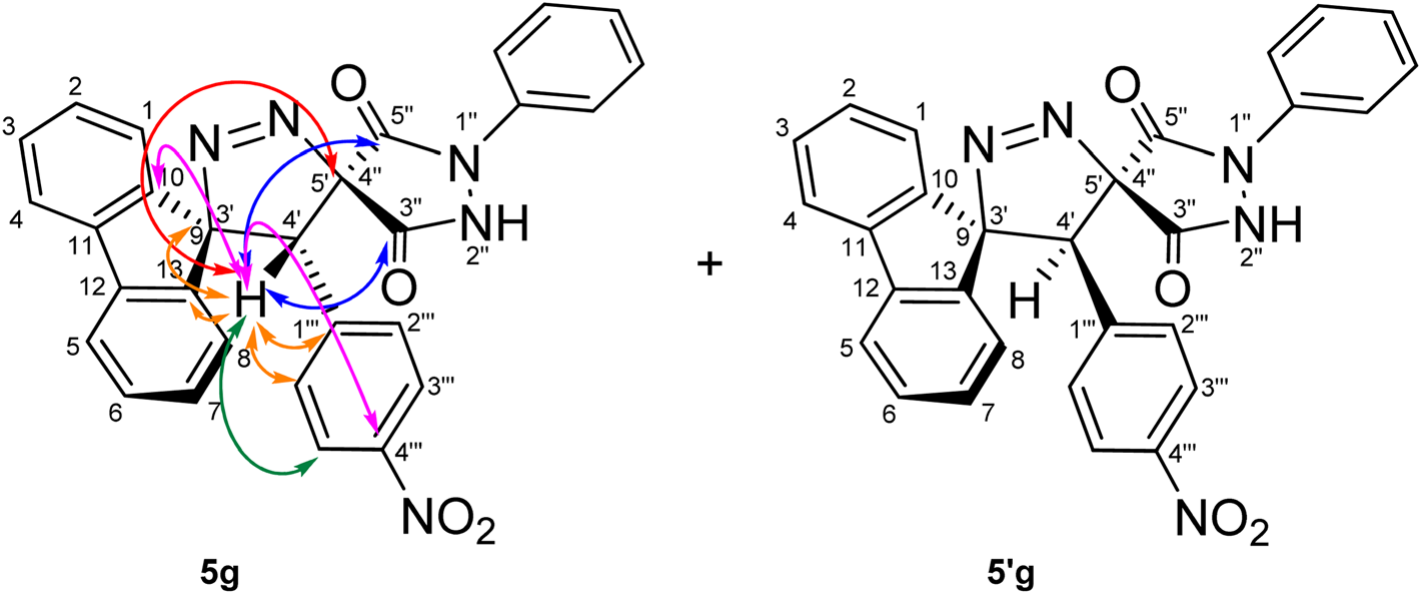
Overview of key correlations observed in the ^1^H–^13^C-HMBC-NMR spectrum of (4′*R*,5′*R*)-4′-(4′-(4-nitrophenyl))-1″-phenyl-4′*H*-dispiro[fluorene-9,3′-pyrazole-5′,4″-pyrazolidine]-3″,5″-dione (5g) and its diastereomer (5′g), highlighting significant long-range correlations between the fluorene, pyrazole, pyrazolidinedione, and 4-nitrophenyl scaffolds and the C4′–H signal.

The unusually downfield chemical shift of C4′–H may be attributed to cumulative deshielding effects arising from the proximity of aromatic rings, adjacent carbonyl groups, and the N–N unit within the pyrazole ring, which collectively place this proton within the deshielding regions of the associated anisotropic magnetic fields. The chemical shifts of the spirocenters, which are farther downfield (C5′/C4″ and C9/C3′, 54.56/54.38, and 43.45/43.19 ppm, respectively), were also instrumental in providing key evidence in support of the regiochemical assignment. In the alternative regioisomer 6g/6′g, a reversal in the relative ordering of the C4′ and the C5′/C4″ spirocenter chemical shifts would be expected, since the C4′ carbon would be directly bonded to a nitrogen atom and is therefore anticipated to resonate further downfield, typically in the 45–50 ppm region. Long-range ^1^H–^13^C-HMBC experiments (Fig. 2e and 3) further supported structure 5, revealing multiple long-range correlations between the aromatic fluorene protons (4J) (*δ* 8.21, 8.20, 7.40–7.34, 7.04, 7.01, and 6.90–6.86 ppm) and the C4′ carbon. In contrast, for regioisomer 6g/6′g, the relevant long-range HMBC correlations would be expected to involve the C4′/C4″ spirocenter (corresponding to C5′/C4″ in regioisomer 5) rather than the C4′ carbon, since correlations between C4′ and the fluorene aromatic protons would require a five-bond (5J) coupling interaction, which is generally too long to be efficiently observed under standard HMBC conditions, thereby providing a clear spectroscopic distinction between the two regioisomeric frameworks.

Beyond these diagnostic comparisons, additional long-range HMBC correlations provide further confirmation of the assigned regiochemistry. Specifically, C4′–H displays long-range HMBC correlations (2J) (Fig. 2e and f) with the proximal fluorene quaternary carbons C10 and C13 (3J) at *δ* 136.19, 135.97, 139.43, 139.14. In addition, cross peaks are observed between C4′–H (*δ* 4.89) and both spirocenters, C5′/C4″ (*δ* 54.56/54.38) and C9/C3′ (43.45/43.19), as well as with the two carbonyl carbons (3J) at *δ* 168.34, 166.97, 163.30, and 161.63. Further HMBC correlations link C4′–H to the 4-nitrophenyl ring, including signals at *δ* 146.96 (C–NO_2_), 137.84, 137.77 (ipso carbons), 132.31(*meta* CH), and 122.83/122.80 (*ortho* CH). Collectively, these long-range correlations establish direct connectivity between the fluorene, pyrazole, pyrazolidinedione, and 4-nitrophenyl scaffolds through the C4′ position, thereby confirming the regiochemical assignment and successful cyclization.

For a concerted cycloaddition process, the reaction is expected to generate a racemic pair of diastereomers depending on the geometric configuration of the dipolarophile employed. Accordingly, since a diastereomeric mixture of (*E*/*Z*)-4-(4-substituted-benzylidene)-1-phenylpyrazolidine-3,5-diones (≈1 : 1 ratio) was used in the [3 + 2] cycloaddition, the formation of an approximately 1 : 1 mixture of diastereomers was observed for all substrates, as anticipated (Fig. 3). The presence of both diastereomers is clearly supported by the NMR data. Distinct diastereomeric signals are evident in the^13^C-DEPT-135 NMR (Fig. 2b), the ^13^C spectrum (SI), and the ^1^H NMR spectrum (Fig. 2a), where many diastereomeric signals are fully resolved. The ^13^C NMR spectrum displays 46 distinct signals, slightly fewer than the 50 unique signals expected for fully resolved diastereomers, which is attributed to partial overlap of resonance frequencies. These include 23 aromatic CH carbons, 2 aliphatic CH carbons, 13 aromatic quaternary carbons, 4 carbonyl carbons, and 4 quaternary sp^3^ carbons. In the ^13^C-DEPT-135 spectrum (Fig. 2b), most diastereomeric C–H signals are well resolved, although some pairs differ by as little as 0.2–3 ppm. Particularly diagnostic diastereomeric signals include those of the carbonyl carbons (*δ* 168.34, 166.97, 163.30, and 161.63), the spirocenters C5′/C4″ (*δ* 54.56/54.38) and C9/C3′ (*δ* 43.35/43.19), and the methine carbon C4′ (*δ* 38.64/38.46). The ^1^H NMR spectra likewise reveal several well-resolved diastereomeric signals. For example, the *ortho* protons of the nitrophenyl ring appear as two overlapped doublets at *δ* 8.21 and 8.20 (d, *J* = 9.0 Hz, 2H each), corresponding to the two diastereomers. The diastereomeric *meta* nitrophenyl protons, as correlated by the ^1^H–^1^H-COSY spectrum with the signals at *δ* 8.21/8.20 (Fig. 2c, purple correlation square), are observed as partially separated doublets in the range *δ* 7.60–7.52. Additional diastereomeric pairs with similar splitting patterns and integrations are observed for the fluorene protons at *δ* 8.13 and 8.10 (d, *J* = 7.8 Hz), and the fluorene and *N*-phenyl triplets at *δ* 7.16, 7.12, 7.04 and 7.01 (t, *J* = 7.8 Hz). Furthermore, the NH protons of the two diastereomers appear as distinct broad singlets at *δ* 11.32 and 11.20 in the ^1^H NMR spectrum and show no correlations in the ^1^H–^13^C-HSQC spectrum (Fig. 2d), consistent with their attachment to nitrogen atoms. An X-ray crystal structure of a representative cycloadduct would, in principle, provide an additional independent structural proof. However, even in the absence of crystallographic data, the present assignments are strongly supported by the full set of 1D and 2D NMR experiments. The products are obtained as two diastereomers, as expected from cycloaddition of an approximately equimolar (*E*/*Z*) alkene mixture, and each diastereomer is formed as a racemic pair under the achiral reaction conditions. The observed paired ^1^H and ^13^C resonances, together with the diagnostic HSQC, HMBC, COSY, and ROESY correlations, provide consistent evidence for both the dispiro framework and the assigned regiochemistry.

### Reaction mechanism

**Scheme 4:**
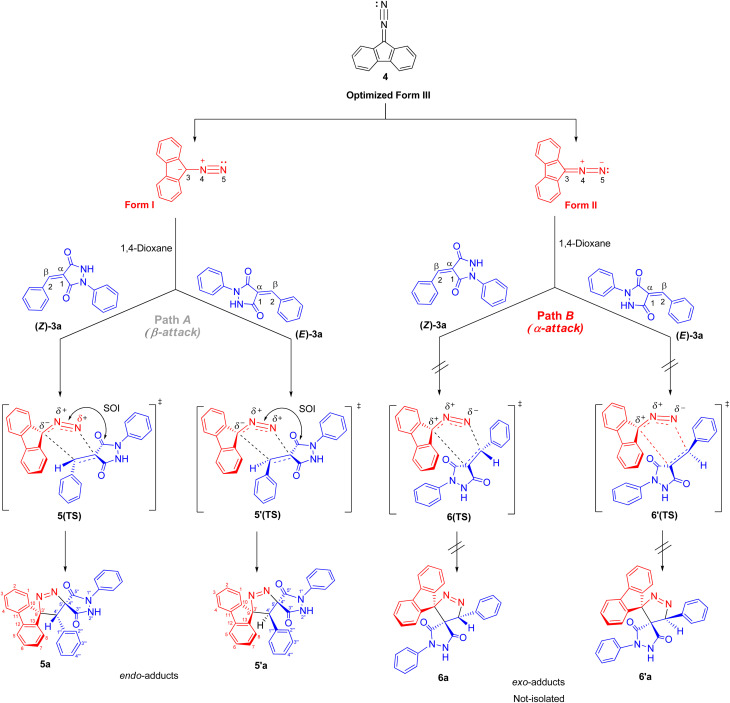
Regio- and stereoisomeric pathways for the 32CA reaction between DF (4) and (*E*/*Z*)-4-benzylidene-1-phenylpyrazolidine-3,5-dione (3a).

The regio- and stereochemistry for the formation of dispiro[fluorene-9,3′-pyrazole-5′,4″-pyrazolidines] 5/5′ rather than the corresponding regioisomers dispiro[fluorene-9,3′-pyrazole-4′,4″-pyrazolidines] 6/6′ can be rationalized based on electronic and steric factors, as proposed in Scheme 4. As shown in Scheme 4, the cycloaddition is favorably regioselective, with bond formation occurring between the nucleophilic carbon of DF (4) (form I) and the β-carbon of the α,β-unsaturated moiety of alkene 3a–g (path A). The regioselectivity can be explained based on electronic polarization of the dipolarophile, arising from the strong electron-withdrawing and resonance effects associated with the two carbonyl groups of alkenes 3a–g, which render the β-carbon the most electrophilic site, together with steric effects imposed by the bulky fluorene and pyrazolidine-3,5-dione moieties, which disfavor approach leading to the *exo* transition state (path B).

Furthermore, the regioselectivity may be further rationalized by considering favorable secondary orbital interactions between the carbonyl group of (*E*/*Z*)-4-arylidene-1-phenylpyrazolidine-3,5-dione derivatives (APPs) 3a–g and the diazo moiety of DF (4), which preferentially stabilize the *endo* transition state, leading to the formation of the *endo*-cycloadduct (path A). Consistent with this interpretation, the regioisomeric products 5/5′ formed *via* path A are preferentially obtained, whereas the alternative *exo*-cycloadducts 6/6′ are not observed under the examined conditions (Table 2).

### Computational study

9-Diazo-9*H*-fluorene (DF) (4) is a 1,3-dipolar three-atom component (TAC) in which four electrons are distributed in a linear C–N–N structure. Initially, DF (4) and two possible *E* and *Z* configurations of dipolarophiles, 4-arylidene-1-phenylpyrazolidine-3,5-diones (APPs) 3a–g, were optimized at B3LYP/cc-pVTZ level of theory. Surprisingly, both configurations were found to be close in energy, with the *E*-configurations calculated to be only ≈0.40–0.60 kcal mol^−1^ more stable than the corresponding *Z*-configurations. This small energy difference is consistent with the experimental observation that the APPs are formed as nearly equimolar (*E*/*Z*) mixtures in all cases (Table 3).

**Table 3:** Geometrical optimization and HOMO and LUMO energies for DF (4) and APPs 3a–g at b3lyp/cc-pvtz level of theory

Comp.	Optimized geometry	*E* (RB3LYP) (kcal mol^−1^)	HOMO–LUMO orbital energy (eV)	Energy gap^a^^*,*^^b^ [Δ*E*, eV]	HOMO–LUMO orbital geometry
4	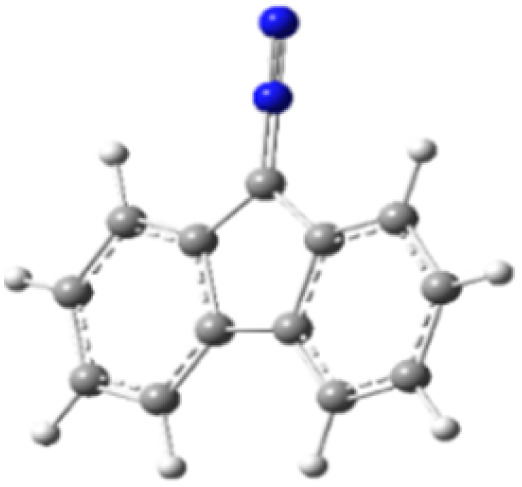	−382702.4	*E*_LUMO_ = −1.973		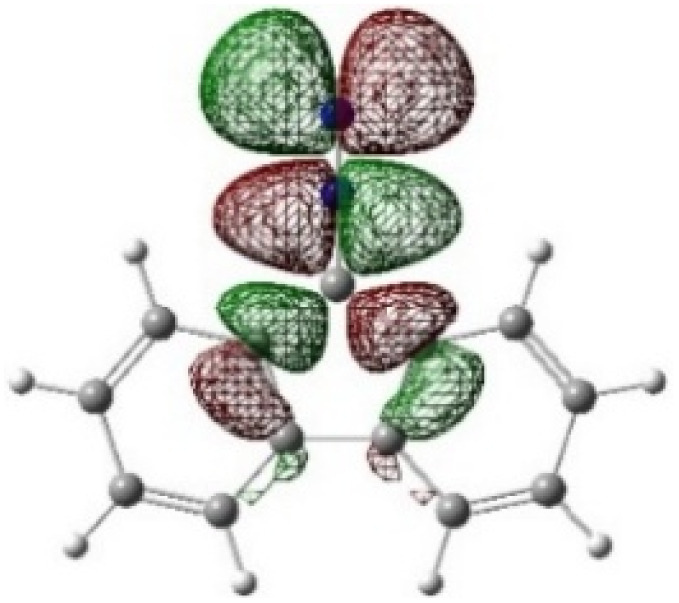
			*E*_HOMO_ = −5.747		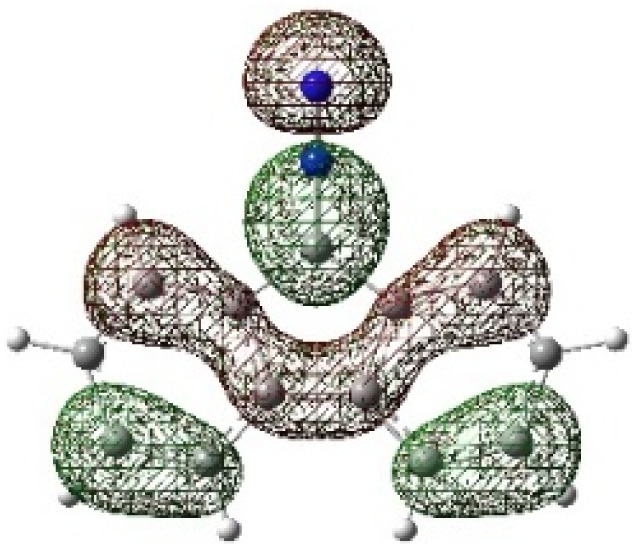
*Z*-3a	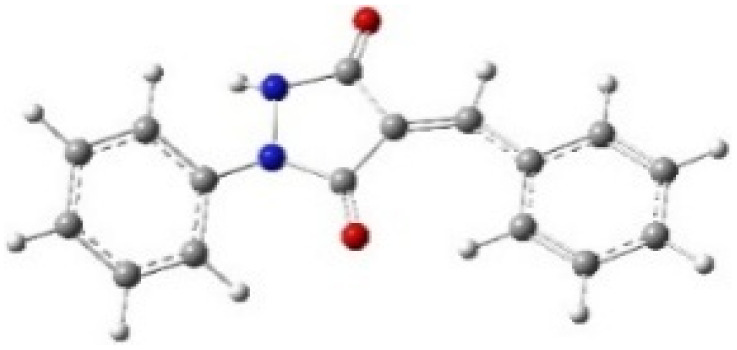	−550433.4	*E*_LUMO_ = −2.841	**2.906** (4.148)	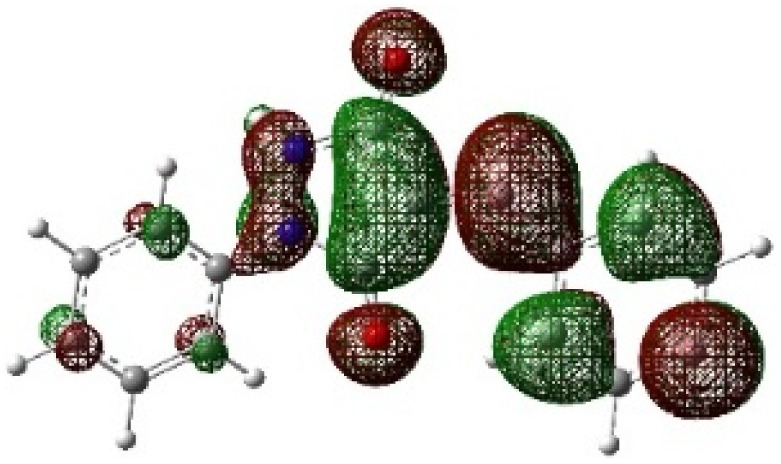
			*E*_HOMO_ = −6.121		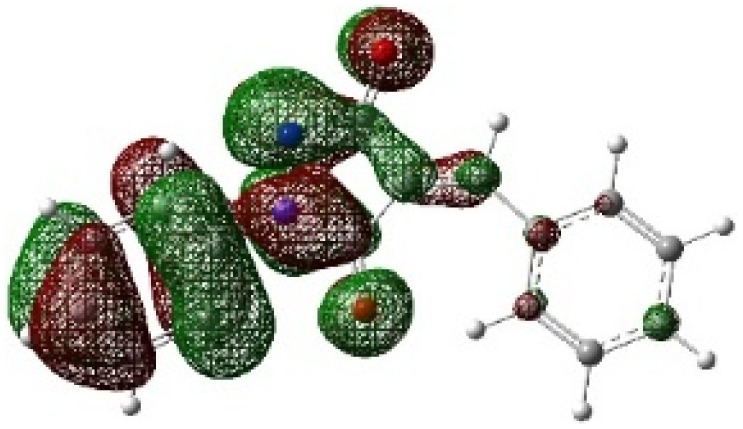
*E*-3a	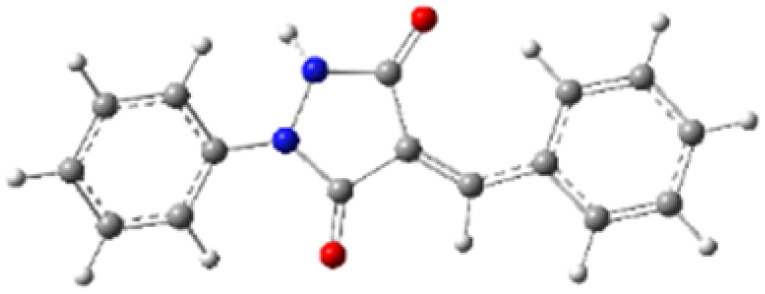	−550433.9	*E*_LUMO_ = −2.859	**2.888** (4.137)	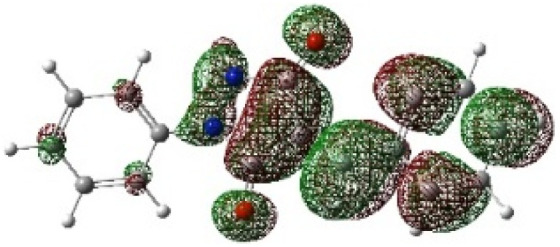
			*E*_HOMO_ = −6.110		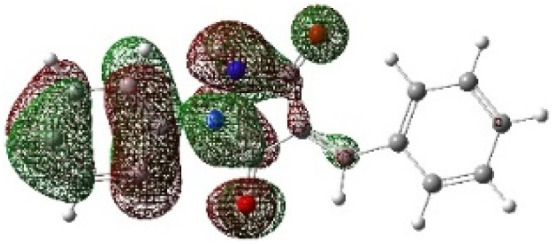
*Z*-3b	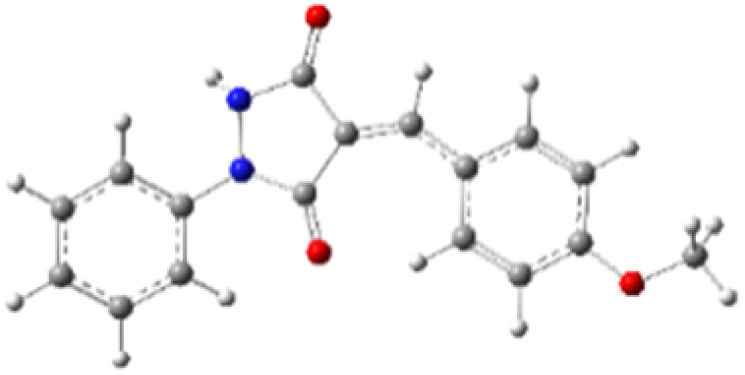	−622327.2	*E*_LUMO_ = −2.600	**3.147** (3.981)	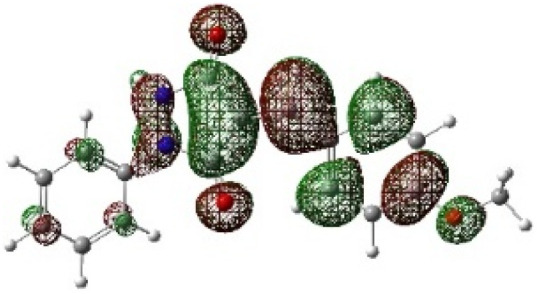
			*E*_HOMO_ = −5.954		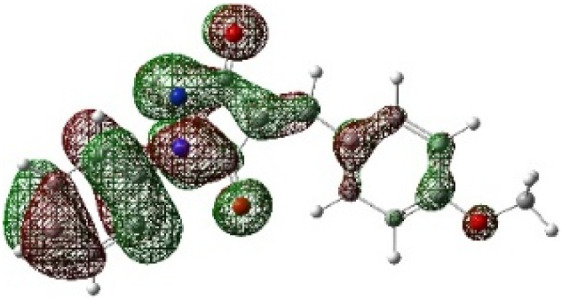
*E*-3b	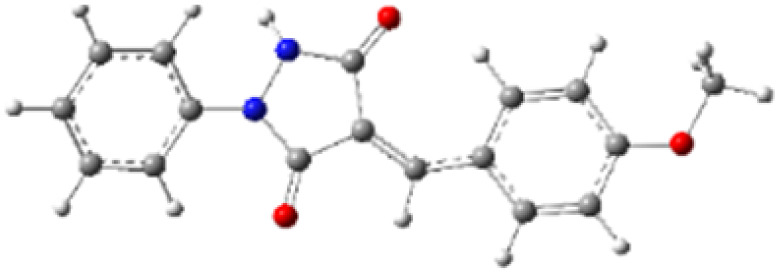	−622327.8	*E*_LUMO_ = −2.628	**3.119** (3.987)	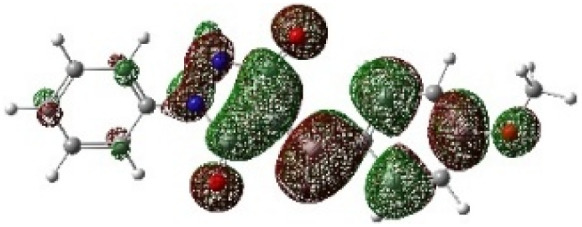
			*E*_HOMO_ = −5.960		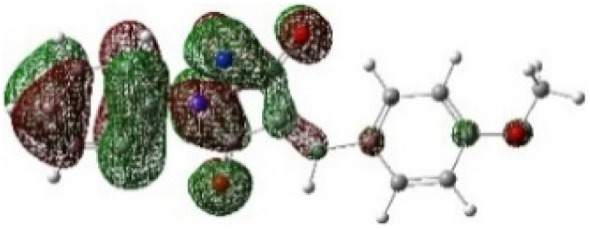
*Z*-3c	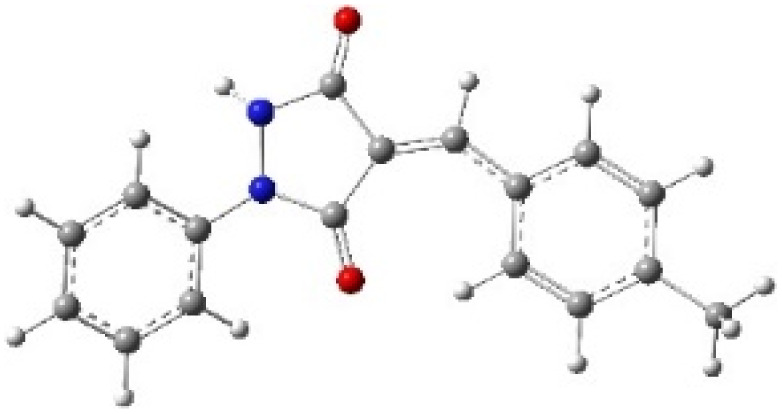	−575115.0	*E*_LUMO_ = −2.745	**3.002** (4.080)	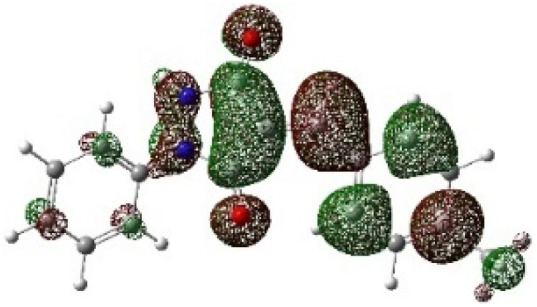
			*E*_HOMO_ = −6.053		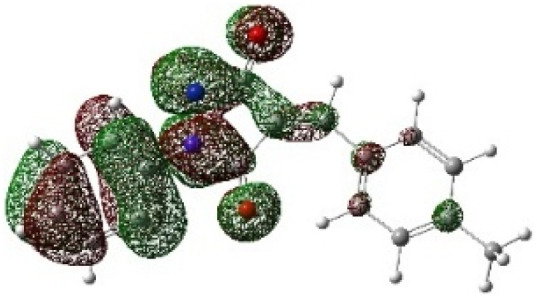
*E*-3c	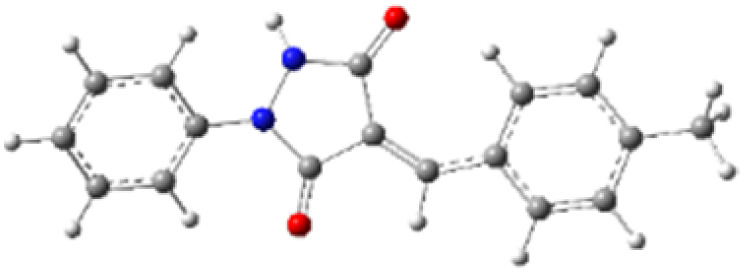	−575115.5	*E*_LUMO_ = −2.761	**2.986** (4.072)	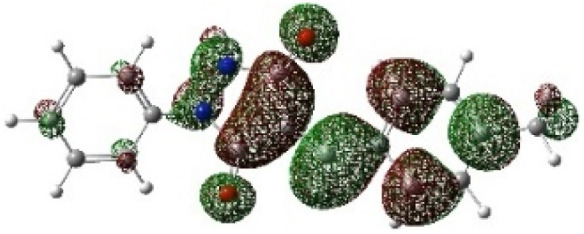
			*E*_HOMO_ = −6.045		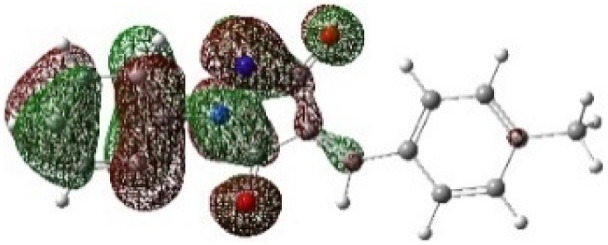
*Z*-3d	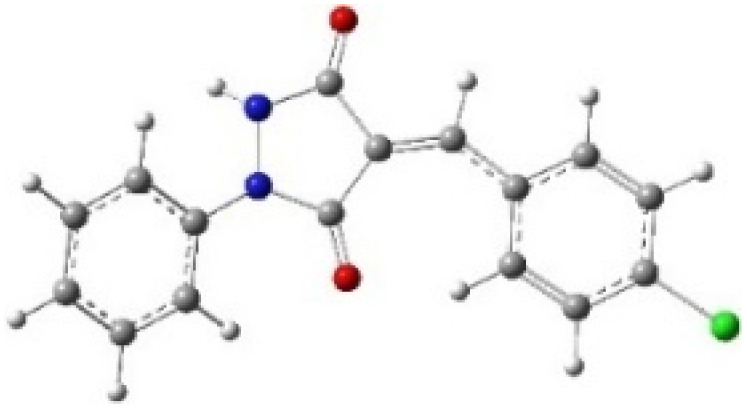	−838856.8	*E*_LUMO_ = −2.986	**2.761** (4.230)	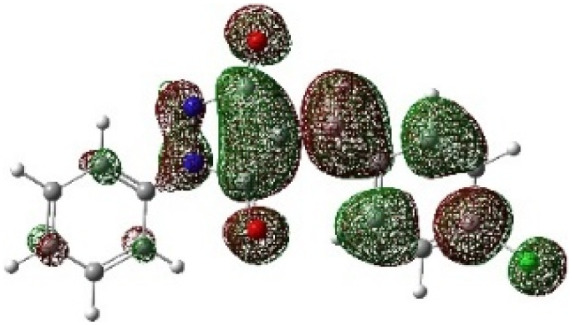
			*E*_HOMO_ = −6.203		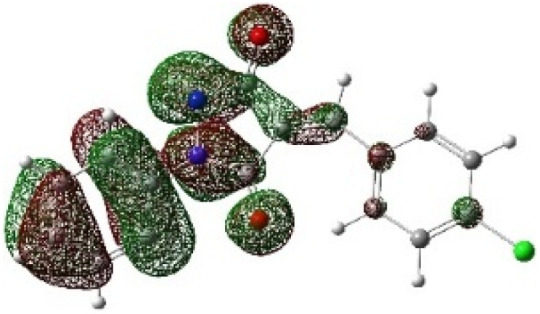
*E*-3d	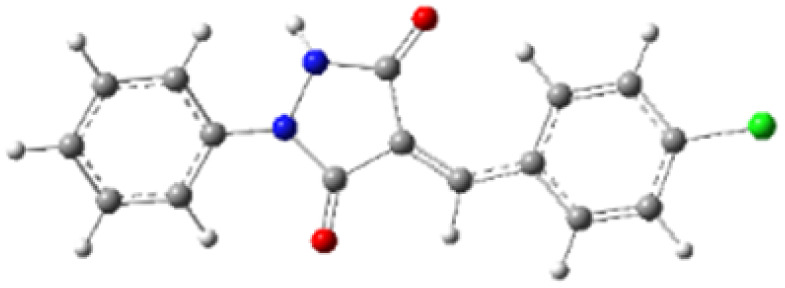	−838857.3	*E*_LUMO_ = −3.004	**2.743** (4.216)	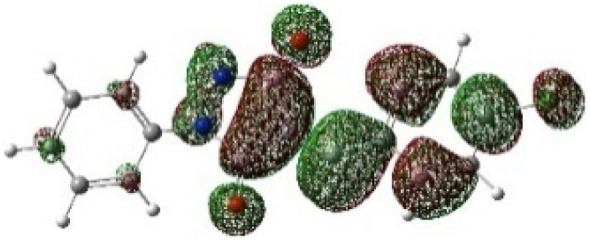
			*E*_HOMO_ = −6.189		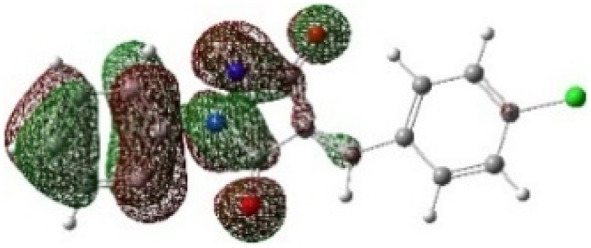
*Z*-3e	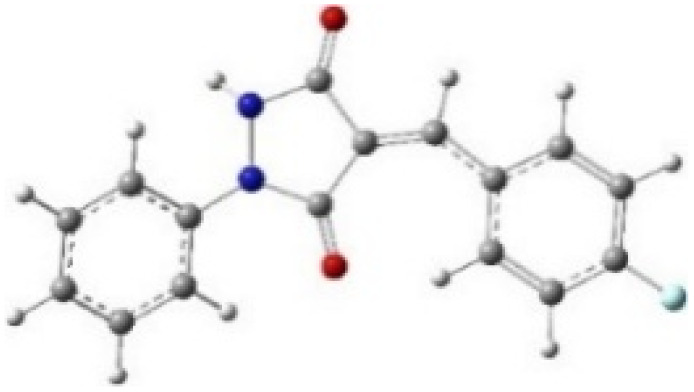	−612730.0	*E*_LUMO_ = −2.883	**2.864** (4.197)	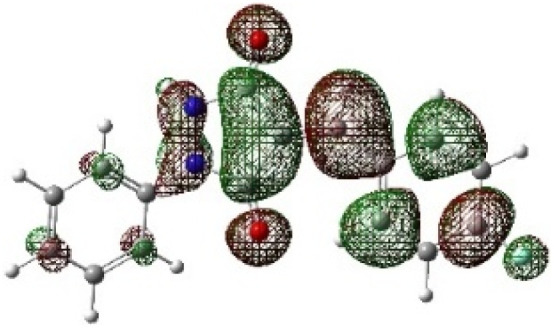
			*E*_HOMO_ = −6.170		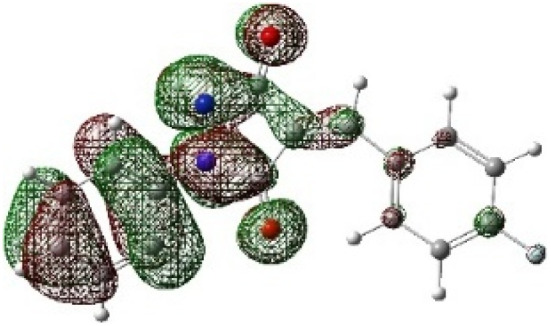
*E*-3e	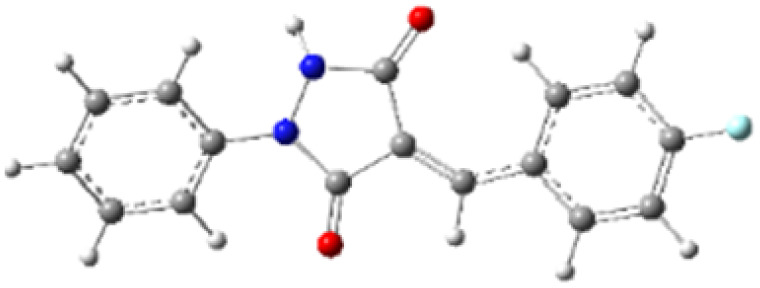	−612730.5	*E*_LUMO_ = −2.902	**2.845** (4.183)	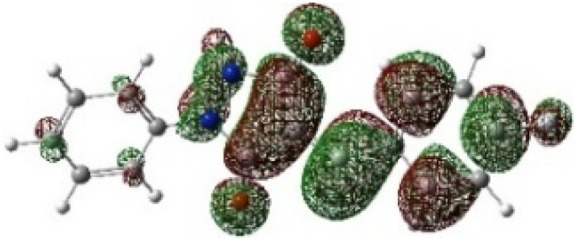
			*E*_HOMO_ = −6.15**6**		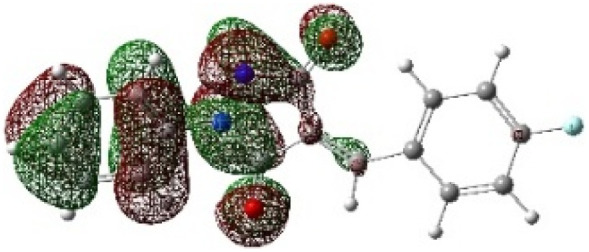
*Z*-3f	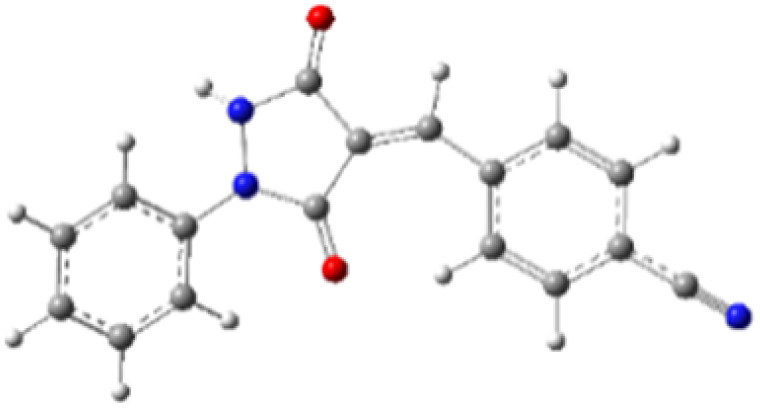	−608334.5	*E*_LUMO_ = −3.40**1**	**2.346** (4.427)	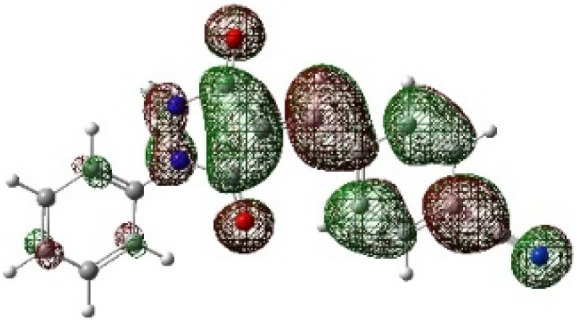
			*E*_HOMO_ = −6.400		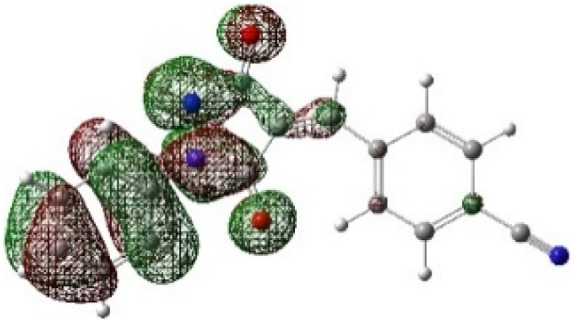
*E*-3f	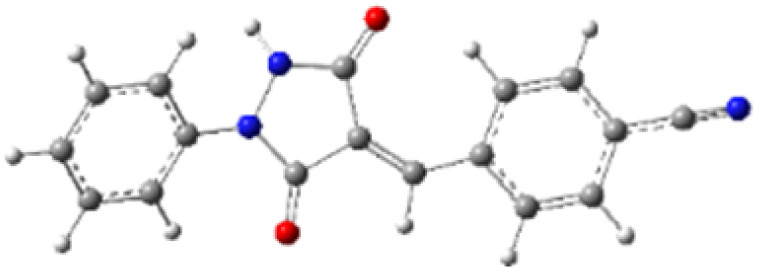	−608334.9	*E*_LUMO_ = −3.419	**2.328** (4.407)	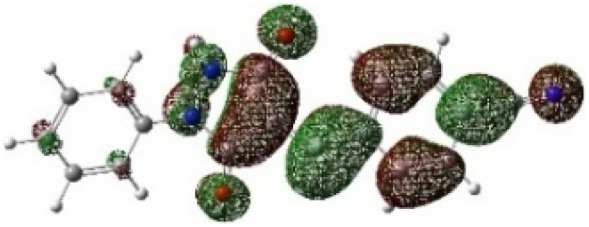
			*E*_HOMO_ = −6.380		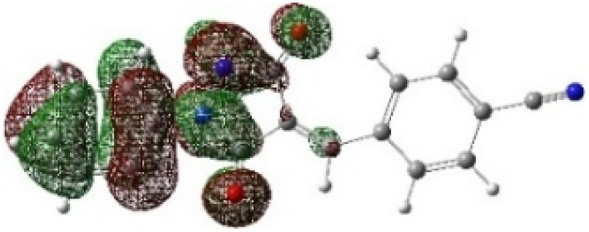
*Z*-3g	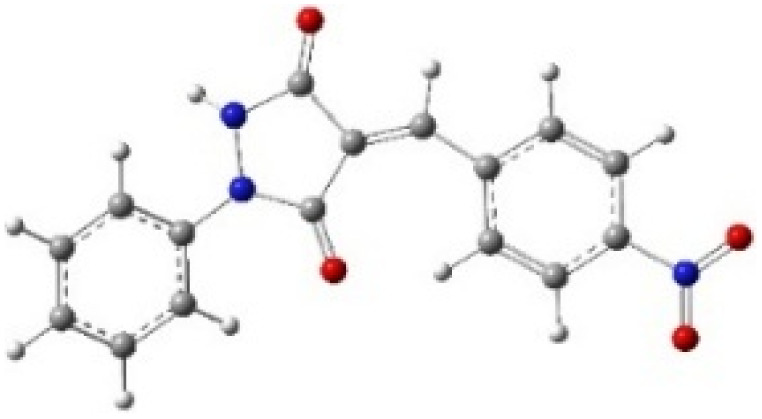	−678806.2	*E*_LUMO_ = −3.597	**2.150** (4.465)	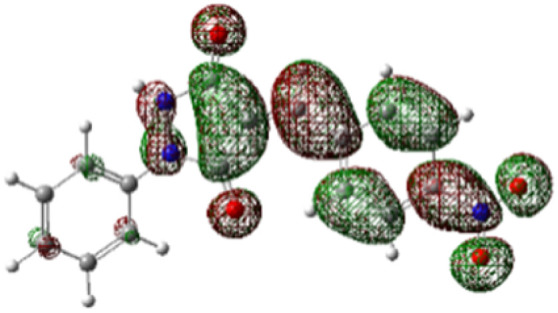
			*E*_HOMO_ = −6.438		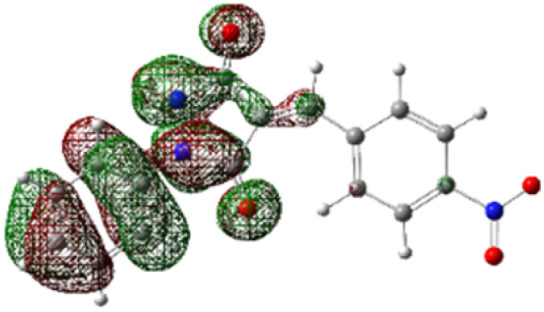
*E*-3g	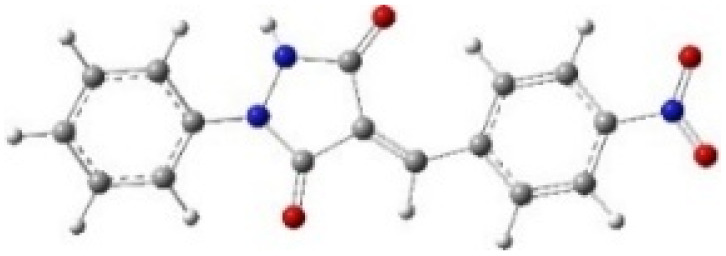	−678806.6	*E*_LUMO_ = −3.613	**2.134** (4.446)	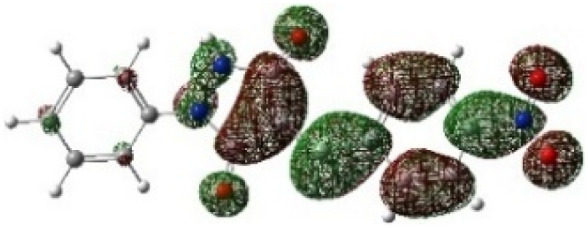
			*E*_HOMO_ = −6.419		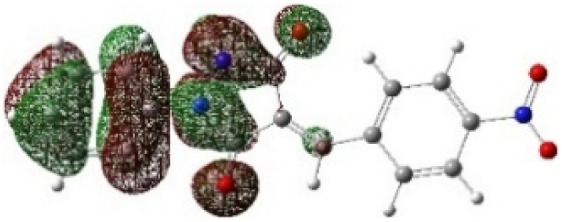

a Δ*E* = LUMO(APPs 3a–g) − HOMO(DF 4) [normal electron demand].

b (Δ*E*) = LUMO(DF 4) − HOMO(APPs 3a–g) [inverse electron demand].

#### Analysis of frontier molecular orbital (FMO) and conceptual density functional theory (CDFT) reactivity indices

The Frontier Molecular Orbital (FMO) approach, originally developed by Fukui,^18^ provides a useful qualitative framework for analyzing reaction mechanisms and reactivity trends. 32CA reactions are commonly rationalized in terms of HOMO–LUMO interactions and can be classified according to the electron-demand type of the cycloaddition based on the relative energies of the interacting frontier orbitals of the dipole and dipolarophile.^19^ The calculated frontier orbital energies for APPs 3a–g and DF (4) are summarized in Table 3. Inspection of the frontier orbital energies listed in Table 3 indicates that the cycloaddition reactions between dipolarophiles 3a–g and dipole 4 proceed under normal electron-demand conditions. In all cases, the HOMO of the dipole DF (4) lies at higher energy than the HOMOs of the corresponding dipolarophiles, while the LUMOs of APPs 3a–g are significantly lower in energy than the LUMO of DF. As a result, the HOMO(dipole)–LUMO(dipolarophile) energy gaps fall in the range of approximately 2.1–3.2 eV and are consistently smaller than the corresponding inverse electron-demand gaps, which lie in the range of approximately 4.0–4.5 eV (Table 3). These relationships identify the interaction between the HOMO of DF and the LUMO of the dipolarophile as the dominant frontier orbital interaction governing the cycloaddition process.

While frontier molecular orbital analysis provides a useful qualitative description of the dominant orbital interactions, a more detailed understanding of reactivity and selectivity in polar cycloaddition processes can be obtained from conceptual density functional theory (CDFT). In recent years, CDFT has emerged as a powerful framework for the investigation of chemical reactivity, allowing quantitative characterization of electrophilic and nucleophilic behavior through global and local reactivity indices derived from ground-state electron density descriptors.^20,21^ In particular, analysis of global and local CDFT indices has proven especially effective for describing polar reaction pathways, including the cycloaddition reactions, where charge transfer plays a significant role in governing reactivity and selectivity.^21–23^ Likewise, electrophilicity and nucleophilicity indices within the CDFT framework have been successfully employed to assess the feasibility of [3 + 2] cycloaddition reactions and to classify dipole–dipolarophile pairs according to their polar character.^24^

#### Analysis of global reactivity indices of the CA reactants at the ground states

The ability of a molecule to transfer charge in its ground state can be described by the electronic chemical potential *µ*, which is defined as the mean of one-electron energies of the frontier molecular orbitals HOMO and LUMO, as *µ* = −(*I* + *A*)/2, where *I* (ionization potential) = −*E*_HOMO_ and *A* (electron affinity) = −*E*_LUMO_.^25^ The global electronegativity (*χ*) is rigorously defined as the negative of the electronic chemical potential (*µ*) as *χ* = −*µ* = (*I* + *A*)/2.^25^ The chemical hardness *η*, which is a measure of the resistance of a system to changes in its electron population, reflects the difficulty of charge transfer and is conceptually related to molecular stability and polarizability. *η* can be defined as *η* = (*I* − *A*). Accordingly, softness, *S*, which represents the propensity of a system to undergo charge redistribution, is defined as the inverse of the hardness, *S* = 1/*η*.^26^ The global electrophilicity index (*ω*), which measures the stabilization in energy when the system acquires an additional electronic charge from the environment, is defined as *ω* = *µ*^2^/2*η*, in terms of the electronic chemical potential *µ* and the chemical hardness *η*.^27^ The global reactivity indices *χ*, *µ*, *η*, *S*, and *ω* were calculated for all reactants and are summarized in Table 4.

**Table 4:** Global properties and global electrophilicity/nucleophilicity indices values for DF (4) and APPs 3a–g involved in the CA reactions^a^

Reactant	*χ* (eV)	*µ* (eV)	*η* (eV)	*S* (a.u.)	*ω* (eV)	*ω* ^+^ (eV)	*ω* ^−^ (eV)	Δ*ω*^±^ (eV)	*N* b (eV)	*N*′ (eV)	*N*″ (eV)
4	3.860	−3.86	3.774	7.218	1.974	0.516	4.376	4.892	3.664	0.507	2.285
*Z*-3a	4.481	−4.481	3.280	8.296	3.061	1.23	5.712	6.942	3.290	0.327	1.751
*E*-3a	4.484	−4.484	3.252	8.372	3.092	1.257	5.741	6.998	3.301	0.323	1.742
*Z*-3b	4.277	−4.277	3.353	8.123	2.728	1.008	5.285	6.293	3.458	0.367	1.892
*E*-3b	4.295	−4.295	3.332	8.171	2.767	1.037	5.331	6.368	3.450	0.361	1.876
*Z*-3c	4.399	−4.399	3.308	8.221	2.925	1.139	5.538	6.677	3.358	0.342	1.806
*E*-3c	4.403	−4.403	3.284	8.296	2.952	1.161	5.563	6.724	3.367	0.339	1.797
*Z*-3d	4.595	−4.595	3.217	8.451	3.281	1.386	5.980	7.366	3.208	0.305	1.672
*E*-3d	4.597	−4.597	3.185	8.530	3.317	1.417	6.014	7.430	3.222	0.301	1.663
*Z*-3e	4.527	−4.527	3.287	8.271	3.116	1.264	5.791	7.055	3.241	0.321	1.727
*E*-3e	4.529	−4.529	3.254	8.372	3.152	1.294	5.823	7.117	3.256	0.317	1.717
*Z*-3f	4.900	−4.900	2.998	9.070	4.005	1.929	6.829	8.759	3.012	0.25	1.464
*E*-3f	4.899	−4.899	2.961	9.193	4.054	1.974	6.874	8.848	3.031	0.247	1.455
*Z*-3g	5.018	−5.018	2.84	9.581	4.432	2.278	7.296	9.574	2.973	0.226	1.371
*E*-3g	5.016	−5.016	2.806	9.683	4.484	2.326	7.342	9.669	2.993	0.223	1.362

a All computations were carried out with the Gaussian 09 suite of programs. Calculations based on the method of DFT were performed at the B3LYP/cc-pVTZ level of the theory.

b HOMO energy of tetracyanoethylene (TCE) is −0.34586 (in a.u.) at the same level of theory.

The electronic chemical potential, *µ*, of the DF (4) (*µ* = −3.860 eV) is higher than those of the APPs 3a–g (−5.018 < *µ* < −4.277 eV). Furthermore, APPs 3a–g act as good electrophiles due to the larger value of their electrophilicity *ω* (2.728 < *ω* < 4.484) relative to the electrophilicity value of DF (4) (*ω* = 1.974 eV); therefore, charge transfer is expected to occur from DF (4), acting as the nucleophilic dipole, to APPs 3a–g, acting as electrophilic dipolarophiles. Recently, a unique electrophilicity scale was developed to classify reagents involved in CA reactions.^28^ Analysis of the electrophilicity indices *ω* presented in Table 4 shows that APPs 3a–g are among the strong electrophiles, while DF (4) is classified as marginal electrophile, consistent with its nucleophilic character, suggesting that in a polar 32CA reaction, DF (4) and APPs 3a–g act as nucleophile and electrophile, respectively. Consequently, in this model, the polar character of a dipole–dipolarophile interaction can be evaluated by the difference Δ*ω* in the global electrophilicity of the two reagents. The 32CA of DF as dipole with APPs as dipolarophile (Δ*ω* values in the range of 0.754–2.509 eV) lie well below the threshold value of 4.5 eV, indicating that these reactions proceed through a polar, one-step mechanism.

To further characterize the charge-accepting and charge-donating tendencies of the reactants, additional reactivity indices have been defined in terms of the electroaccepting, *ω*^+^, and electrodonating, *ω*^−^ powers, as proposed by Gázquez *et al.*^29^ Both quantities are calculated by employing the vertical ionization energy *I* and electron affinity *A* according to *ω*^+^ = *A*^2/(2(*I*−*A*)^, *ω*^−^ = *I*^2/(2(*I*−*A*))^. Here, *ω*^+^ represents the measure of the tendency of a given system to accept charge, whereas *ω*^−^ is the tendency to donate charge. It is important to mention here that a larger *ω*^+^ value of a system reflects a better capability of accepting charge, whereas a smaller value of *ω*^−^ corresponds to a better electron donor (Table 4). To directly relate the electroaccepting and electrodonating powers, the following definition of net electrophilicity (Δ*ω*^±^) has been suggested as Δ*ω*^±^ = *ω*^+^ − (−*ω*^−^) = *ω*^+^ + *ω*^−^, which represents the electron accepting power relative to the electron donating power and it has been defined as the net electroaccepting power. This global reactivity descriptor measures the relative electrophilicity.^30^

It is important to note that according to these definitions and as summarized in Table 4, the APPs (*E*/*Z*)-3a–g exhibit larger values of *ω*^+^ (*ω*^+^ = 1.037/1.008–2.326/2.278, respectively) and therefore possess a greater ability to accept electronic charge, classifying them as strong electrophiles, while DF (4) displays a smaller value of *ω*^−^(*ω*^−^ = 4.376) and thus exhibits a greater tendency to donate electronic charge, consistent with its behavior as a good nucleophile.

In addition to these electrophilicity-based descriptors, a working model for nucleophilicity can be obtained using the empirical nucleophilicity index *N*, which was originally proposed on the basis of HOMO energies and is defined as *N* = *E*_HOMO_ (eV) − *E*_HOMO(TCE)_ (eV).^31^ Nucleophilicity values *N* for the series of APPs 3a–g and DF (4) are listed in Table 4. Notably, the relatively high nucleophilicity index of DF (4) (*N* = 3.664 eV) indicates that it is the most nucleophilic species in the studied series, fully consistent with its role as the electron donor toward APPs 3a–g.

Moreover, based on the assumption of Chattaraj *et al.* that electrophilicity and nucleophilicity are inversely related,^32,33^ the proposed nucleophilicity parameter can be described as the multiplicative inverse of the electrophilicity index (*ω*) and is expressed as *N*′ = 1/*ω*.^28,34^ In this context, Roy *et al.* proposed the nucleophilicity index *N*″ based on the mutual electrodonating *ω*^−^ power.^35^ Because the nucleophilicity index determined as 1/*ω*^−^ is typically less than unity, the nucleophilicity index *N*″ has been defined as *N*″ = ((1/*ω*^−^) × 10). As revealed in Table 4, the calculated *N*′ and *N*″ values follow the same qualitative order as observed for the related nucleophilicity *N* descriptor. Thus, a comparison of the values obtained with the three nucleophilicity models, namely *N*,*N*′, and Roy's *N*″, shows a consistent and rational agreement with the experimental observations.

#### Analysis of local reactivity indices of the CA reactants at the ground states

The regiochemistry of polar cycloaddition reactions can be investigated using local parameters of reactivity. Local quantities such as the Fukui function *f*(*r*), Parr function *P*(*r*), local philicity and local softness *s*(*r*) indices are used to describe the reactivity and selectivity of a specific site in a molecule.

The condensed forms of the Fukui functions (*f*_k_^±^) of an atom, k, in a molecule with *N* electrons, depending on the type of electron transfer, can be calculated using the procedure proposed by Yang and Mortier, based on a finite difference method,^36^ according to:*f*_k_^+^ = *q*_k_ (*N* + 1) − *q*_k_ (*N*) for nucleophilic attack*f*_k_^−^ = *q*_k_ (*N*) − *q*_k_ (*N* − 1) for electrophilic attack.

Recently, Morell *et al.*^37^ proposed a more refined local reactivity descriptor, defined as a dual descriptor (*f*^(2)^_k_ or Δ*f*_k_), which corresponds to the derivative of molecular hardness with respect to the external potential and can be expressed as the difference between the nucleophilic and electrophilic Fukui Functions, given by *f*^(2)^_k_ ≡ Δ*f*_k_ = *f*_k_^+^ − *f*_k_^−^.

The dual descriptor is considered more efficient than the conventional Fukui function for predicting the reactive sites of a molecule. Moreover, it is less affected by orbital contraction or expansion than the Fukui function and seems to be more rational. The use of *f*^(2)^_k_ provides the net nucleophilicity (or net electrophilicity) at a given atomic site in terms of the net amount of electron transfer in each direction.

When *f*^(2)^_k_ < 0, an electrophilic attack on atom k is favored, and the atom behaves as a nucleophilic site; conversely, when *f*^(2)^_k_ > 0, a nucleophilic attack on atom k is favored, and the atom behaves as an electrophilic site.

Furthermore, Chattaraj *et al.*^38^ have introduced the concept of generalized philicity, which incorporates information from previously known different global and local reactivity and selectivity descriptors, in addition to the information regarding electrophilic/nucleophilic power of a given atomic site in a molecule (*ω*_k_^±^). It is possible to define a local quantity called philicity associated with a site k in a molecule by means of the corresponding condensed Fukui functions (*f*_k_^±^), according to *ω*_k_^±^ = *ω f*_k_^±^, where *ω*_k_^+^ and *ω*_k_^−^ represent local philic quantities describing nucleophilic and electrophilic attacks, respectively.

In the light of the dual descriptor^37^ and local philicity,^38^ Padmanabhan *et al.*^39^ proposed a multiphilic descriptor (Δ*ω*_k_) within the unified philicity concept, which enables simultaneous description of both the nucleophilic and electrophilic nature of a chemical species in nucleophile–electrophile interactions. It is defined as the difference between the nucleophilic and electrophilic condensed philicity functions and is given by Δ*ω*_k_ = *ω*_k_^+^ − *ω*_k_^−^ = *ωf*^(2)^_k_. When Δ*ω*_k_ < 0, the site is favored for an electrophilic attack, whereas when Δ*ω*_k_ > 0, the site may be favored for a nucleophilic attack. Local condensed Fukui *f*_k_^±^, Fukui dual descriptor *f*^(2)^_k_, and multiphilic descriptor Δ*ω*_k_ values are listed in Table 5.

**Table 5:** Nucleophilic and electrophilic Fukui *f*_k_^±^, Fukui dual descriptor *f*^(2)^_k_, multiphilic descriptor Δ*ω*_k_, Parr indices *P*_k_^±^, local electrophilicity *ω*_k_, local nucleophilicity *N*_k_ and local reactivity difference index *R*_k_ for the most relevant heavy atoms of DF (4) and APPs 3a–g^a^

Comp.	Site k		*f*_k_^+^	*f*_k_^−^	*f* ^(2)^_k_	*ω*_k_^+^	*ω*_k_^−^	Δ*ω*_k_	*P*_k_^+^	*P*_k_^−^	*ω*_k_	*N*_k_	*R*_k_
4	3	C	0.011	0.180	**−0.169**	0.022	0.355	**−0.334**	−0.056	0.233	−0.111	0.854	−0.97
	4	N	0.210	−0.017	0.193	0.415	−0.034	0.381	0.302	−0.037	0.596	−0.136	+0.73
	5	N	0.300	0.212	0.088	0.592	0.419	0.174	0.672	0.372	1.327	1.363	±1.35
*Z*-3a	1	C	0.063	−0.019	0.044	0.193	−0.058	0.135	0.048	0.048	0.147	0.158	±0.15
	2	C	0.180	0.049	**0.131**	0.551	0.150	**0.401**	0.424	−0.018	1.298	−0.059	+1.36
*E*-3a	1	C	0.062	−0.029	0.033	0.192	−0.090	0.102	0.048	0.031	0.148	0.102	±0.13
	2	C	0.182	0.051	**0.131**	0.563	0.158	**0.405**	0.425	−0.015	1.314	−0.050	+1.36
*Z*-3b	1	C	0.051	0.021	0.029	0.139	0.057	0.079	0.036	0.096	0.098	0.332	−0.23
	2	C	0.184	0.017	**0.167**	0.502	0.046	**0.455**	0.440	−0.047	1.200	−0.163	+1.36
*E*-3b	1	C	0.050	0.013	0.037	0.138	0.036	0.102	0.035	0.081	0.097	0.279	−0.18
	2	C	0.187	0.019	**0.167**	0.517	0.053	**0.462**	0.443	−0.042	1.226	−0.145	+1.37
*Z*-3c	1	C	0.058	−0.005	0.053	0.17	−0.015	0.155	0.045	0.066	0.132	0.222	±0.18
	2	C	0.180	0.037	**0.143**	0.526	0.108	**0.418**	0.424	−0.027	1.240	−0.091	+1.33
*E*-3c	1	C	0.057	−0.016	0.041	0.168	−0.047	0.121	0.045	0.048	0.133	0.162	±0.15
	2	C	0.182	0.04	**0.142**	0.537	0.118	**0.419**	0.425	−0.022	1.254	−0.074	+1.33
*Z*-3d	1	C	0.065	−0.012	0.054	0.213	−0.039	0.177	0.058	0.059	0.190	0.189	±0.19
	2	C	0.172	0.041	**0.130**	0.564	0.135	**0.427**	0.404	−0.025	1.325	−0.08	+1.41
*E*-3d	1	C	0.064	−0.022	0.042	0.212	−0.073	0.139	0.057	0.041	0.189	0.132	±0.16
	2	C	0.174	0.044	**0.130**	0.577	0.146	**0.431**	0.405	−0.021	1.343	−0.068	+1.41
*Z*-3e	1	C	0.061	−0.013	0.048	0.19	−0.041	0.150	0.045	0.055	0.140	0.178	±0.16
	2	C	0.182	0.043	**0.138**	0.567	0.134	**0.430**	0.431	−0.022	1.343	−0.071	+1.41
*E*-3e	1	C	0.060	−0.025	0.036	0.189	−0.079	0.113	0.045	0.035	0.142	0.114	±0.13
	2	C	0.184	0.046	**0.138**	0.58	0.145	**0.435**	0.432	−0.017	1.362	−0.055	+1.41
*Z*-3f	1	C	0.078	−0.032	0.046	0.312	−0.128	0.184	0.082	0.040	0.328	0.120	+0.21
	2	C	0.140	0.058	**0.083**	0.561	0.232	**0.332**	0.332	−0.016	1.329	−0.048	+1.38
*E*-3f	1	C	0.077	−0.041	0.036	0.312	−0.166	0.146	0.082	0.024	0.332	0.073	+0.26
	2	C	0.142	0.059	**0.083**	0.576	0.239	**0.336**	0.332	−0.014	1.346	−0.042	+1.39
*Z*-3g	1	C	0.082	−0.041	0.041	0.363	−0.182	0.182	0.079	0.031	0.350	0.092	+0.26
	2	C	0.108	0.066	**0.042**	0.479	0.293	**0.186**	0.271	−0.013	1.201	−0.039	+1.24
*E*-3g	1	C	0.080	−0.048	0.033	0.359	−0.215	0.148	0.079	0.017	0.354	0.051	+0.30
	2	C	0.110	0.066	**0.044**	0.493	0.296	**0.197**	0.272	−0.012	1.220	−0.036	+1.26

a Calculations based on the method of DFT were performed at the B3LYP/cc-pVTZ level of the theory.

The Fukui dual descriptor *f*^(2)^_k_/multiphilic descriptor Δ*ω*_k_ of DF (4) are −1.697/−0.334 for (C3) and 0.088/0.174 (N5), respectively. While these for APPs (*E*/*Z*)-3a–g are 0.029–0.054/0.079–0.177 (C1) and 0.042–0.167/0.186–0.462 (C2), respectively, indicating that C3 is the most nucleophilic center in DF and C2 is the most electrophilic site of APPs.

**Fig. 4:**
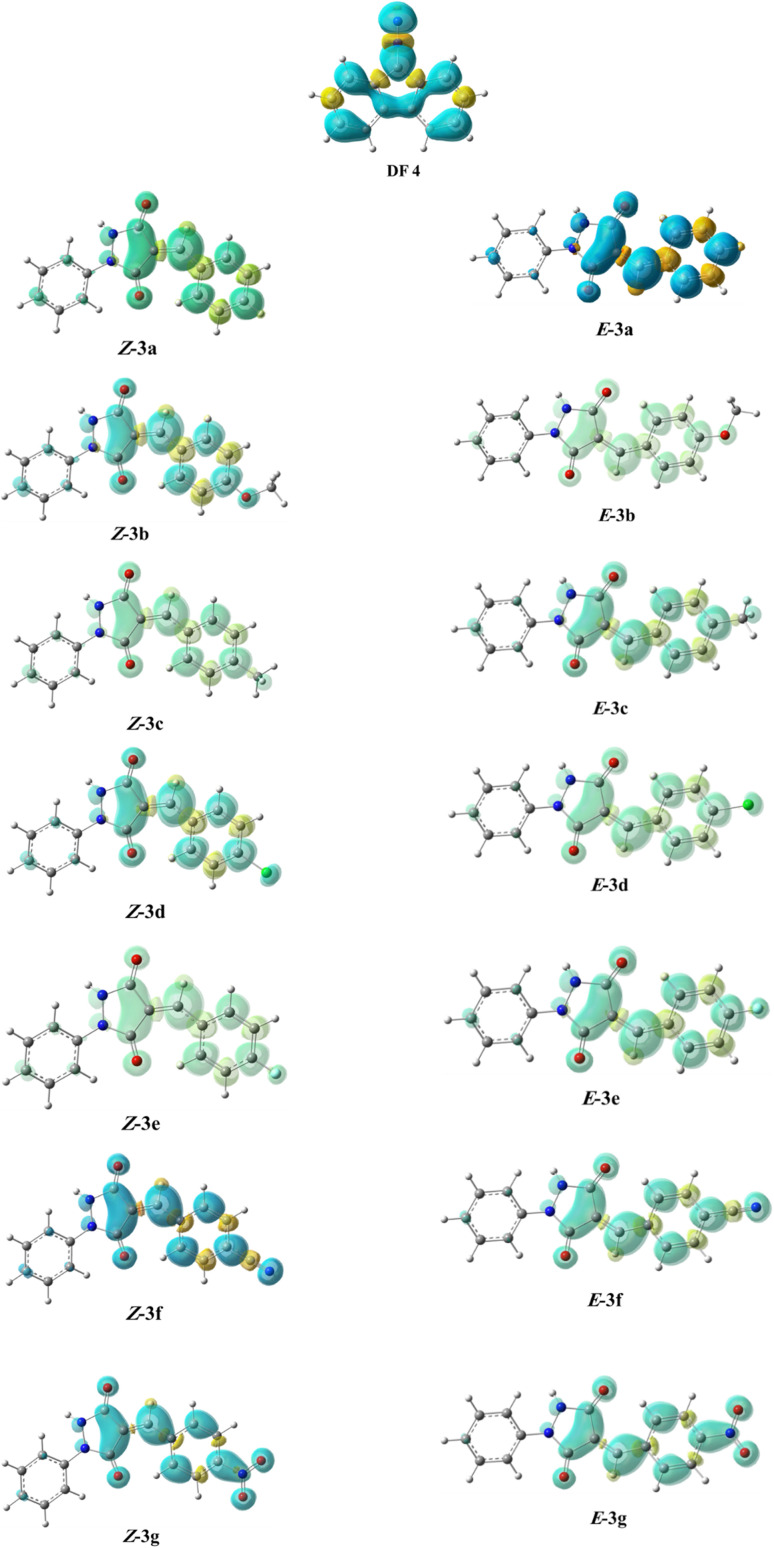
ASD maps of the radical cation of DF 4 and radical anions of APPs 3a–g at B3LYP/ccpVTZ.

On the other hand, local electrophilic *P*_k_^+^ and nucleophilic *P*_k_^−^ Parr functions, which are obtained from the atomic spin density (ASD) at the radical anion and at the radical cation of the corresponding reagents, respectively, can be used to define local electrophilicity *ω*_k_ = *ω P*_k_^+^, local nucleophilicity *N*_k_ = *N P*_k_^−^ indices,^40^ which are summarized in Table 5, and provide further insight into the regiochemistry of our 32CA reaction of DF with APPs. Fig. 4 shows the ASD maps of the radical cation of DF (4) and radical anions of APPs (*E*/*Z*)-3a–g calculated at the B3LYP/cc-pVTZ level.

To acquire a deeper insight into nucleophilic and/or electrophilic activation at the different sites of a molecule, Chattaraj *et al.* proposed that the local reactivity difference index *R*_k_, can predict the local nucleophilic and/or electrophilic activation within an organic molecule.^41^

The *R*_k_ index is described as:If (1 < *ω*_k_/*N*_k_ < 2 or 1 < *N*_k_/*ω*_k_ < 2), then *R*_k_ ≈ (*ω*_k_ + *N*_k_)/2 ⇒ corresponds to ambiphilic site (*R*_k_ = ± x.xx)then, *R*_k_ ≈ (*ω*_k_ − *N*_k_); where *R*_k_ < 0 ⇒ corresponds to nucleophilic site (*R*_k_ = −x.xx)*R*_k_ > 0 ⇒ corresponds to electrophilic site (*R*_k_ = +x.xx).

Parr indices *P*_k_^±^, local electrophilicity *ω*_k_, local nucleophilicity *N*_k_ and local reactivity difference index *R*_k_ for the most relevant heavy atoms of DF (4) and APPs (*E*/*Z*)-3a–g are listed in Table 5.

From the data presented in Table 5 for DF, the C3 site is more nucleophilic (*R*_k_ = −0.97), while N5 is classified as an ambiphilic site (*R*_k_ = ±1.35). On the other hand, for APPs (*E*/*Z*)-3a–g, the C2 site is more electrophilic than C1 (C2, *R*_k_ = +1.24 − +1.41; C1, *R*_k_ = −0.23 − +0.30).

Therefore, the most favorable regioisomeric pathway is accompanied by initial C^DF^_3_–C^APP^_2_ bond formation. These results are consistent with experimental findings indicating that the 32CA reaction between DF (4) and APPs (*E*/*Z*)-3a–g occurs through concerted interactions involving C^DF^_3_–C^APP^_2_ and N^DF^_5_–C^APP^_1_ bond formation, leading to the generation of the spiropyrazole derivatives 5/5′a–g that facilitate path A (β-attack) (Scheme 4). Overall, the combined local reactivity analysis is fully consistent with the experimentally observed regioselective formation of 5/5′a–g. Taken together, the computational data provide a coherent picture of the observed selectivity. The frontier-orbital energy gaps indicate a normal electron-demand cycloaddition, while the global reactivity indices identify DF as the nucleophilic partner and the APP derivatives as the electrophilic dipolarophiles. The local descriptors further show that the C3 center of DF is the most nucleophilic site and the C2 position of the dipolarophile is the most electrophilic site, supporting the experimentally observed C^DF^_3_–C^APP^_2_ bond formation pattern. In addition, the transition-state analysis shows that the *endo* pathway leading to products 5/5′ is energetically preferred over the alternative pathways, in agreement with the exclusive formation of one regioisomeric framework in the experimental study. Although the present computational study provides a consistent and reliable description of the observed regioselectivity, explicit inclusion of solvent effects (*e.g.*, PCM models) and detailed comparison of activation free energies for *endo* and *exo* pathways would further refine the mechanistic analysis. Such investigations are computationally demanding and will be the subject of future studies.

In addition to the local electrophilicity- and nucleophilicity-based descriptors discussed above, the hard and soft acids and bases (HSAB) principle^42^ and the local softness *s*_k_^±^ can be used to predict the regioselectivity of 32CA reactions.^43^ The local softness values *s*_k_^±^ are calculated by *s*_k_^±^ = *S*.*f*_k_^±^,^44^ where *S* is the global softness and *f*_k_^±^ are the respective Fukui functions. Following the above definition, the associated dual local softness (Δ*s*_k_) has also been defined as the condensed version of *f*^(2)^_k_ multiplied by the global softness; Δ*s*_k_ = *S*.*f*^(2)^_k_ = (*s*_k_^+^ − *s*_k_^−^).^45^

Although local hardness descriptors may occasionally exhibit ambiguity, dual local softness provides a more reliable description of hard–soft interactions at the local level. In this context, Ayres provided a rigorous theoretical foundation for the global and local HSAB principles and clarified their relationships with polarizability, charge, and electronegativity.^46^ However, the dual descriptor *f*^(2)^_k_ is a sub-intensive property, meaning that its condensed values become less significant as the molecular size increases. To overcome this intrinsic behavior of this local reactivity descriptor, another local reactivity descriptor has been defined as the local hypersoftness (*s*^(2)^_k_), which allows local reactivities to be evaluated for molecular size and is calculated by *s*^(2)^_k_ = *S*^2^(*f*_k_^+^ − *f*_k_^−^) = *S*^2^.*f*^(2)^_k_.^47^

Accordingly, local softness *s*_k_^±^, dual local softness Δ*s*_k_, and local hypersoftness *s*^(2)^_k_ for the most relevant heavy atoms of DF (4) and APPs (*E*/*Z*)-3a–g are shown in Table 6.

**Table 6:** Local softness *s*_k_^±^, dual local softness Δ*s*_k_, and local hypersoftness *s*^(2)^_k_ for the most relevant heavy atoms of DF (4) and APPs 3a–g^a^

Reactant	Site k		*s*_k_^+^	*s*_k_^−^	Δ*s*_k_	*s* ^(2)^_k_
4	3	C	0.079	1.299	**−1.220**	−8.805
	4	N	1.516	−0.123	1.393	10.056
	5	N	2.165	1.530	0.635	4.585
*Z*-3a	1	C	0.523	−0.158	0.365	3.028
	2	C	1.493	0.407	**1.087**	9.016
*E*-3a	1	C	0.519	−0.243	0.276	2.313
	2	C	1.524	0.427	**1.097**	9.183
*Z*-3b	1	C	0.414	0.171	0.236	1.913
	2	C	1.495	0.138	**1.357**	11.019
*E*-3b	1	C	0.409	0.106	0.302	2.470
	2	C	1.528	0.155	**1.365**	11.151
*Z*-3c	1	C	0.477	−0.041	0.436	3.582
	2	C	1.480	0.304	**1.176**	9.665
*E*-3c	1	C	0.473	−0.133	0.340	2.822
	2	C	1.510	0.332	**1.178**	9.773
*Z*-3d	1	C	0.549	−0.101	0.456	3.857
	2	C	1.454	0.346	**1.099**	9.284
*E*-3d	1	C	0.546	−0.188	0.358	3.056
	2	C	1.484	0.375	**1.109**	9.459
*Z*-3e	1	C	0.505	−0.108	0.397	3.283
	2	C	1.505	0.356	**1.141**	9.440
*E*-3e	1	C	0.502	−0.209	0.301	2.523
	2	C	1.541	0.385	**1.155**	9.673
*Z*-3f	1	C	0.707	−0.290	0.417	3.784
	2	C	1.270	0.526	**0.753**	6.828
*E*-3f	1	C	0.708	−0.377	0.331	3.042
	2	C	1.305	0.542	**0.763**	7.014
*Z*-3g	1	C	0.786	−0.393	0.393	3.764
	2	C	1.035	0.632	**0.402**	3.856
*E*-3g	1	C	0.775	−0.465	0.320	3.094
	2	C	1.065	0.639	**0.426**	4.126

a Calculations based on the method of DFT were performed at the B3LYP/cc-pVTZ level of the theory.

Building on the foregoing discussion of local softness descriptors, dual local softness and local hypersoftness can be employed to identify the most reactive atomic sites toward electrophilic attack. From Table 6, the negative condensed values of dual local softness and local hypersoftness are observed DF at C3 (−1.220 and −8.805, respectively), indicating that this position is the most favorable electron-donating site in DF. In contrast, for the APPs, C2 exhibits more positive values of dual local softness and local hypersoftness (0.402–1.365 and 3.856–11.151, respectively) than C1 (0.236–0.456 and 1.913–3.857, respectively), thereby identifying C2 as the most favorable electron-accepting site among all APPs examined in this study. These findings are fully consistent with the experimental results of the 32CA reaction of DF (4) and APPs (*E*/*Z*)-3a–g, which proceeds preferentially through path A (β-attack) to afford spiropyrazole derivatives 5/5′a–g (Scheme 4).

Taken together, and in the context of the foregoing local reactivity analyses, the Fukui dual descriptor *f*^(2)^_k_, multiphilic descriptor Δ*ω*_k_, local reactivity difference index *R*_k_, and dual local softness Δ*s*_k_, provide a consistent and complementary framework. All these descriptors clearly distinguish between nucleophilic and electrophilic attacks at a particular site through the sign of their values. That is, they provide a negative value for sites favored for electrophilic attack and a positive value for sites favored for nucleophilic attack. These results were found to be in excellent agreement with the experimental observations, thus confirming the accuracy of the calculated dual descriptors for the local electrophilicity, nucleophilicity, and softness of the reactants. Accordingly, by using these descriptors, the observed regioselective behavior of DF and APPs in the 32CA reaction can be rationalized, providing deeper insight into the underlying mechanism of regioselectivity.

#### Electrostatic potential distribution (ESP)

**Fig. 5:**
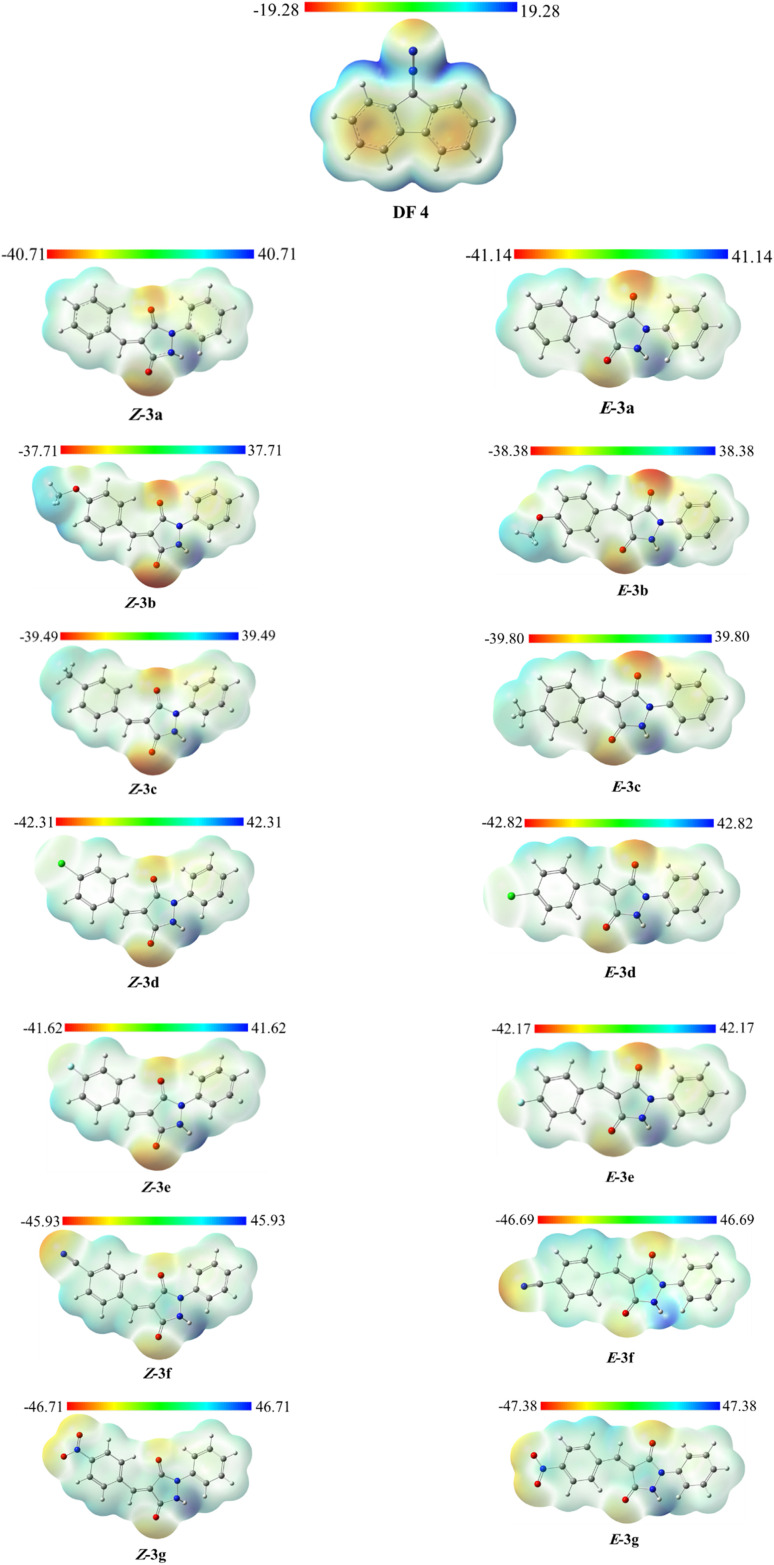
Electrostatic potential (ESP) maps of DF (4) and APPs (*E*/*Z*)-3a–g (B3LYP/cc-pVTZ, kcal mol^−1^).

To further complement the local reactivity descriptors discussed above and to provide a spatial visualization of charge distribution, quantitative molecular surface analysis can be performed using the molecular surface as a simulation technique, which enables the visualization of the electron density distribution.^48^ The electrostatic potential map helps analyze the distribution of charges in various types of molecules, including organic compounds. The molecular electrostatic potential (MESP) has recently been utilized to determine the most likely reactive sites in cycloaddition reactions. Beyond qualitative interpretation, electrostatic effects can also play a decisive role in governing regioselectivity.^49^ The electrostatic potential distribution patterns of the starting materials DF (4) and APPs (*E*/*Z*)-3a–g are shown in Fig. 5. The molecular surfaces were generated using an isosurface of electron density *ρ* = 0.001 a.u., and the color scale is expressed in kcal mol^−1^. The blue color represents regions of positive electrostatic potential, indicating sites susceptible to nucleophilic attack, whereas the red color corresponds to regions of negative electrostatic potential, indicating sites prone to electrophilic attack. From Fig. 5, it is evident that C2 centers in APPs (*E*/*Z*)-3a–g are located in the regions of positive electrostatic potential, confirming their higher electrophilicity and identifying them as the preferred sites for nucleophilic attack compared to the C1 centers. This observation supports the formation of the regioisomeric adducts 5/5′ as the major products *via* path A (β-attack).

#### Study of energies of transition-state structures

**Fig. 6:**
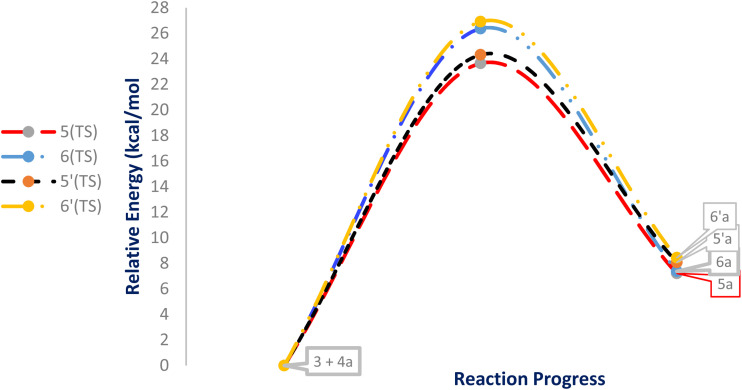
Relative energies (kcal mol^−1^) for the reactants (4 + 3a), TSs, and products for the four possible reaction pathways.

While the FMO analysis provides valuable insight into the electronic factors governing regioselectivity, it does not explicitly account for steric effects, which often play a decisive role in controlling the regiochemistry of 32CA reactions.^50^ Experimentally, it was observed that the cycloaddition reactions of DF (4) and APPs (*E*/*Z*)-3a–g are completely regiospecific, with no formation of the alternative regioisomeric products 6′/6 detected. Instead, only a single regioisomer 5′/5 was obtained through path A (β-attack). Hypothetically, the reaction may proceed *via* either path A (β-attack) or path B (α-attack), and the relative orientation of the reactants can adopt either *endo* or *exo* approaches (Scheme 4). Accordingly, a theoretical study was carried out to evaluate the energetics of reaction paths A and B for both *endo* and *exo* transition states, leading to the possible formation of regioisomers 5/5′ and 6/6′ from DF (4) and APPs (*E*/*Z*)-3a–g (Fig. 6).

To further elucidate the origin of the experimentally observed stereoselectivity, the reaction of DF (4) and APPs (*E*/*Z*)-3a–g was analyzed by comparing the activation and reaction energies, enthalpies, and Gibbs free energies associated with the possible reaction pathways leading to the stereoisomeric products 5/5′ and 6/6′*via* the *endo*- and *exo*-TS, respectively (Table 7).

**Table 7:** Calculated electronic activation energies *E*_a_, reaction enthalpies Δ*H*, reaction Gibbs free energies Δ*G*, reaction energies Δ*E*_rxn_, activation enthalpies Δ*H*^#^, activation Gibbs free energies Δ*G*^#^ (in kcal mol^−1^) and reaction entropies Δ*S* (in cal mol K^−1^), and bond length differences (Å) for the cycloaddition reaction of DF 4 and APP 3a at B3LYP/cc-pVTZ level

Structure	*E*_a_	Δ*H*	Δ*G*	Δ*E*_rxn_	Δ*H*^#^	Δ*G*^#^	Δ*S*	Δ*R*
5(TS)	**23.65**	**9.25**	**24.48**	**7.21**	**24.04**	**38.26**	**−51.08**	0.38
5′(TS)	**24.32**	**10.04**	**25.19**	**8.08**	**24.69**	**38.50**	**−50.81**	0.36
6(TS)	26.37	9.14	24.24	7.37	26.61	40.83	−50.65	0.81
6′(TS)	26.91	10.13	25.05	8.44	27.19	41.52	−50.06	0.81

In the gas phase, the calculated relative Gibbs free energies of activation for the possible pathways of this 32CA reaction at the B3LYP/cc-pVTZ level are 38.26 kcal mol^−1^ for 5(TS), 38.50 kcal mol^−1^ for 5′(TS), 40.83 kcal mol^−1^ for 6(TS), and 41.52 kcal mol^−1^ for 6′(TS). Consistent with these trends, the calculated free activation energies for the formation of *endo* regioisomer 5/5′*via* β-attack (23.65/24.32 kcal mol^−1^) are systematically lower than that of *exo* regioisomer 6/6′*via* α-attack (26.37/26.91 kcal mol^−1^). These energetic differences indicate that *endo* adducts 5/5′ are preferentially formed, accounting for the high stereo- and regioselectivity observed experimentally, as the *endo* transition states are energetically favored relative to the corresponding *exo* transition states.

**Fig. 7:**
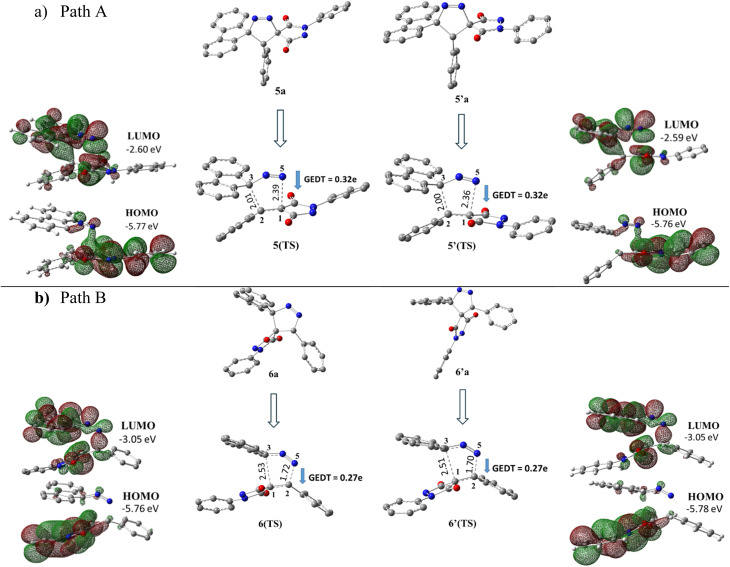
Optimized products and transition state geometries, HOMO and LUMO energies, and global electron density transfer at (a) *endo*-TS and (b) *exo*-TS in the cycloaddition reaction of DF (4) with APP (*E*/*Z*)-3a in possible paths A and B at the B3LYP/cc-pVTZ level. The C3⋯C2/N5⋯C1 (path A) and C3⋯C1/N5⋯C2 (path B) bond distance (*A*°) are shown in dashed lines. The blue arrow indicates the direction of GEDT.

In addition to energetic considerations, Table 7 also reports the bond length differences (Δ*R*) between the two forming bonds in the corresponding transition states. Analysis of these geometrical parameters indicates that regioisomeric path A proceeds through a more synchronous transition state than path B. The optimized geometries of the reactants, transition states, and products for the representative reaction of DF (4) with APP (*E*/*Z*)-3a are depicted in Fig. 7.

The polarity of the reaction also emerges as a significant factor in determining the feasibility of the cycloaddition. The global electron density transfer was computed at the transition states by the sum of the natural atomic charges (*q*), obtained from natural population analysis (NPA), over the atoms belonging to each reacting framework (*f*) at the TSs,^51^ according to 
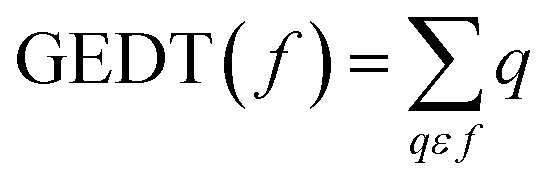
, which reflects electron density flux from DF (4) toward the APP framework. As illustrated in Fig. 7, along the possible reaction pathways, the GEDT values are calculated to be 0.32*e* at 5(TS) and 5′(TS), and 0.27*e* at 6(TS) and 6′(TS). These relatively large GEDT values emphasize the highly polar nature of the reaction.

**Fig. 8:**
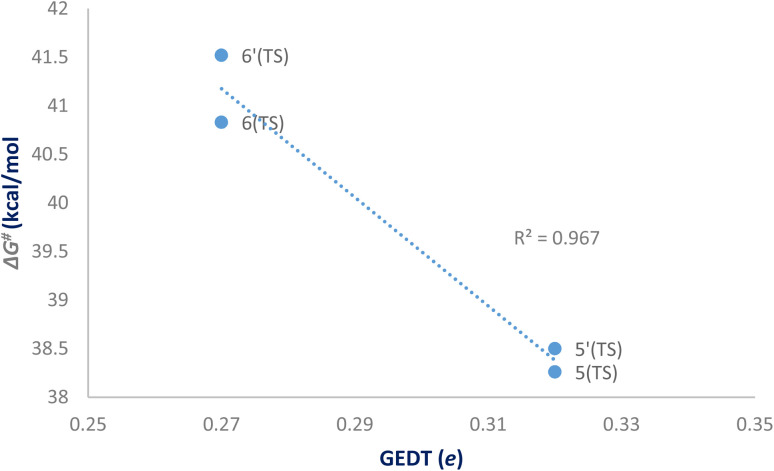
Plot of the activation free energies *vs.* GEDT at TSs for the cycloaddition reaction of DF (4) with APP 3a in possible reaction paths A and B at the B3LYP/cc-pVTZ level.

In line with the foregoing analysis, a good correlation between GEDT values at TS and the estimated relative free energy of activation (Δ*G*^#^) can be established, with *R*^2^ = 0.97. This correlation is consistent with the notion that higher GEDT values facilitate bond formation, resulting in lower activation free energies and faster reaction rates,^51^ in good agreement with the experimental observations (Fig. 8). The lower *R*^2^ reflects limitations of correlating computed descriptors with experimental data but does not affect qualitative conclusions.

**Fig. 9:**
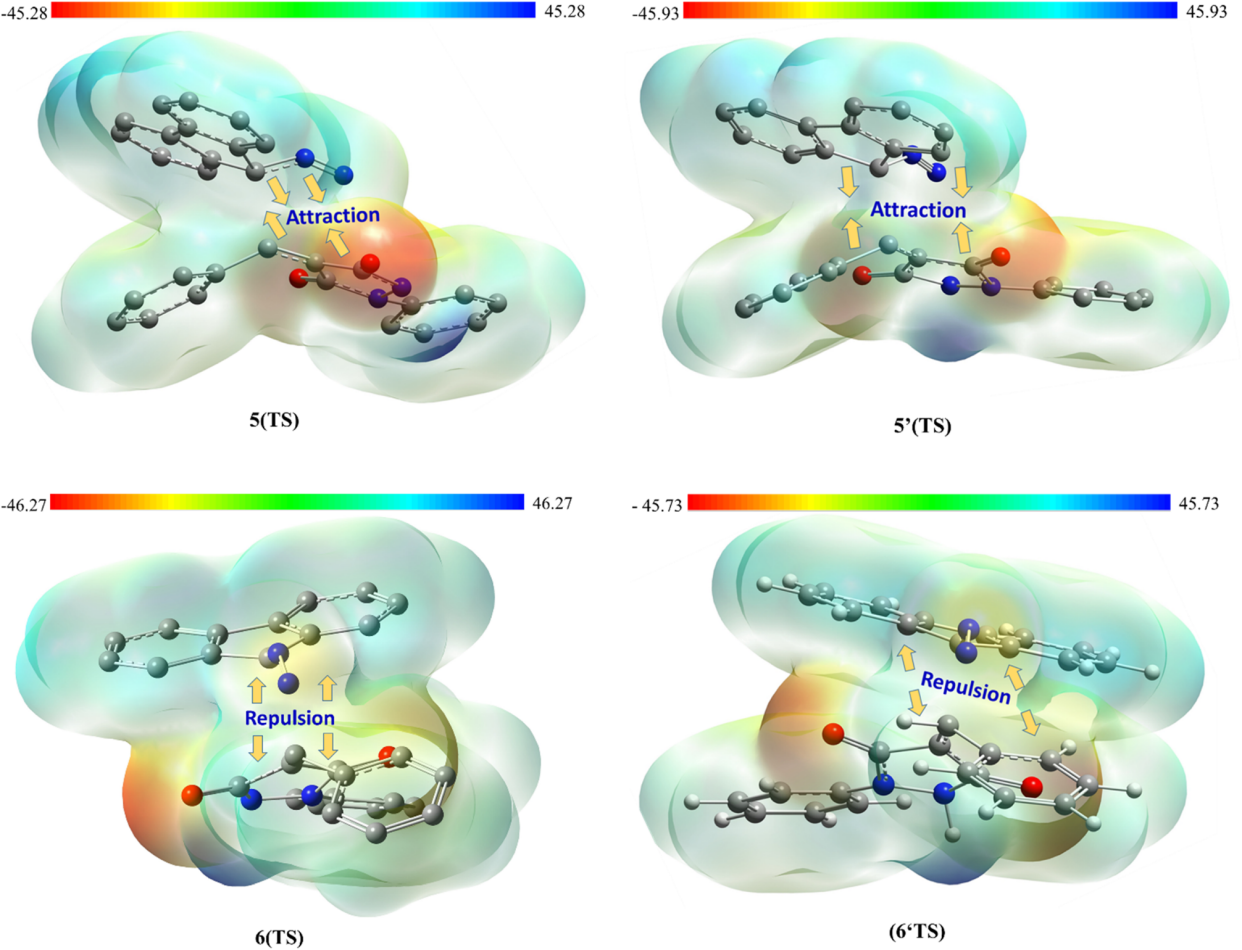
Molecular electrostatic potential MESP maps of the transition states associated with the possible regioisomeric attacks. The red and blue colors display high and low electron density regions, respectively (*ρ* = 0.001 at B3LYP/cc-pVTZ, kcal mol^−1^).

Additionally, the observed stereochemistry can be explained by assessing the molecular electrostatic potential surface (MESP) and analyzing the associated electrostatic interactions. Fig. 9 demonstrates that in the energetically favorable transition states 5(TS) and 5′(TS), the approach of the reactants aligns oppositely charged regions, giving rise to attractive electrostatic interactions between the two reacting fragments. In contrast, in the energetically less favorable transition states 6(TS) and 6′(TS), regions bearing like charges are brought into proximity, leading to repulsive electrostatic interactions between the reactants. Consequently, the presence of favorable electrostatic attractions in 5(TS) and 5′(TS) renders their formation more favorable than that of 6(TS) and 6′(TS), fully consistent with the experimentally observed stereochemical outcome.

## Conclusions

In conclusion, we have developed a regio- and stereoselective method for the synthesis of novel dispiro[fluorene-9,3′-pyrazole-5′,4″-pyrazolidines] *via* a one-pot [3 + 2] cycloaddition (32CA) reaction of 9-diazo-9*H*-fluorene (DF) with a series of (*E*/*Z*)-4-arylidene-1-phenylpyrazolidine-3,5-diones (APPs). The obtained products were characterized by 1D and 2D-NMR spectroscopy, confirming their regiochemical and stereochemical assignments. The regiospecific cycloaddition reactions consistently proceed *via* path A (β-attack), resulting in formation of a single regioisomer. Complementary computational analysis revealed that the *endo* transition state displays the lowest activation energy among the possible reaction pathways, in agreement with the experimental observations. The electrophilic and nucleophilic properties of DF (4) and APPs (*E*/*Z*)-3a–g involved in the polar cycloaddition reactions were systematically investigated using global and dual local electrophilicity and nucleophilicity indices, together with dual local softness and local hypersoftness descriptors, all of which showed close agreement with the experimental findings. In particular, analysis of the dual local reactivity indices indicated that the C3 carbon is the most nucleophilic center of DF (4), while the C2 site of APPs 3 is the most electrophilic site. These findings were consistent with the experimental observations, which established that the [3 + 2] cycloaddition reaction between DF (4) and APPs (*E*/*Z*)-3a–g proceeds *via* concerted interactions between C^DF^_3_–C^APP^_2_ and N^DF^_5_–C^APP^_1_, leading to the formation of the spiropyrazole derivatives 5/5′a–g through path A (β-attack). The global electron density transfer analysis further confirms the polar nature of the reactions, with electron density flux from DF (4) toward APPs (*E*/*Z*)-3a–g. Finally, the molecular electrostatic potential surface (MESP) analysis showed that, in the energetically favorable transition states 5(TS) and 5′(TS), the approach of the reactants aligns oppositely charged regions, resulting in attractive electrostatic interactions between the reacting fragments, fully consistent with the observed regio- and stereochemical outcome.

## Experimental section

All solvents purchased from Sigma-Aldrich are spectroscopic grade and used without further purification. NMR spectra were recorded on a Bruker Avance III HD NMR spectrometer (600 MHz for ^1^H, 150 MHz for ^13^C) in DMSO-d_6_ solutions, with residual solvent signals as internal standard. The reactions described in this study were carried out on a 1.0 mmol scale, which provided clean conversions and reproducible isolated yields under the optimized conditions. Although larger-scale reactions were not systematically examined in the present work, the operational simplicity of the protocol, the complete regioselectivity observed across the substrate series, and the direct isolation of the products by filtration suggest that the method should be amenable to further scale-up. A dedicated investigation of preparative-scale synthesis will be undertaken in future studies. The use of readily available starting materials and mild reaction conditions contributes to the practical and potentially cost-effective nature of the developed protocol. The reactions proceeded cleanly under the optimized conditions, and no significant impurities were observed beyond the expected diastereomeric mixtures. Caution: Diazo compounds should be handled with appropriate safety precautions due to their potential instability and reactivity. All reactions were carried out using standard safety protocols and under controlled conditions.

### Computational details

Geometry optimizations were computed in vacuum with density functional theory (DFT) using B3LYP exchange correlation functional^53^ combined with Dunning's correlation consistent triple-*ζ* basis set (cc-pVTZ). Regional Fukui functions for electrophilic (*f*_k_^−^) and nucleophilic (*f*_k_^+^) attacks were calculated employing the natural population analysis (NPA) at the B3LYP/cc-pVTZ level of theory.^54^ The electrophilic, (*P*_k_^+^), and nucleophilic, (*P*_k_^−^), Parr functions were obtained through the analysis of the Mulliken ASD of the radical anion and the radical cation by single-point energy calculations over the optimized neutral geometries using the unrestricted UB3LYP/cc-pVTZ formalism for radical species.^40*d*^

### Synthesis of (*E*/*Z*)-4-arylidene-1-phenylpyrazolidine-3,5-diones (APPs) 3a–g

APPs 3a–g were prepared according to the previously reported procedure,^17*a*,*b*^ isolated as (*E*/*Z*)-diastereomeric mixture in a (≈1 : 1 ratio), and cannot be separated due to their similar *R*_f_.

#### (*E*)/(*Z*)-4-(4-Benzylidene)-1-phenylpyrazolidine-3,5-dione (3a)


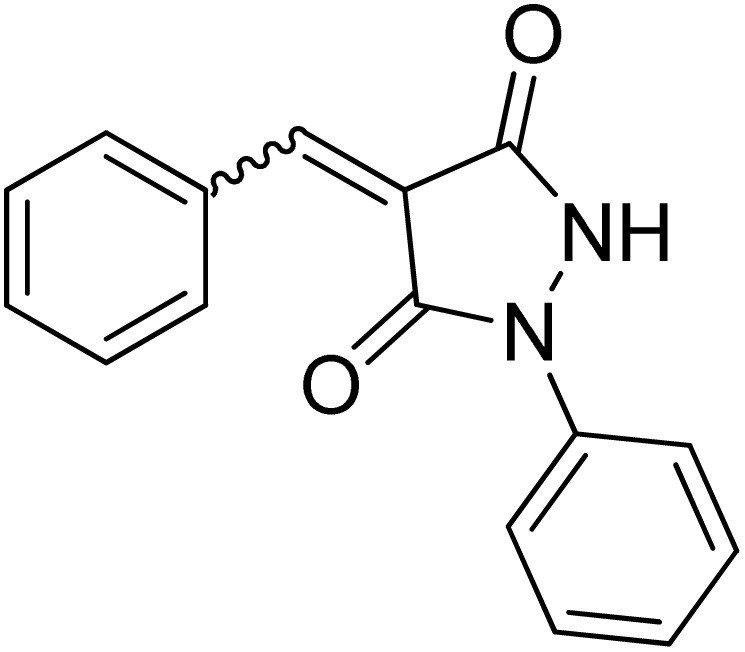
 ^1^H NMR (DMSO-d_6_, 600 MHz) *δ* 11.34 (br s, 1H, NH), 8.57 (d, *J* = 7.8 Hz, 1H, *meta* CH_Ph_), 8.51 (br s, 1H, *meta* 2xCH_Ph_), 7.93 & 7.88 (2xbr s, 1H, CH_benzylidene_), 7.78–7.70 (m, 2H, 2xCH_N-Ph_), 7.66–7.61 (m, 1H, *para* CH_Ph_), 7.59–7.53 (m, 2H, *ortho* CH_Ph_), 7.47 (d, *J* = 7.8 Hz, 1H, 2xCH_*N*-Ph_), 7.44 (d, *J* = 7.2 Hz, 1H, 2xCH_*N*-Ph_), 7.21 (t, *J* = 7.2 Hz, 1H, *para* CH_*N*-Ph_); ^13^C NMR (DMSO-d_6_, 151 MHz) *δ* 160.6 (C=O), 158.8 (C=O), 134.3 (CH_benzylidene_), 134.2 (CH_benzylidene_), 133.7 (CH_Ph_), 132.5 (N–C_q_), 132.4 (N–C_q_), 129.2 (*ortho* 2xCH_Ph_), 129.1 (*ortho* 2xCH_Ph_), 129.0 (*meta* 2xCH_Ph_), 128.9 (*meta* 2xCH_Ph_), 125.0 (*meta* 2XCH_*N*-Ph_), 120.0 (C_*q*-pyrazolidinone_), 119.8 (C_q-Ph_), 119.1 (*para* CH_*N*-Ph_), 118.5 (*ortho* 2xCH_*N*-Ph_).

#### (*E*)/(*Z*)-4-(4-Methoxybenzylidene)-1-phenylpyrazolidine-3,5-dione (3b)


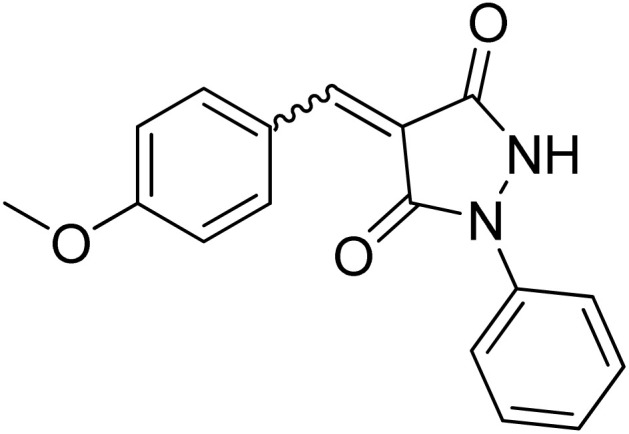
 ^1^H NMR (DMSO-d_6_, 600 MHz) *δ* 11.24 (br s, 1H, NH), 8.67 (d, *J* = 9.0 Hz, 1H, *meta* CH_methoxyphenyl_), 8.63 (br s, 1H, *meta* 2xCH_methoxyphenyl_), 7.87 & 7.83 (2xbr s, 1H, CH_benzylidene_), 7.79–7.67 (m, 2H, 2xCH_*N*-Ph_), 7.50–7.40 (m, 2H, 2xCH_*N*-Ph_), 7.20 (t, *J* = 7.2 Hz, 1H, *para* CH_*N*-Ph_), 7.15–7.07 (m, 2H, *ortho* CH_methoxyphenyl_), 3.88 (s, 3H, CH3); ^13^C NMR (DMSO-d_6_, 151 MHz) *δ* 164.0 (C–O), 163.9 (C–O), 161.1 (C=O), 159.3 (C=O), 137.4 (CH_benzylidene_), 137.3 (CH_benzylidene_), 129.0 (*meta* 2xCH_methoxyphenyl_), 128.9 (*meta* 2xCH_methoxyphenyl_), 125.6 (2 signals, N–C_q_), 124.7 (*meta* 2XCH_*N*-Ph_), 118.9 (*para* CH_*N*-Ph_), 118.3 (*ortho* 2xCH_*N*-Ph_), 116.4 (C_*q*-pyrazolidinone_), 116.1 (C_*q*-methoxyphenyl_), 114.6 (*ortho* 2xCH_tolyl_), 114.5 (*ortho* 2xCH_methoxyphenyl_), 55.8 (OCH_3_).

#### (*E*)/(*Z*)-4-(4-Methylbenzylidene)-1-phenylpyrazolidine-3,5-dione (3c)


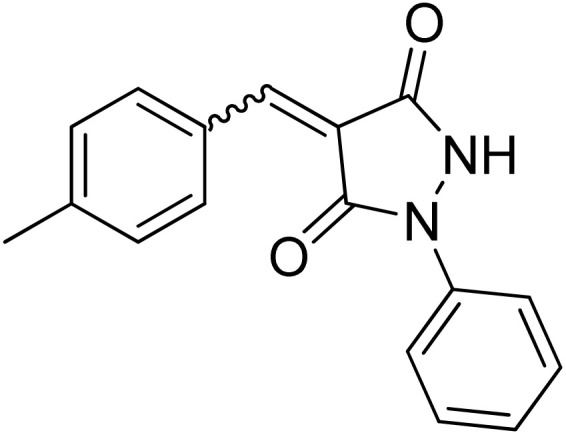
 ^1^H NMR (DMSO-d_6_, 600 MHz) *δ* 11.36 (br s, 1H, NH), 8.51 (d, *J* = 8.4 Hz, 1H, *meta* CH_*p*-tolyl_), 8.46 (br s, 1H, *meta* 2xCH_*p*-tolyl_), 7.88 & 7.84 (2xbr s, 1H, CH_benzylidene_), 7.80–7.67 (m, 2H, 2xCH_*N*-Ph_), 7.48–7.42 (m, 2H, 2xCH_*N*-Ph_), 7.39–7.35 (m, 2H, *ortho* CH_*p*-tolyl_), 7.21 (t, *J* = 7.2 Hz, 1H, *para* CH_*N*-Ph_), 2.40 (s, 3H, CH_3_); ^13^C NMR (DMSO-d_6_, 151 MHz) *δ* 160.7 (C=O), 158.9 (C=O), 144.7 (CH_3_C), 134.5 (CH_benzylidene_), 134.4 (CH_benzylidene_), 129.9 (2 signals, N-Cq), 129.6 (*ortho* 2xCH_*p*-tolyl_), 129.5 (*ortho* 2xCH_*p*-tolyl_), 129.0 (*meta* 2xCH_*p*-tolyl_), 128.9 (*meta* 2xCH_*p*-tolyl_), 124.8 (*meta* 2XCH_*N*-Ph_), 118.9 (*para* CH_*N*-Ph_), 118.7 (C_*q*-pyrazolidinone_), 118.5 (C_*q*-tolyl_), 118.4 (*ortho* 2xCH_*N*-Ph_), 21.6 (CH_3_).

#### (*E*)/(*Z*)-4-(4-Chlorobenzylidene)-1-phenylpyrazolidine-3,5-dione (3d)


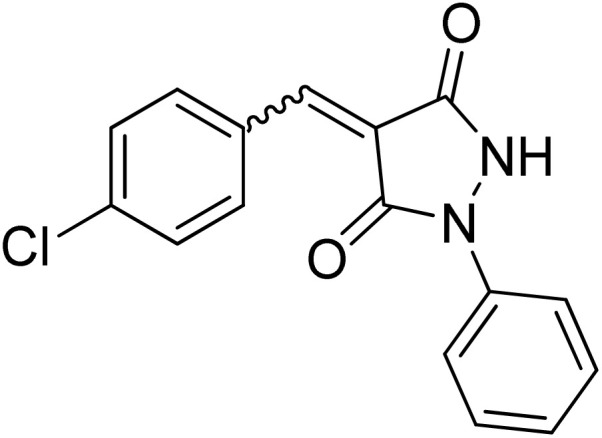
 ^1^H NMR (DMSO-d_6_, 600 MHz) *δ* 11.43 (br s, 1H, NH), 8.62 (d, *J* = 8.4 Hz, 1H, *meta* CH_*p*-chlorophenyl_), 8.56 (br s, 1H, *meta* 2xCH_*p*-chlorophenyl_), 7.92 & 7.88 (2xbr s, 1H, CH_benzylidene_), 7.80–7.70 (m, 2H, 2xCH_*N*-Ph_), 7.64 (d, *J* = 8.4 Hz, 1H, *ortho* CH_*p*-chlorophenyl_), 7.63 (d, *J* = 8.4 Hz, 1H, *ortho* CH_*p*-chlorophenyl_), 7.47 (d, *J* = 7.8 Hz, 1H, 2xCH_*N*-Ph_), 7.44 (d, *J* = 7.8 Hz, 1H, 2xCH_*N*-Ph_), 7.22 (t, *J* = 7.2 Hz, 1H, *para* CH_*N*-Ph_); ^13^C NMR (DMSO-d_6_, 151 MHz) *δ* 160.2 (C=O), 158.6 (C=O), 138.2 (C–Cl), 135.8 (CH_benzylidene_), 135.7 (CH_benzylidene_), 131.3 (N–C_q_), 131.2 (N–C_q_), 129.1 (*ortho* 2xCH_*p*-chlorophenyl_), 129.0 (*ortho* 2xCH_*p*-chlorophenyl_), 128.9 (2 signals, *meta* 2xCH_*p*-chlorophenyl_), 125.0 (*meta* 2XCH_*N*-Ph_), 120.5 (*para* CH_*N*-Ph_), 120.3 (C_*q*-pyrazolidinone_), 118.9 (C_*q*-*p*-chlorophenyl_), 118.4 (*ortho* 2xCH_*N*-Ph_).

#### (*E*)/(*Z*)-4-(4-Fluorobenzylidene)-1-phenylpyrazolidine-3,5-dione (3e)


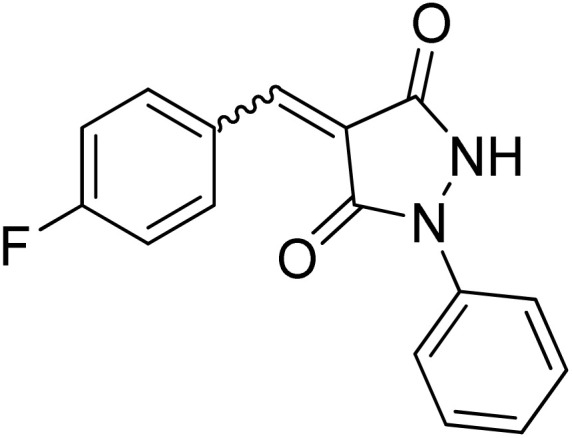
 ^1^H NMR (DMSO-d_6_, 600 MHz) *δ* 11.43 (br s, 1H, NH), 8.74–8.70 (m, 1H, *meta* CH_*p*-fluorophenyl_), 8.67 (br s, 1H, *meta* 2xCH_*p*-fluorophenyl_), 7.94 & 7.89 (2xbr s, 1H, CH_benzylidene_), 7.79–7.69 (m, 2H, 2xCH_*N*-Ph_), 7.50–7.43 (m, 2H, 2xCH_*N*-Ph_), 7.42–7.37 (m, 2H, *ortho* CH_*p*-fluorophenyl_), 7.21 (t, *J* = 7.2 Hz, 1H, *para* CH_*N*-Ph_); ^13^C NMR (DMSO-d_6_, 151 MHz) *δ* 165.0 (d, *J* = 255.2 Hz, C–F), 164.9 (d, *J* = 255.2 Hz, C–F), 160.5 (C=O), 158.8 (C=O), 137.4 (CH_benzylidene_), 137.3 (CH_benzylidene_), 129.3 (2 signals, N–C_q_), 129.1 (*meta* 2xCH_*p*-fluorophenyl_), 129.0 (*meta* 2xCH_*p*-fluorophenyl_), 125.0 (*meta* 2XCH_*N*-Ph_), 119.5 (C_*q*-pyrazolidinone_), 119.3 (2 signals, C_*q*-*p*-fluorophenyl_), 119.0 (*para* CH_*N*-Ph_), 118.5 (*ortho* 2xCH_*N*-Ph_), 116.1 (d, *J* = 21.9 Hz, *ortho* 2xCH_*p*-fluorophenyl_), 116.0 (d, *J* = 21.9 Hz, *ortho* 2xCH_*p*-fluorophenyl_). 19F NMR (DMSO-d_6_, 565 MHz) *δ* – 103.9.

#### (*E*)/(*Z*)-4-(4-Cyanobenzylidene)-1-phenylpyrazolidine-3,5-dione (3f)


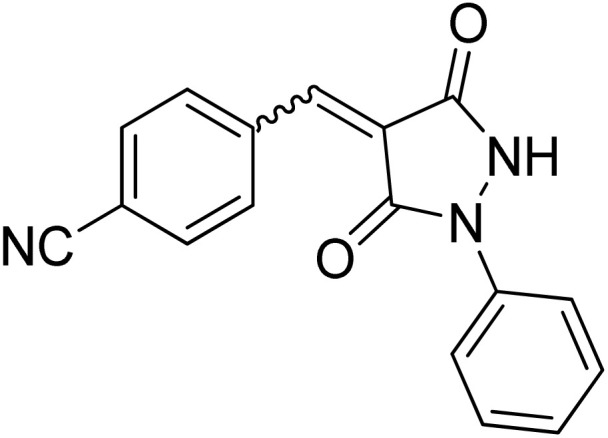
 ^1^H NMR (DMSO-d_6_, 600 MHz) *δ* 10.09 (br s, 1H, NH), 8.63 (d, *J* = 8.4 Hz, 1H, *meta* CH_*p*-cyanophenyl_), 8.57 (d, *J* = 7.8 Hz, 1H, *meta* 2xCH_*p*-cyanophenyl_), 8.00 (d, *J* = 8.4 Hz, 1H, *ortho* CH_*p*-cyanophenyl_), 7.99 (d, *J* = 8.4 Hz, 1H, *ortho* CH_*p*-cyanophenyl_), 7.96 & 7.92 (2xbr s, 1H, CH_benzylidene_), 7.73 (t, *J* = 7.8 Hz, 2H, *meta* 2xCH_*N*-Ph_), 7.48 (d, *J* = 7.2 Hz, 1H, *ortho* 2xCH_*N*-Ph_), 7.45 (d, *J* = 7.2 Hz, 1H, *ortho* 2xCH_*N*-Ph_), 7.22 (t, *J* = 7.8 Hz, 1H, *para* CH_*N*-Ph_); ^13^C NMR (DMSO-d_6_, 151 MHz) *δ* 160.0 (C=O), 158.3 (C=O), 136.4 (N–C_q_), 134.1 (CH_benzylidene_), 134.0 (CH_benzylidene_), 132.6 (*ortho* 2xCH_*p*-cyanophenyl_), 132.5 (*ortho* 2xCH_*p*-cyanophenyl_), 129.3 (*meta* 2xCH_*p*-cyanophenyl_), 129.2 (*meta* 2xCH_*p*-cyanophenyl_), 125.4 (2 signals, *meta* 2XCH_*N*-Ph_), 123.2 (C_*q*-pyrazolidinone_), 123.0 (C_*q*-cyanophenyl_), 119.3 (*para* CH_*N*-Ph_), 118.8 (*ortho* 2xCH_*N*-Ph_), 118.7 (NC–C), 114.6 (CN).

#### (*E*)/(*Z*)-4-(4-Nitrobenzylidene)-1-phenylpyrazolidine-3,5-dione (3g)


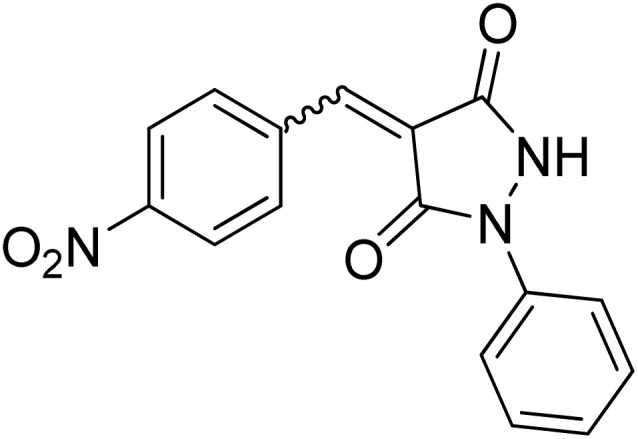
 ^1^H NMR (DMSO-d_6_, 600 MHz) *δ* 10.13 (br s, 1H, NH), 8.68 (d, *J* = 7.8 Hz, 1H, *ortho* CH_*p*-nitrophenyl_), 8.61 (d, *J* = 7.8 Hz, 1H, *ortho* 2xCH_*p*-nitrophenyl_), 8.38–8.28 (m, 2H, *meta* CH_*p*-nitrophenyl_), 7.99 & 7.95 (2xbr s, 1H, CH_benzylidene_), 7.71 (t, *J* = 7.8 Hz, 2H, *meta* 2xCH_*N*-Ph_), 7.47 (d, *J* = 7.2 Hz, 1H, *ortho* 2xCH_*N*-Ph_), 7.44 (d, *J* = 7.2 Hz, 1H, *ortho* 2xCH_*N*-Ph_), 7.23 (t, *J* = 7.8 Hz, 1H, *para* CH_*N*-Ph_); ^13^C NMR (DMSO-d_6_, 151 MHz) *δ* 159.9 (C=O), 158.2 (C=O), 149.3 (2 signals, C–NO_2_), 140.3 (C_*q*-nitrophenyl_), 138.1 (N–C_q_), 134.8 (CH_benzylidene_), 134.7 (CH_benzylidene_), 129.3 (*meta* 2xCH_*p*-nitrophenyl_), 129.2 (*meta* 2xCH_*p*-nitrophenyl_), 124.5 (*meta* 2XCH_*N*-Ph_), 123.7 (*ortho* 2xCH_*p*-nitrophenyl_), 123.6 (*ortho* 2xCH_*p*-nitrophenyl_), 123.5 (C_*q*-pyrazolidinone_), 119.3 (*para* CH_*N*-Ph_), 118.8 (*ortho* 2xCH_*N*-Ph_).

### Synthesis of 9-diazo-9*H*-fluorene (DF)

DF (4) was prepared according to the previously reported procedure.^52^

### Synthesis of dispiro[fluorene-9,3′-pyrazole-5′,4″-pyrazolidines] 5/5′a–g: general procedure

A mixture of the appropriate APPs 3a–g (1.0 mmol), DF (192 mg, 1.0 mmol), and 10 mL of 1,4-dioxane was gently heated under reflux for 60–90 min. Upon completion of the reaction, as monitored by TLC using CH_2_Cl_2_ as the eluent, the mixture was cooled to room temperature. The resultant solid product was filtered off and washed with *n*-hexane to afford the regioisomeric spiro-cycloadducts 5/5′a–g. The products were isolated as a diastereomeric mixture and could not be separated because of their similar *R*_f_ values (Table 2).

#### (4′*R*,5′*R*)-1″,4′-Diphenyl-4′*H*-dispiro[fluorene-9,3′-pyrazole-5′,4″-pyrazolidine]-3″,5″-dione (5a) and (4′*S*,5′*R*)-1″,4′-diphenyl-4′*H*-dispiro[fluorene-9,3′-pyrazole-5′,4″-pyrazolidine]-3″,5″-dione (5′a)


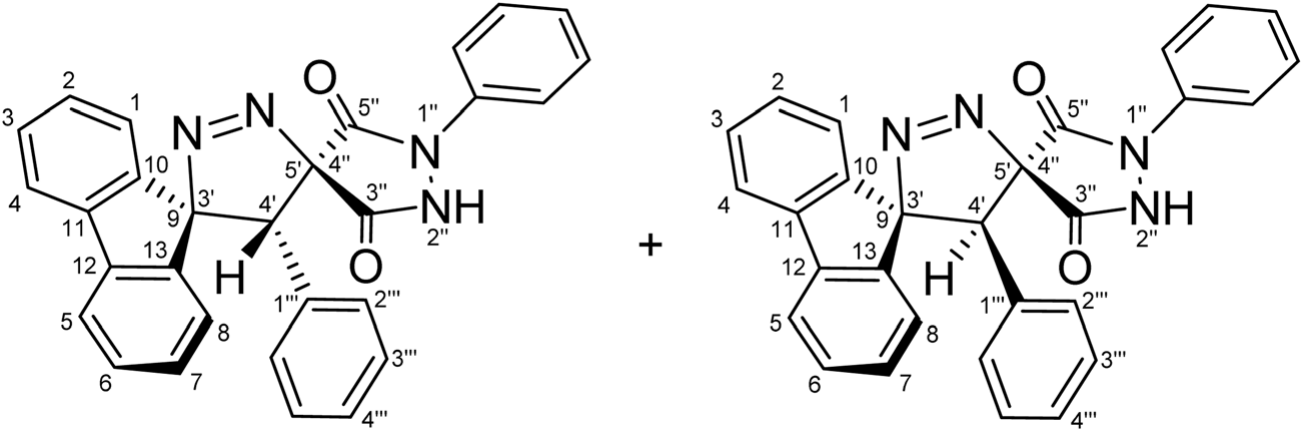
Isolated yield 78% as a diastereomeric mixture in a (52 : 48 ratio). ^1^H NMR (DMSO-d_6_, 600 MHz) *δ* 11.31 (1H, NH), 11.11 (1H, NH), 8.13 (d, *J* = 7.8 Hz, 1H), 8.11 (d, *J* = 7.8 Hz, 1H), 7.97–7.86 (m, 4H, Ar–H), 7.60–7.53 (m, 3H, Ar–H), 7.50–7.44 (m, 12H, Ar–H), 7.41–7.30 (m, 12H, Ar–H), 7.26–7.09 (m, 8H, Ar–H), 7.03–6.92 (m, 4H, Ar–H), 4.78 (s, 2H, C4′–H); ^13^C NMR (151 MHz, DMSO) *δ* 168.78 (C=O), 167.17 (C=O), 163.76 (C=O), 161.80 (C=O), 141.19 (q), 141.12 (q), 140.37 (q), 140.25 (q), 140.13 (q), 140.10 (q), 139.86 (q), 139.54 (q), 136.49 (q), 136.26 (q), 130.59 (CH), 129.67 (CH), 129.63 (q), 129.53 (CH), 129.01 (CH), 128.90 (CH), 128.83 (CH), 128.77 (CH), 128.54 (CH), 128.51 (CH), 128.48 (CH), 128.38 (CH), 128.14 (CH), 127.88 (CH), 127.85 (CH), 127.68 (CH), 126.32 (CH), 126.28 (CH), 125.91 (CH), 125.60 (CH), 125.13 (CH), 124.91 (CH), 124.76 (CH), 119.75 (CH), 119.74 (CH), 119.59 (CH), 119.57 (CH), 118.56 (CH), 118.52 (CH), 55.29 (C5′/C4″), 55.07 (C5′/C4″), 43.21 (C9/C3′), 42.99 (C9/C3′), 40.06 (C4′–H), 39.94 (C4′–H).

#### (4′*R*,5′*R*)-4′-(4-Methoxyphenyl)-1″-phenyl-4′*H*-dispiro[fluorene-9,3′-pyrazole-5′,4″-pyrazolidine]-3″,5″-dione (5b) and (4′*S*,5′*R*)-4′-(4-methoxyphenyl)-1″-phenyl-4′*H*-dispiro[fluorene-9,3′-pyrazole-5′,4″-pyrazolidine]-3″,5″-dione (5b)


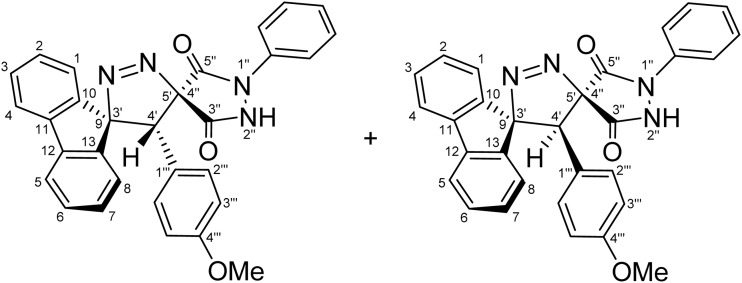
Isolated yield 65% as a diastereomeric mixture in a (50 : 50 ratio). ^1^H NMR (DMSO-d_6_, 600 MHz) *δ* 11.19 (2H, NH), 8.09 (d, *J* = 7.8 Hz, 1H), 8.08 (d, *J* = 7.8 Hz, 1H), 7.95–7.88 (m, 4H, Ar–H), 7.81–7.77 (m, 2H, Ar–H), 7.70–7.49 (m, 9H, Ar–H), 7.42–7.28 (m, 10H, Ar–H), 7.07–6.96 (m, 3H, Ar–H), 6.91–6.86 (m, 2H, Ar–H), 6.82–6.75 (m, 2H, Ar–H), 4.68 (s, 2H, C4′–H), 3.68 (s, 3H, OCH_3_), 3.67 (s, 3H, OCH_3_); ^13^C NMR (151 MHz, DMSO) *δ* 168.46 (C=O), 166.91 (C=O), 163.69 (C=O), 161.85 (C=O), 158.11 (C–O), 158.05 (C–O), 141.21 (q), 141.14 (q), 140.43 (q), 140.30 (q), 140.08 (q), 140.04 (q), 140.00 (q), 139.65 (q), 135.46 (CH), 133.31 (q), 131.78 (CH), 129.78 (CH), 129.64 (CH), 129.56 (CH), 128.97 (CH), 128.91 (CH), 128.57 (CH), 128.54 (CH), 128.48 (CH), 128.44 (CH), 128.40 (CH), 128.37 (CH), 128.17 (CH), 127.12 (CH), 126.90 (CH), 126.38 (CH), 126.33 (CH), 125.94 (CH), 125.62 (CH), 125.58 (CH), 125.44 (CH), 125.25 (CH), 124.96 (CH), 124.82 (CH), 124.02 (CH), 121.27 (CH), 120.64 (CH), 120.59 (CH), 119.79 (CH), 119.60 (CH), 118.61 (CH), 118.56 (CH), 116.40 (q), 116.15 (q), 113.88 (CH), 113.37 (CH), 113.33 (CH), 55.51 (C5′/C4″), 55.31 (C5′/C4″), 55.04 (OCH3), 55.01 (OCH3), 43.36 (C9/C3′), 43.12 (C9/C3′), 39.60 (C4′–H), 39.38 (C4′–H).

#### (4′*R*,5′*R*)-1″-Phenyl-4′-(*p*-tolyl)-4′*H*-dispiro[fluorene-9,3′-pyrazole-5′,4″-pyrazolidine]-3″,5″-dione (5c) and (4′*S*,5′*R*)-1″-phenyl-4′-(*p*-tolyl)-4′*H*-dispiro[fluorene-9,3′-pyrazole-5′,4″-pyrazolidine]-3″,5″-dione (5′c)


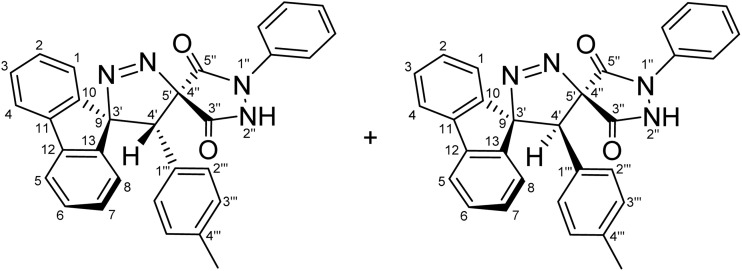
Isolated yield 79% as a diastereomeric mixture in a (61 : 39 ratio). ^1^H NMR (DMSO-d_6_, 600 MHz) *δ* 11.09 (2H, NH), 8.11 (d, *J* = 7.8 Hz, 1H), 8.09 (d, *J* = 7.8 Hz, 1H), 7.95–7.85 (m, 4H, Ar–H), 7.51 (d, *J* = 8.4 Hz, 2H), 7.50–7.30 (m, 13H, Ar–H), 7.18–6.97 (m, 13H, Ar–H), 4.71 (s, 2H, C4′–H), 2.33 (6H, CH_3_); ^13^C NMR (151 MHz, DMSO) *δ* 167.97 (C=O), 167.02 (C=O), 163.95 (C=O), 161.92 (C=O), 141.95 (q), 141.51 (q), 141.23 (q), 141.16 (q), 140.43 (q), 140.30 (q), 140.13 (q), 139.93 (q), 139.61 (q), 137.96 (q), 136.91 (q), 136.59 (q), 136.35 (q), 130.47 (CH), 129.78 (CH), 129.63 (CH), 129.58 (CH), 129.14 (CH), 128.98 (CH), 128.91 (CH), 128.60 (CH), 128.58 (CH), 128.55 (CH), 128.20 (CH), 126.56 (q), 126.46 (q), 126.38 (CH), 126.33 (CH), 125.96 (CH), 125.86 (CH), 125.64 (CH), 125.59 (CH), 125.44 (CH), 125.21 (CH), 124.99 (CH), 124.83 (CH), 119.80 (CH), 119.61 (CH), 118.63 (CH), 118.56 (CH), 55.43 (C5′/C4″), 55.22 (C5′/C4″), 43.27 (C9/C3′), 43.06 (C9/C3′), 39.91 (C4′–H), 39.68 (C4′–H), 20.89 (2xCH_3_).

#### (4′*R*,5′*R*)-4′-(4-Chlorophenyl)-1″-phenyl-4′*H*-dispiro[fluorene-9,3′-pyrazole-5′,4″-pyrazolidine]-3″,5″-dione (5d) and (4′*S*,5′*R*)-4′-(4-chlorophenyl)-1″-phenyl-4′*H*-dispiro[fluorene-9,3′-pyrazole-5′,4″-pyrazolidine]-3″,5″-dione (5′d)


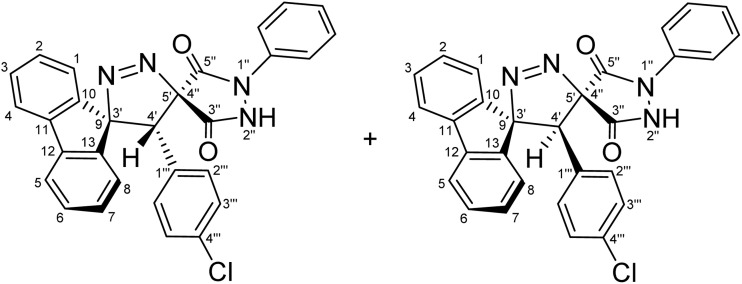
Isolated yield 82% as a diastereomeric mixture in a (62 : 38 ratio). ^1^H NMR (DMSO-d_6_, 600 MHz) *δ* 11.16 (2H, NH), 8.11 (d, *J* = 7.8 Hz, 1H), 8.09 (d, *J* = 7.8 Hz, 1H), 7.95–7.87 (m, 4H, Ar–H), 7.79–7.72 (m, 1H, Ar–H), 7.66–7.61 (m, 1H, Ar–H), 7.54 (d, *J* = 7.8 Hz, 2H), 7.49–7.43 (m, 4H, Ar–H), 7.42–7.31 (m, 10H, Ar–H), 7.27–7.20 (m, 4H, Ar–H), 7.19–7.08 (m, 2H, Ar–H), 7.08–7.00 (m, 2H, Ar–H), 6.97–6.93 (m, 2H, Ar–H), 4.74 (s, 2H, C4′–H); ^13^C NMR (151 MHz, DMSO) *δ* 168.46 (C=O), 166.91 (C=O), 163.69 (C=O), 161.85 (C=O), 141.30 (q), 141.23 (q), 140.40 (q), 140.28 (q), 139.72 (q), 139.41 (q), 138.27 (q), 136.42 (q), 136.19 (q), 135.83 (q), 135.73 (q), 132.67 (CH), 132.44 (q), 131.29 (q), 131.26 (q), 129.45 (CH), 129.32 (CH), 129.09 (CH), 129.02 (CH), 128.97 (CH), 128.91 (CH), 128.81 (q), 128.72 (q), 128.67 (CH), 128.64 (CH), 128.31 (CH), 127.93 (CH), 127.89 (CH), 126.41 (CH), 126.36 (CH), 126.04 (CH), 125.73 (CH), 125.37 (CH), 124.99 (CH), 124.87 (CH), 119.83 (CH), 119.76 (CH), 118.63 (CH), 118.59 (CH), 54.98 (C5′/C4″), 54.79 (C5′/C4″), 43.30 (C9/C3′), 43.11 (C9/C3′), 39.00 (C4′–H), 38.76 (C4′–H).

#### (4′*R*,5′*R*)-4′-(4-Fluorophenyl)-1″-phenyl-4′*H*-dispiro[fluorene-9,3′-pyrazole-5′,4″-pyrazolidine]-3″,5″-dione (5e) and (4′*S*,5′*R*)-4′-(4-fluorophenyl)-1″-phenyl-4′*H*-dispiro[fluorene-9,3′-pyrazole-5′,4″-pyrazolidine]-3″,5″-dione (5′e)


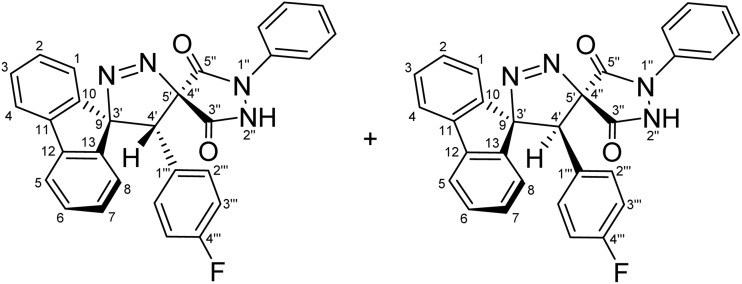
Isolated yield 59% as a diastereomeric mixture in a (55 : 45 ratio). ^1^H NMR (DMSO-d_6_, 600 MHz) *δ* 11.21 (2H, NH), 8.11 (d, *J* = 7.8 Hz, 1H), 8.09 (d, *J* = 7.8 Hz, 1H), 7.97–7.85 (m, 4H, Ar–H), 7.59–6.90 (m, 28H, Ar–H), 4.73 (s, 2H, C4′–H); ^13^C NMR (151 MHz, DMSO) *δ* 168.66 (C=O), 167.14 (C=O), 164.91 (d, *J* = 253.5 Hz, C–F) 163.67 (C=O), 161.80 (C=O), 161.62 (d, *J* = 243.0 Hz, C–F), 141.23 (q), 141.16 (q), 140.36 (q), 140.24 (q), 139.78 (q), 139.47 (q), 136.44 (q), 136.22 (q), 132.80 (d, *J* = 7.5 Hz, *meta* 2xCH_fluorophenyl_), 132.70 (d, *J* = 7.5 Hz, *meta* 2xCH_fluorophenyl_), 129.48 (CH), 129.35 (CH), 129.01 (CH), 128.88 (CH), 128.82 (CH), 128.55 (CH), 128.52 (CH), 128.18 (CH), 126.33 (CH), 126.28 (CH), 125.94 (CH), 125.84 (C_*q*-fluorophenyl_), 125.74 (d, *J* = 2.85 Hz, C_*q*-fluorophenyl_), 125.63 (CH), 125.26 (CH), 124.87 (CH), 124.75, 119.75 (CH), 119.66 (CH), 119.64 (CH), 118.54 (CH), 118.51 (CH), 114.70 (d, *J* = 21.3 Hz, *ortho* 2xCH_fluorophenyl_), 114.67 (d, *J* = 21.3 Hz, *ortho* 2xCH_fluorophenyl_), 55.09 (C5′/C4″), 54.89 (C5′/C4″), 43.32 (C9/C3′), 43.11 (C9/C3′) 38.96 (C4′–H), 38.76 (C4′–H).

#### (4′*R*,5′*R*)-4′-(4-Cyanophenyl)-1″-phenyl-4′*H*-dispiro[fluorene-9,3′-pyrazole-5′,4″-pyrazolidine]-3″,5″-dione (5f) and (4′*S*,5′*R*)-4′-(4-cyanophenyl)-1″-phenyl-4′*H*-dispiro[fluorene-9,3′-pyrazole-5′,4″-pyrazolidine]-3″,5″-dione (5′f)


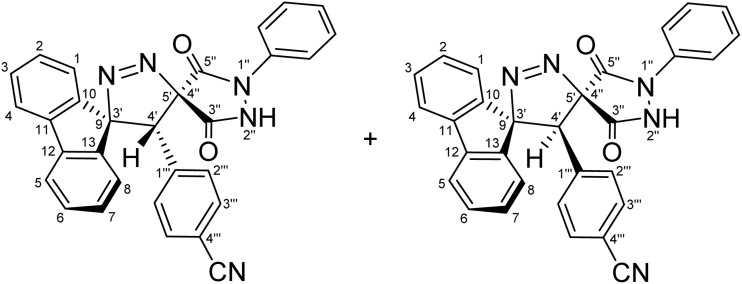
Isolated yield 91% as a diastereomeric mixture in a (51 : 49 ratio). ^1^H NMR (DMSO-d_6_, 600 MHz) *δ* 11.31 (1H, NH), 11.17 (1H, NH), 8.68 (d, *J* = 8.4 Hz, 1H), 8.15–7.73 (m, 11H, Ar–H), 7.59–6.82 (m, 22H, Ar–H), 4.85 (s, 2H, C4′–H); ^13^C NMR (DMSO-d_6_, 151 MHz) *δ* 166.68 (C=O), 165.32 (C=O), 161.69 (C=O), 159.98 (C=O), 139.61 (C1‴, q), 139.52 (q), 138.66 (q), 138.53 (q), 137.82 (C1, q), 137.53 (C1, q), 134.58 (q), 134.33 (q), 134.01 (q), 133.93 (q), 132.21 (CH), 130.71 (CH), 130.63 (CH), 130.28 (CH), 129.98 (CH), 129.95 (C(2)–H), 127.47 (C(2)–H), 127.36 (CH), 127.34 (CH), 127.24 (CH), 127.21 (CH), 127.15 (CH), 126.97 (CH), 126.95 (CH), 126.62 (CH), 124.67 (CH), 124.63 (CH), 124.37 (CH), 124.07 (CH), 123.70 (CH), 123.24 (CH), 123.14 (CH), 118.11 (CH), 117.11 (2C, q), 116.86 (CH), 116.75 (NC–C), 116.43 (NC–C), 108.78 (CN), 108.77 (CN), 52.91 (C5′/C4″), 52.72 (C5′/C4″), 41.60 (C9/C3′), 41.42 (C9/C3′), 37.27 (C4′–H), 37.08 (C4′–H).

#### (4′*R*,5′*R*)-4′-(4-Nitrophenyl)-1″-phenyl-4′*H*-dispiro[fluorene-9,3′-pyrazole-5′,4″-pyrazolidine]-3″,5″-dione (5g) and (4′*S*,5′*R*)-4′-(4-nitrophenyl)-1″-phenyl-4′*H*-dispiro[fluorene-9,3′-pyrazole-5′,4″-pyrazolidine]-3″,5″-dione (5′g)


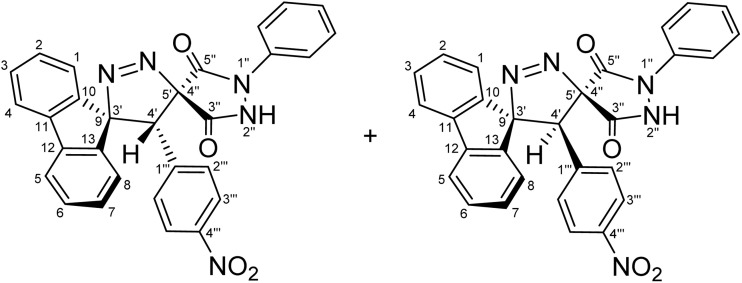
Isolated yield 89% as a diastereomeric mixture in a (51 : 49 ratio). ^1^H NMR (DMSO-d_6_, 600 MHz) *δ* 11.32 (1H, NH), 11.20 (1H, NH), 8.21 (d, *J* = 9.0 Hz, 2H, *ortho* 2xCH_nitrophenyl_), 8.20 (d, *J* = 9.0 Hz, 2H, *ortho* 2xCH_nitrophenyl_), 8.13 (d, *J* = 7.8 Hz, 1H), 8.10 (d, *J* = 7.8 Hz, 1H), 7.98–7.90 (m, 4H, Ar–H), 7.60–7.52 (m, 6H, Ar–H), 7.51–7.44 (m, 3H, Ar–H), 7.42–7.30 (m, 9H, Ar–H), 7.16 (t, *J* = 7.2 Hz, 1H), 7.12 (t, *J* = 7.8 Hz, 1H), 7.04 (t, *J* = 7.8 Hz, 1H), 7.01 (t, *J* = 7.8 Hz, 1H), 6.90–6.86 (m, 2H, Ar–H), 4.89 (s, 2H, C4′–H); ^13^C NMR (151 MHz, DMSO) *δ* 168.34 (C=O), 166.97 (C=O), 163.30 (C=O), 161.63 (C=O), 146.96 (2xC–NO_2_), 141.31 (q), 141.23 (q), 140.35 (q), 140.22 (q), 139.43 (q), 139.14 (q), 137.84 (C_*q*-nitrophenyl_), 137.77 (C_*q*-nitrophenyl_), 136.19 (q), 135.97 (q), 132.31 (*meta* 2xCH_nitrophenyl_), 132.30 (*meta* 2xCH_nitrophenyl_), 129.12 (CH), 128.98 (CH), 128.90 (CH), 128.84 (CH), 128.69 (CH), 128.66 (CH), 128.35 (CH), 128.34 (CH), 126.36 (CH), 126.32 (CH), 126.10 (CH), 125.80 (CH), 125.44 (CH), 124.92 (CH), 124.83 (CH), 122.83 (*ortho* 2xCH_nitrophenyl_), 122.80 (*ortho* 2xCH_nitrophenyl_), 119.85 (CH), 119.82 (CH), 119.81 (CH), 118.52 (CH), 118.11 (q), 54.56 (C5′/C4″), 54.38 (C5′/C4″), 43.35 (C9/C3′), 43.19 (C9/C3′), 38.64 (C4′–H), 38.46 (C4′–H).

## Author contributions

Essam M. Hussein: methodology, investigation, conceptualization, formal analysis, validation, data curation, software, visualization, resources, writing – original and final draft; Ziad Moussa: investigation, conceptualization, formal analysis, data curation, software, visualization, funding acquisition, original and final draft; Munirah M. Al-Rooqi: methodology, investigation, conceptualization, funding acquisition, resources, writing – original draft; Saeed S. Samman: investigation, conceptualization, methodology, conceptualization, data curation, funding acquisition; Abdulrahman A. Alsimaree: investigation, conceptualization, methodology, conceptualization, data curation, funding acquisition; Rabab S. Jassas: data curation, funding acquisition, writing – original and final draft; Saleh A. Ahmed: methodology, investigation, data curation, formal analysis, conceptualization, funding acquisition, supervision, project administration, resources, writing – original and final draft.

## Conflicts of interest

The authors declare that they have no competing interests.

## Funding

This research work was funded by Umm Al-Qura University, Saudi Arabia under grant number: 26UQU4281605GSSR01.

## Supplementary Material

RA-016-D6RA01559J-s001

## Data Availability

The data underlying this study are available in the published article and in the supporting information (SI). All experimental spectra and relevant computational input/output files are provided in the SI in accordance with RSC data requirements. Supplementary information is available. See DOI: https://doi.org/10.1039/d6ra01559j.
