# Supplementary material for: Innovative regioselective synthesis of dispiro[fluorene-9,3′-pyrazole-5′,4″-pyrazolidines]: experimental and computational study
Source: RSC Adv. 2026 May 11;16(25):22735–62. doi: 10.1039/d6ra01559j (PMC13159531; doi:10.1039/d6ra01559j)

Supplementary Information (SI) for RSC Advances.
This journal is © The Royal Society of Chemistry 2026

SUPPORTING INFORMATION FOR

Innovative Regioselective Synthesis of Dispiro[fluorene-9,3'-pyrazole-5',4''-pyrazolidines]: Experimental and Computational Study

Essam M. Hussein[a,*], Ziad Moussa[b,*], Munirah M. Al-Rooqi[c], Saeed S. Samman[d], Abdulrahman A. Alsimaree[e], Rabab S. Jassas[f], Saleh A. Ahmed[c,*]

[a]*Chemistry Chemistry, Faculty of Science, Assiut University, 71516 Assiut, Egypt*

[b]*Department of Chemistry, College of Science, United Arab Emirates University, P.O. Box 15551, Al Ain, United Arab Emirates*

[c]*Department of Chemistry, Faculty of Science, Umm Al-Qura University, 21955 Makkah, Saudi Arabia*

[d]*Deparment of Basic Science and Technologies, Applied College, Taibah University, 42353 Madina, Saudi Arabia*

[e]*Department of Chemistry, College of Science and Humanities, Shaqra University, Shaqra, Saudi Arabia*

[f]*Department of Chemistry, Jamoum University College, Umm Al-Qura University, 21955 Makkah, Saudi Arabia*

Correspondence may be addressed to:

Essam M. Hussein, https://orcid.org/0000-0003-4778-540X; E-mail: amhfarghaly@uqu.edu.sa, essam.hussein78@yahoo.com; Ziad Mousaa, https://orcid.org/0000-0002-3365-0451 ; E-mail: zmoussa@uaeu.ac.ae; Saleh A. Ahmed, https://orcid.org/0000-0002-2364-0380; E-mail: saahmed@uqu.edu.sa, saleh_63@hotmail.com

Table of Contents: **page**

(E)/(Z)-4-(4-benzylidene)-1-phenylpyrazolidine-3,5-dione (3a)	S3
(E)/(Z)-4-(4-methoxybenzylidene)-1-phenylpyrazolidine-3,5-dione (3b)	S6
(E)/(Z)-4-(4-methylbenzylidene)-1-phenylpyrazolidine-3,5-dione (3c)	S9
(E)/(Z)-4-(4-chlorobenzylidene)-1-phenylpyrazolidine-3,5-dione (3d)	S12
(E)/(Z)-4-(4-fluorobenzylidene)-1-phenylpyrazolidine-3,5-dione (3e)	S15
(E)/(Z)-4-(4-cyanobenzylidene)-1-phenylpyrazolidine-3,5-dione (3f)	S19
(E)/(Z)-4-(4-nitrobenzylidene)-1-phenylpyrazolidine-3,5-dione (3g)	S22
(4'R,5'R)-1'',4'-diphenyl-4'H-dispiro[fluorene-9,3'-pyrazole-5',4''-pyrazolidine]-3'',5''-dione (5a) and (4'S,5'R)-1'',4'-diphenyl-4'H-dispiro[fluorene-9,3'-pyrazole-5',4''-pyrazolidine]-3'',5''-dione (5'a)	S25
(4'R,5'R)-1''-phenyl-4'-(p-tolyl)-4'H-dispiro[fluorene-9,3'-pyrazole-5',4''-pyrazolidine]-3'',5''-dione (5c) and (4'S,5'R)-1''-phenyl-4'-(p-tolyl)-4'H-dispiro[fluorene-9,3'-pyrazole-5',4''-pyrazolidine]-3'',5''-dione (5'c)	S31
(4'R,5'R)-1''-phenyl-4'-(p-tolyl)-4'H-dispiro[fluorene-9,3'-pyrazole-5',4''-pyrazolidine]-3'',5''-dione (5c) and (4'S,5'R)-1''-phenyl-4'-(p-tolyl)-4'H-dispiro[fluorene-9,3'-pyrazole-5',4''-pyrazolidine]-3'',5''-dione (5'c)	S33
((4'R,5'R)-4'-(4-chlorophenyl)-1''-phenyl-4'H-dispiro[fluorene-9,3'-pyrazole-5',4''-pyrazolidine]-3'',5''-dione (5d) and (4'S,5'R)-4'-(4-chlorophenyl)-1''-phenyl-4'H-dispiro[fluorene-9,3'-pyrazole-5',4''-pyrazolidine]-3'',5''-dione (5'd)	S39
(4'R,5'R)-4'-(4-fluorophenyl)-1''-phenyl-4'H-dispiro[fluorene-9,3'-pyrazole-5',4''-pyrazolidine]-3'',5''-dione (5e) and (4'S,5'R)-4'-(4-fluorophenyl)-1''-phenyl-4'H-dispiro[fluorene-9,3'-pyrazole-5',4''-pyrazolidine]-3'',5''-dione (5'e)	S46
(4'R,5'R)-4'-(4-cyanophenyl)-1''-phenyl-4'H-dispiro[fluorene-9,3'-pyrazole-5',4''-pyrazolidine]-3'',5''-dione (5f) and (4'S,5'R)-4'-(4-cyanophenyl)-1''-phenyl-4'H-dispiro[fluorene-9,3'-pyrazole-5',4''-pyrazolidine]-3'',5''-dione (5'f)	S52
(4'R,5'R)-4'-(4-nitrophenyl)-1''-phenyl-4'H-dispiro[fluorene-9,3'-pyrazole-5',4''-pyrazolidine]-3'',5''-dione (5g) and (4'S,5'R)-4'-(4-nitrophenyl)-1''-phenyl-4'H-dispiro[fluorene-9,3'-pyrazole-5',4''-pyrazolidine]-3'',5''-dione (5'g)	S58

^{1}H NMR spectrum of (E)/(Z)-4-(4-benzylidene)-1-phenylpyrazolidine-3,5-dione (3a)

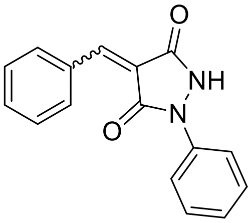

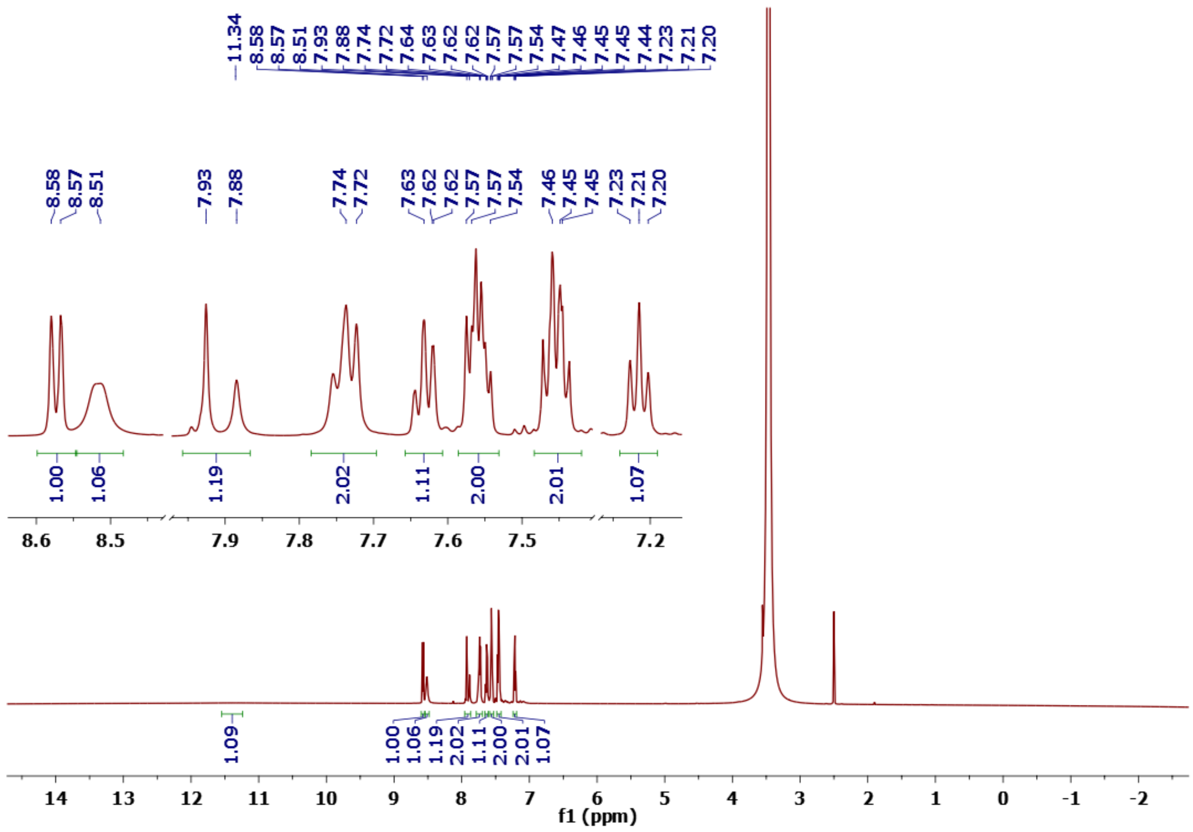

^{13}C NMR spectrum of (E)/(Z)-4-(4-benzylidene)-1-phenylpyrazolidine-3,5-dione (3a)

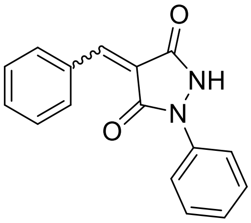

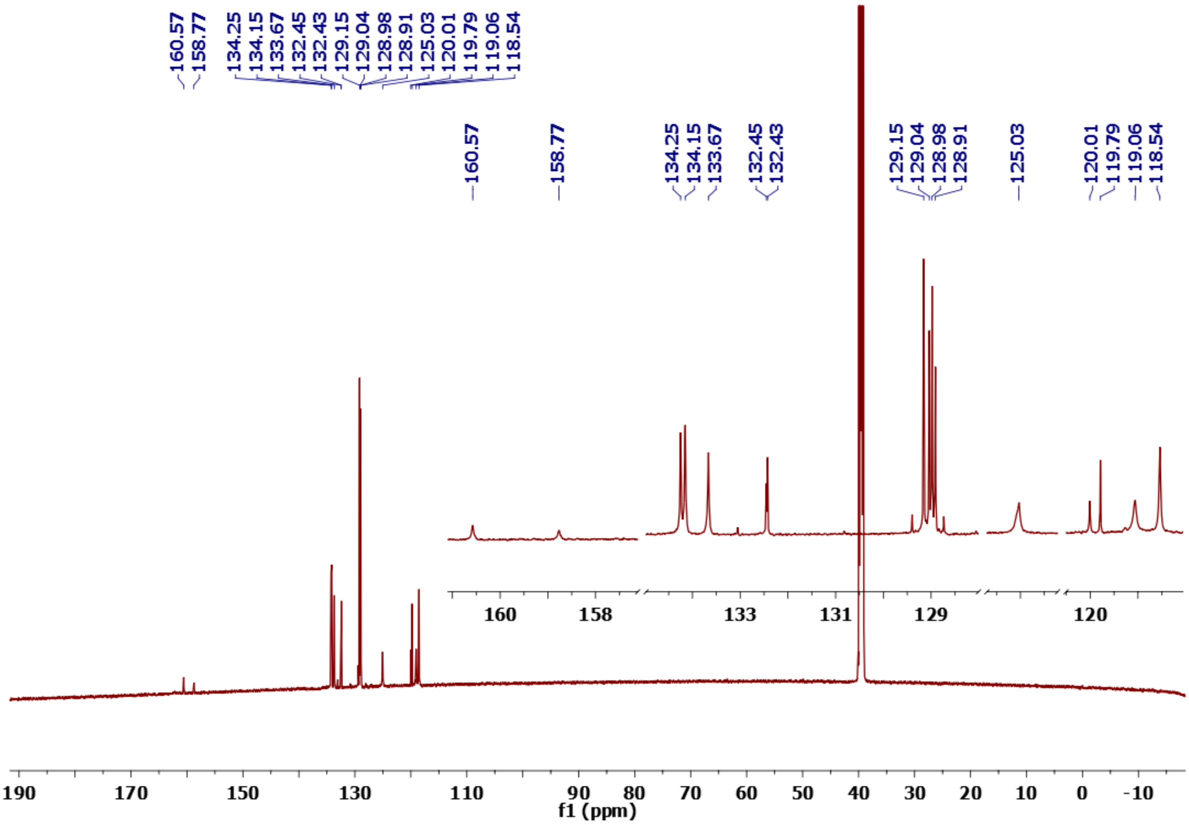

^{13}C DEPT-135 NMR spectrum of (E)/(Z)-4-(4-benzylidene)-1-phenylpyrazolidine-3,5-dione (3a)

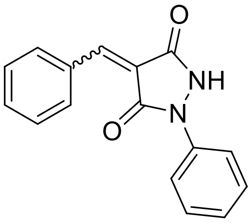

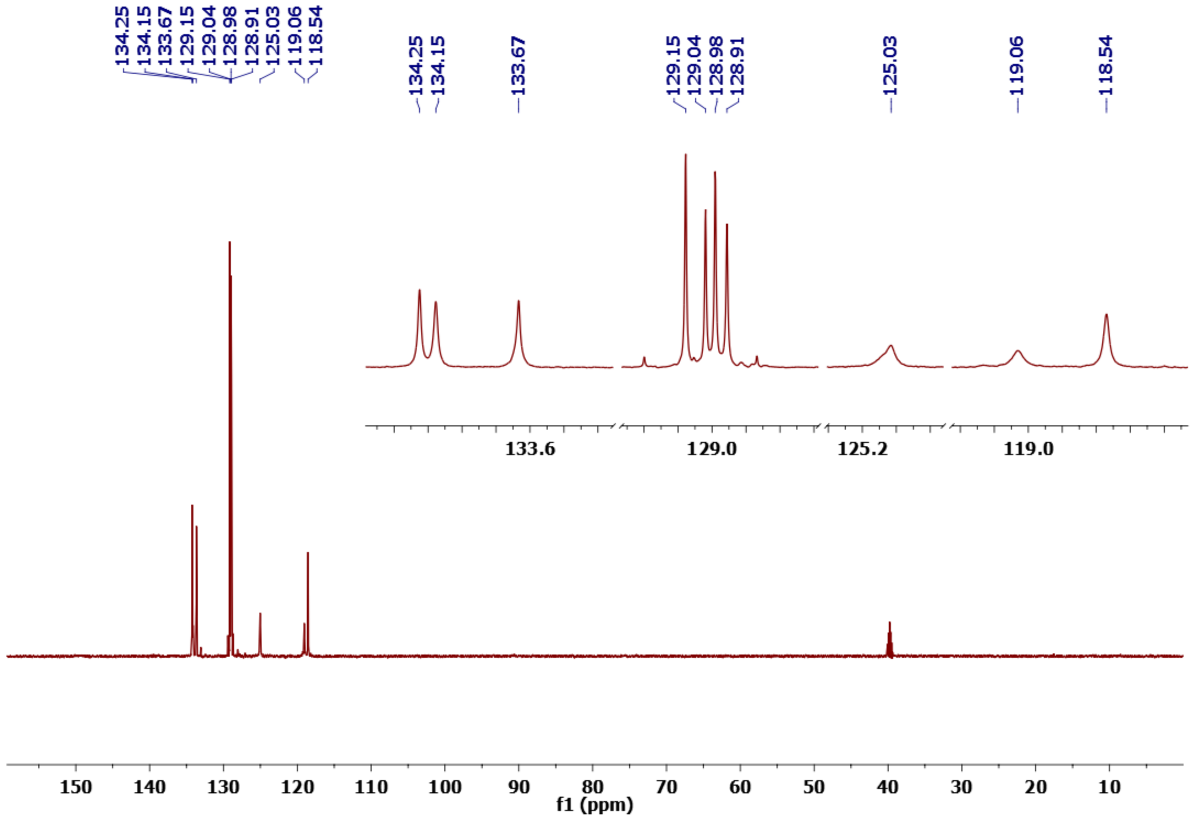

^{1}H NMR spectrum of (E)/(Z)-4-(4-methoxybenzylidene)-1-phenylpyrazolidine-3,5-dione (3b)

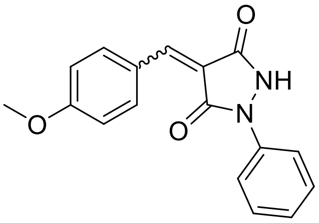

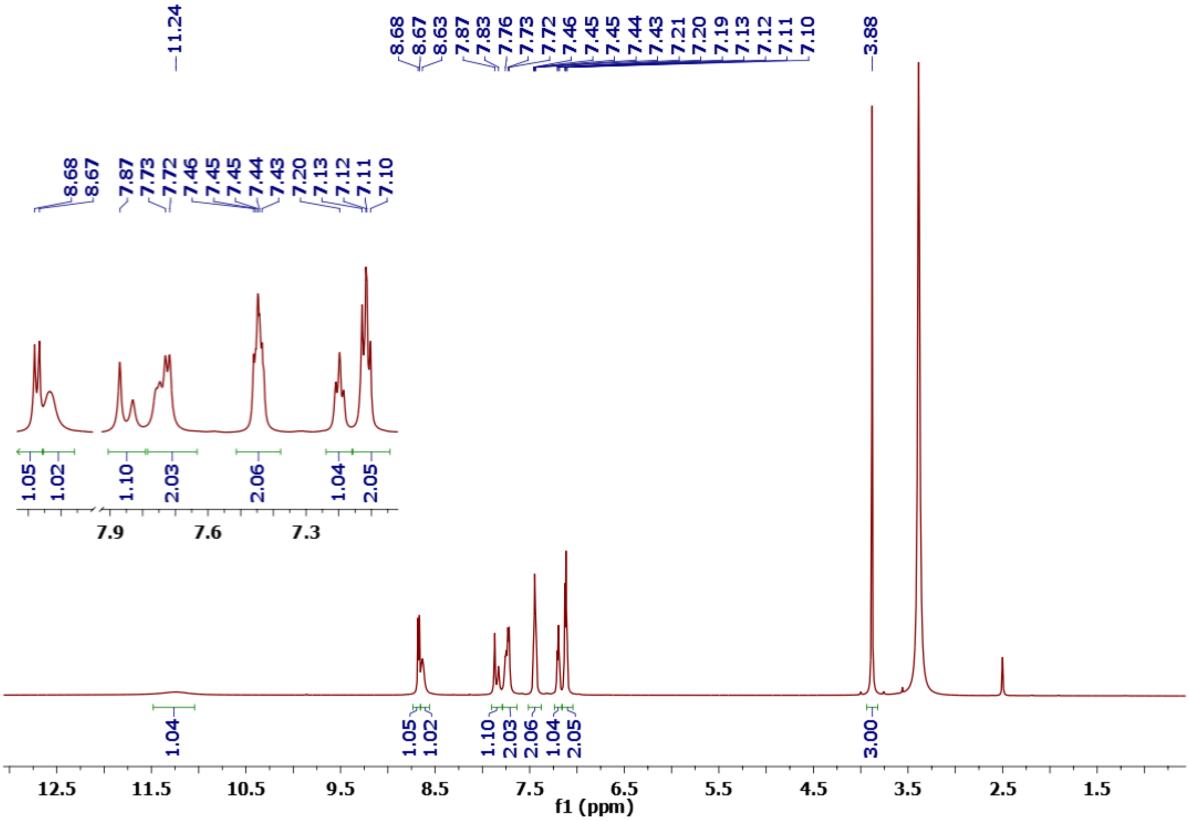

^{13}C NMR spectrum of (E)/(Z)-4-(4-methoxybenzylidene)-1-phenylpyrazolidine-3,5-dione (3b)

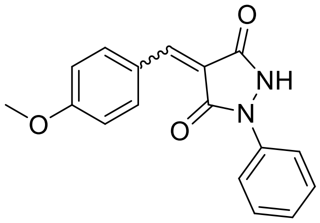

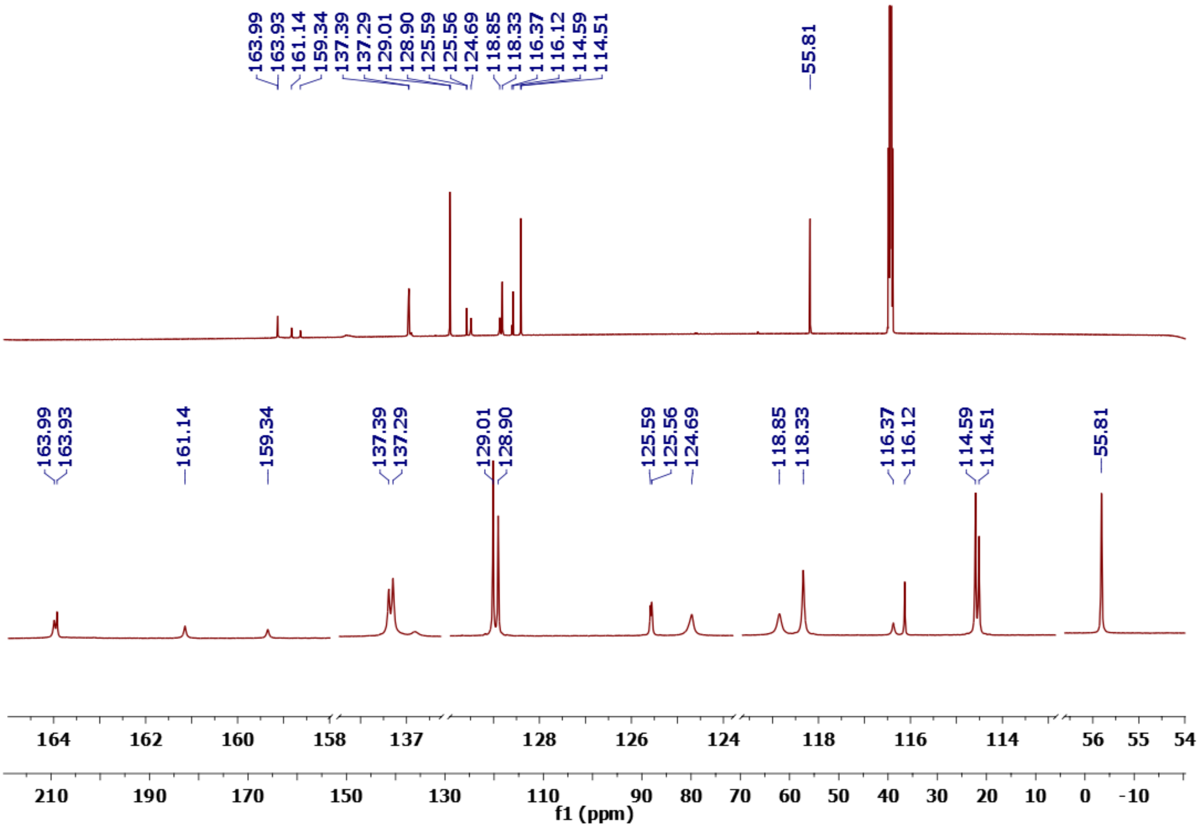

^{13}C DEPT-135 NMR spectrum of (E)/(Z)-4-(4-methoxybenzylidene)-1-phenylpyrazolidine-3,5-dione (3b)

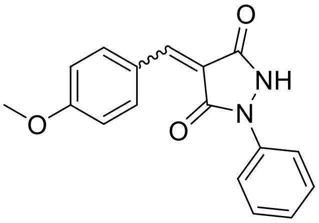

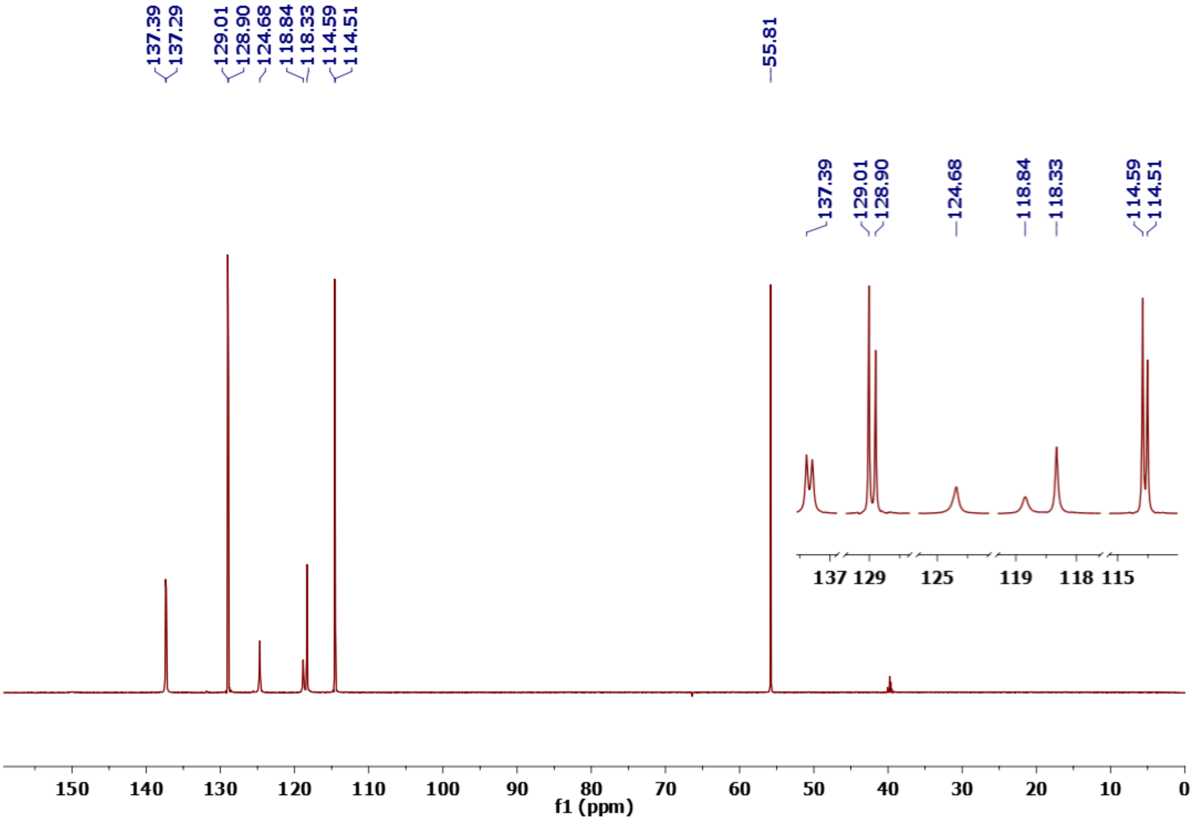

^{1}H NMR spectrum of (E)/(Z)-4-(4-methylbenzylidene)-1-phenylpyrazolidine-3,5-dione (3c)

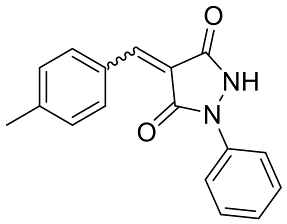

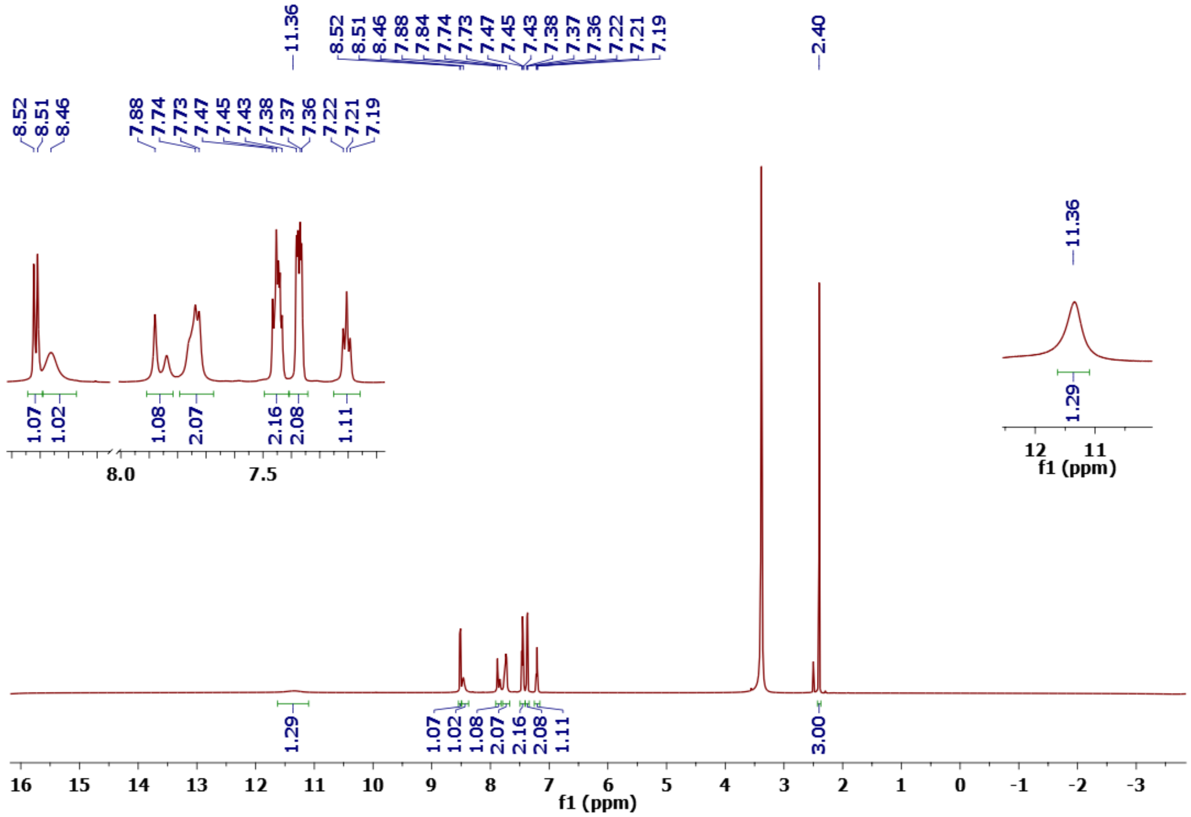

^{13}C NMR spectrum of (E)/(Z)-4-(4-methylbenzylidene)-1-phenylpyrazolidine-3,5-dione (3c)

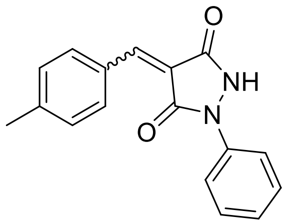

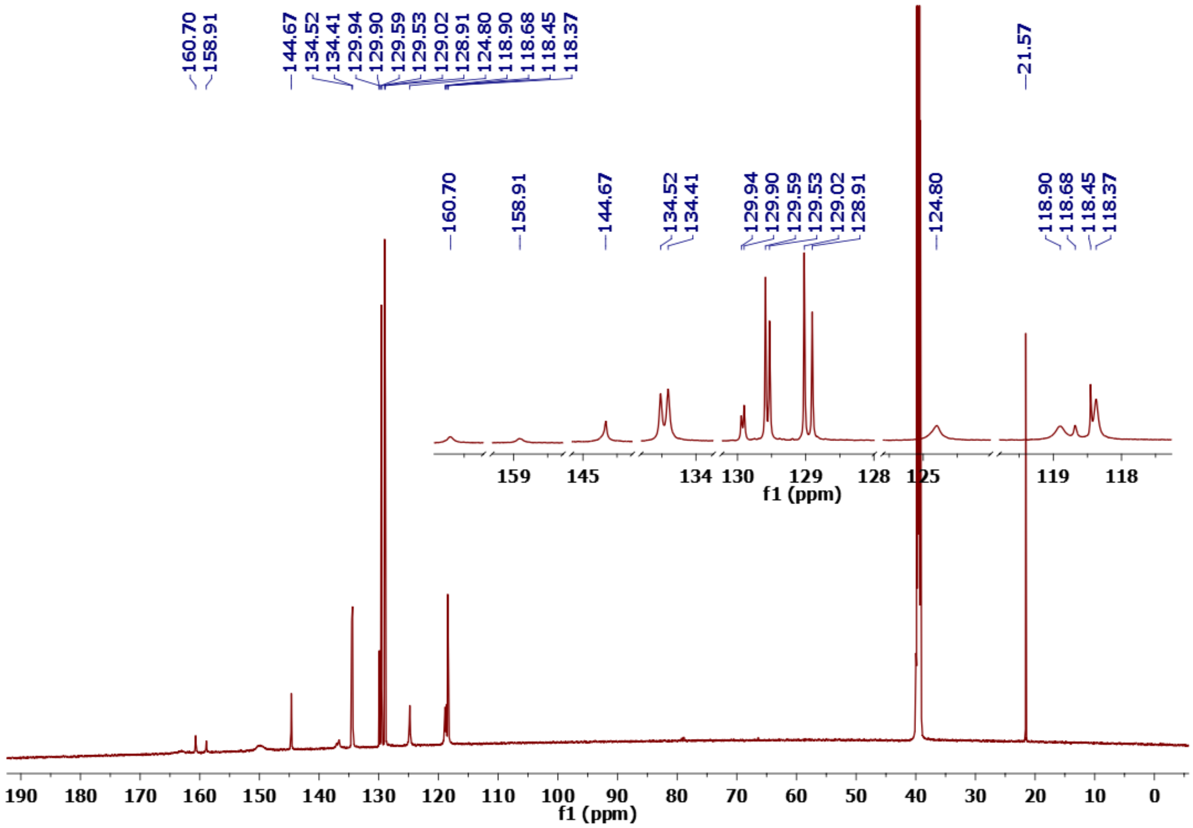

^{13}C DEPT-135 NMR spectrum of (E)/(Z)-4-(4-methylbenzylidene)-1-phenylpyrazolidine-3,5-dione (3c)

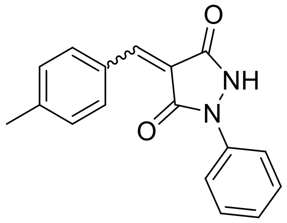

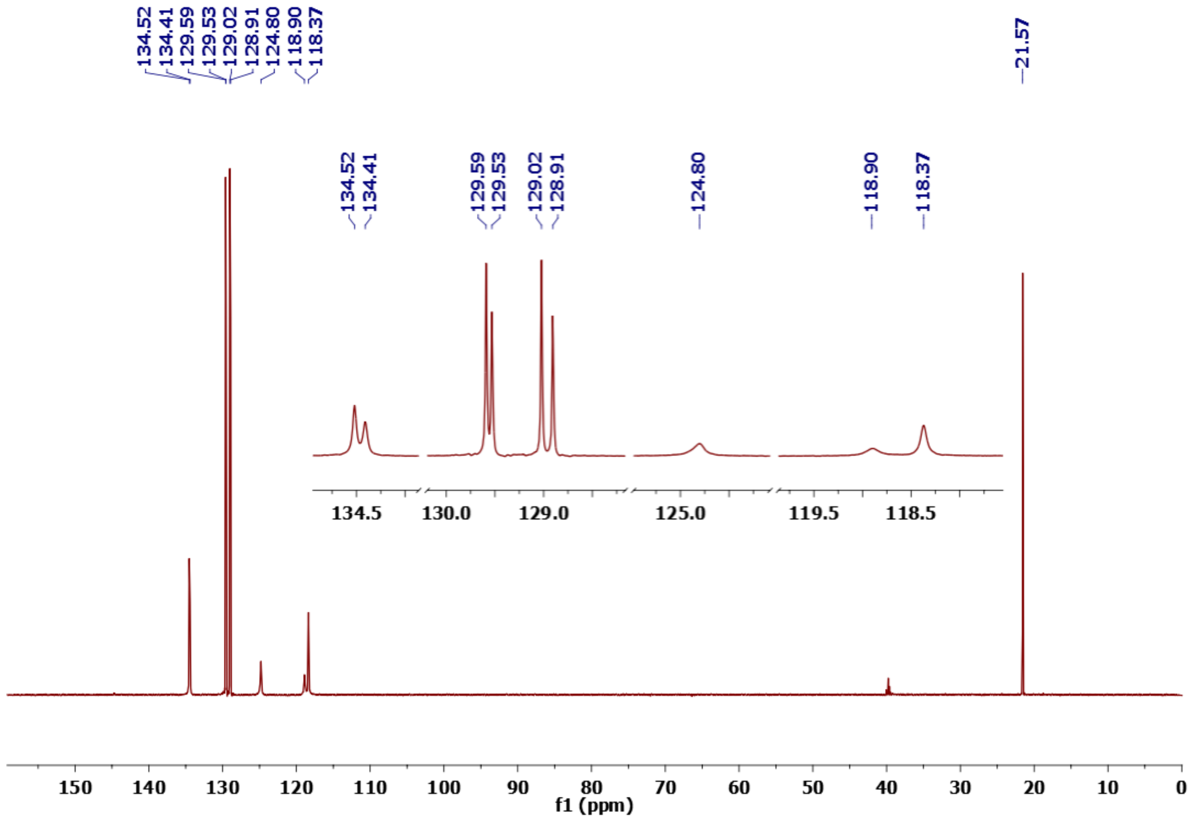

¹H NMR spectrum of (E)/(Z)-4-(4-chlorobenzylidene)-1-phenylpyrazolidine-3,5-dione (3d)

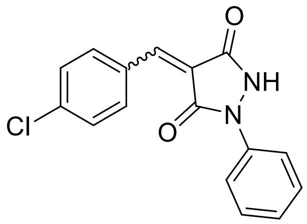

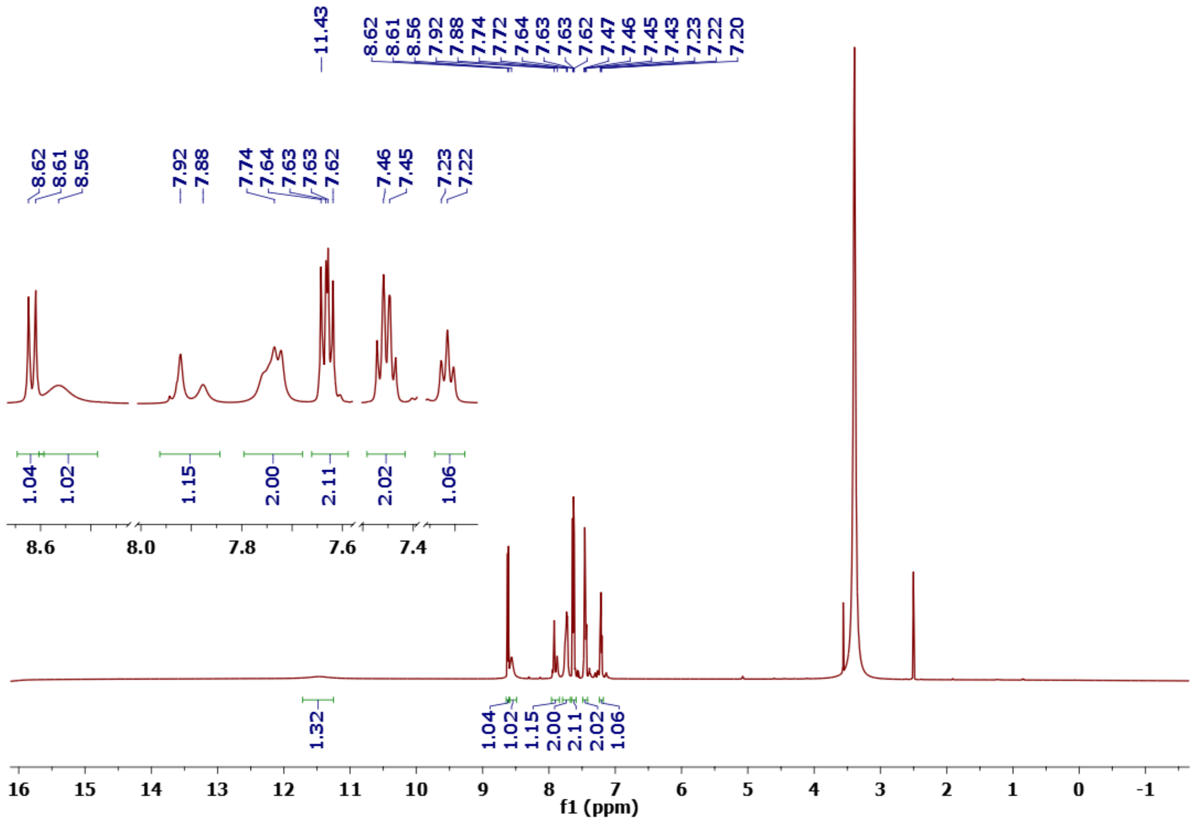

^{13}C NMR spectrum of (E)/(Z)-4-(4-chlorobenzylidene)-1-phenylpyrazolidine-3,5-dione (3d)

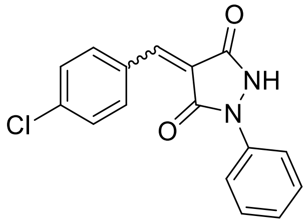

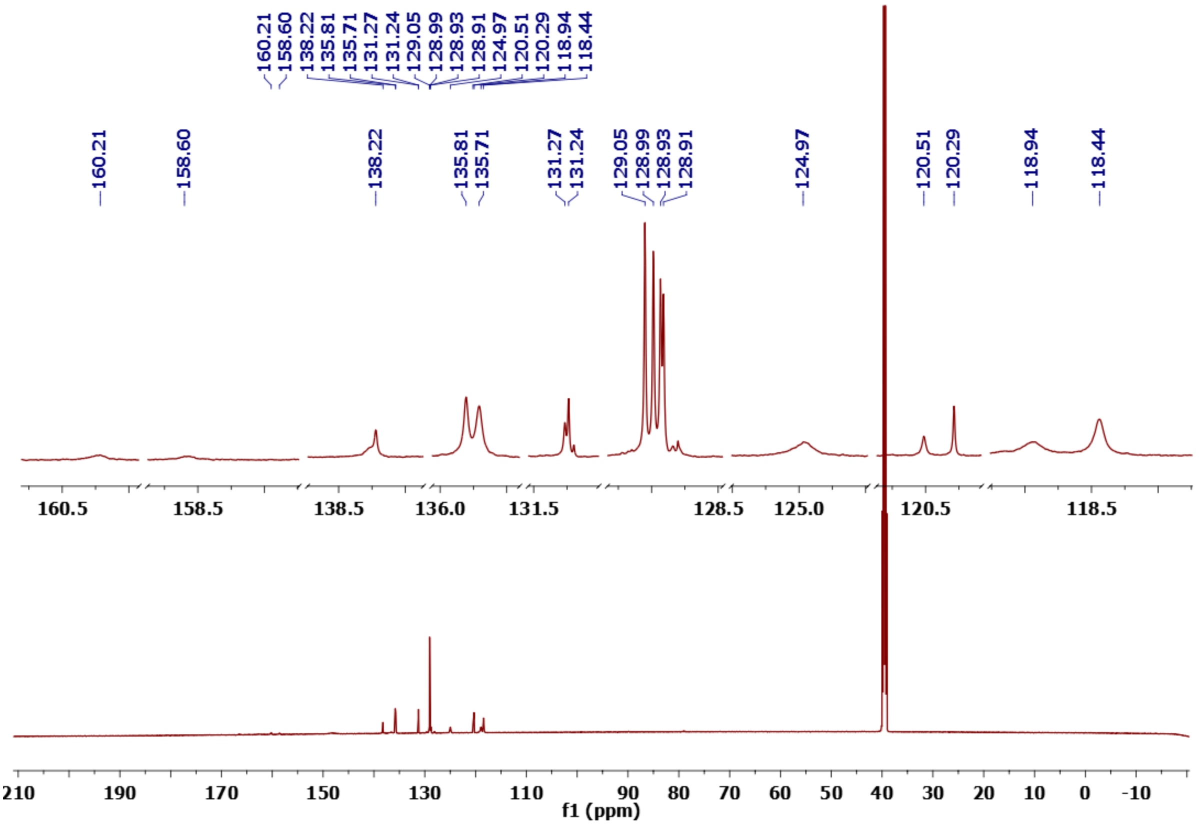

^{13}C DEPT-135 NMR spectrum of (E)/(Z)-4-(4-chlorobenzylidene)-1-phenylpyrazolidine-3,5-dione (3d)

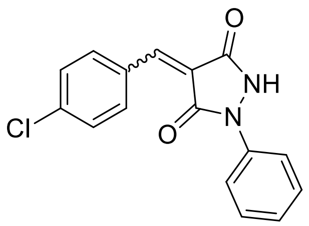

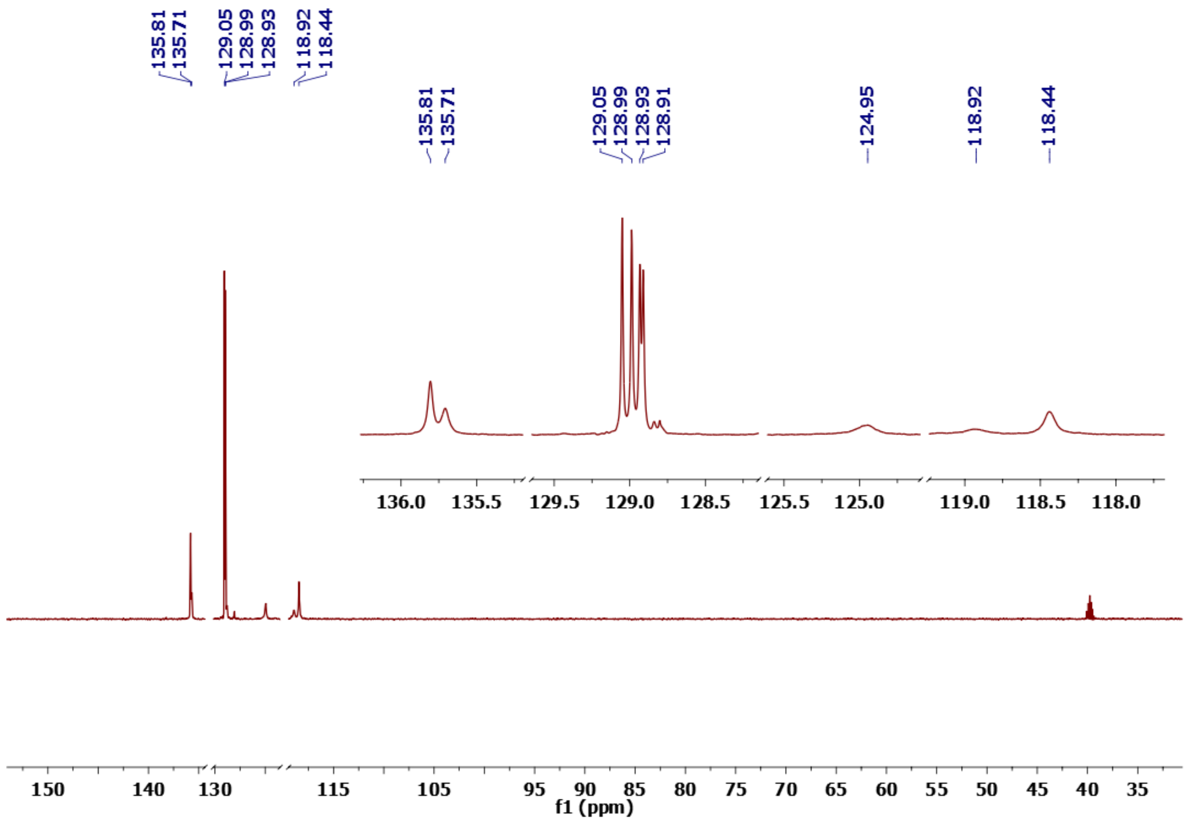

^{1}H NMR spectrum of (E)/(Z)-4-(4-fluorobenzylidene)-1-phenylpyrazolidine-3,5-dione (3e)

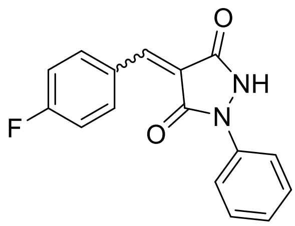

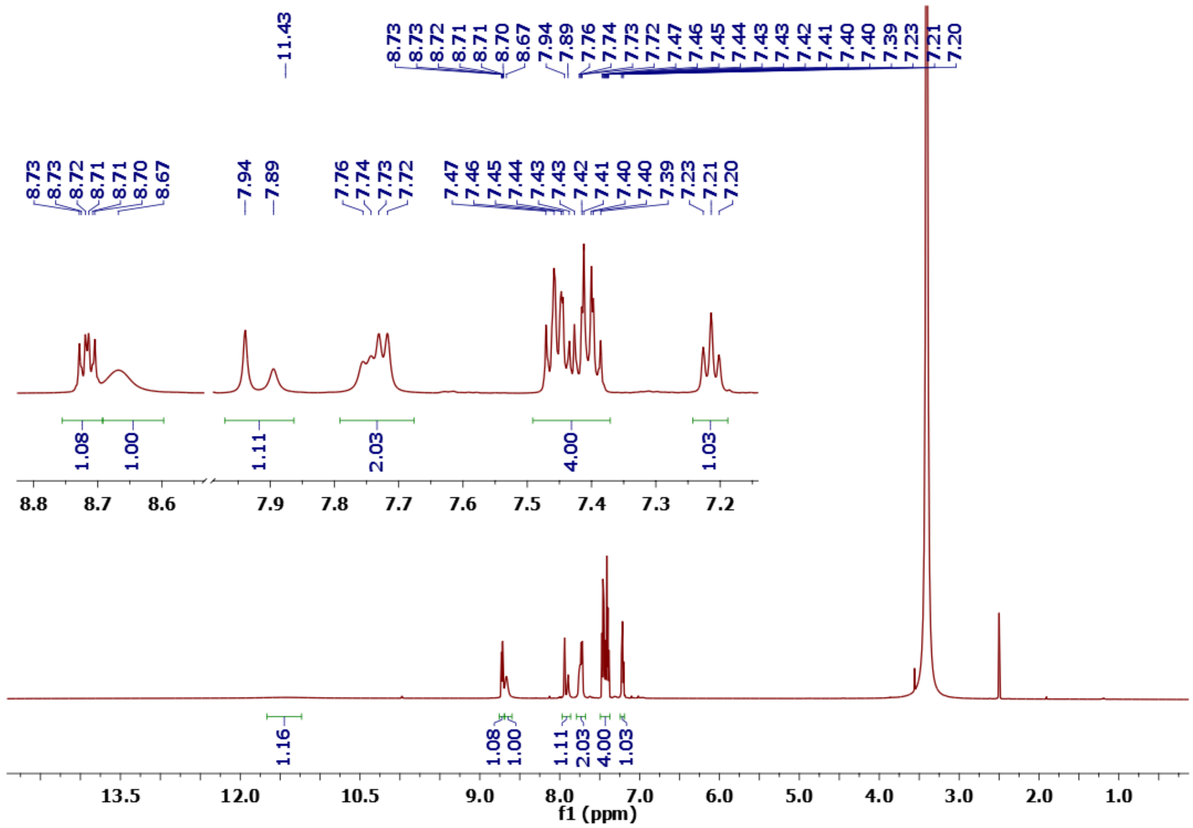

^{13}C NMR spectrum of (E)/(Z)-4-(4-fluorobenzylidene)-1-phenylpyrazolidine-3,5-dione (3e)

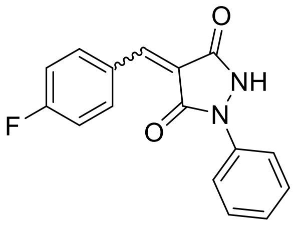

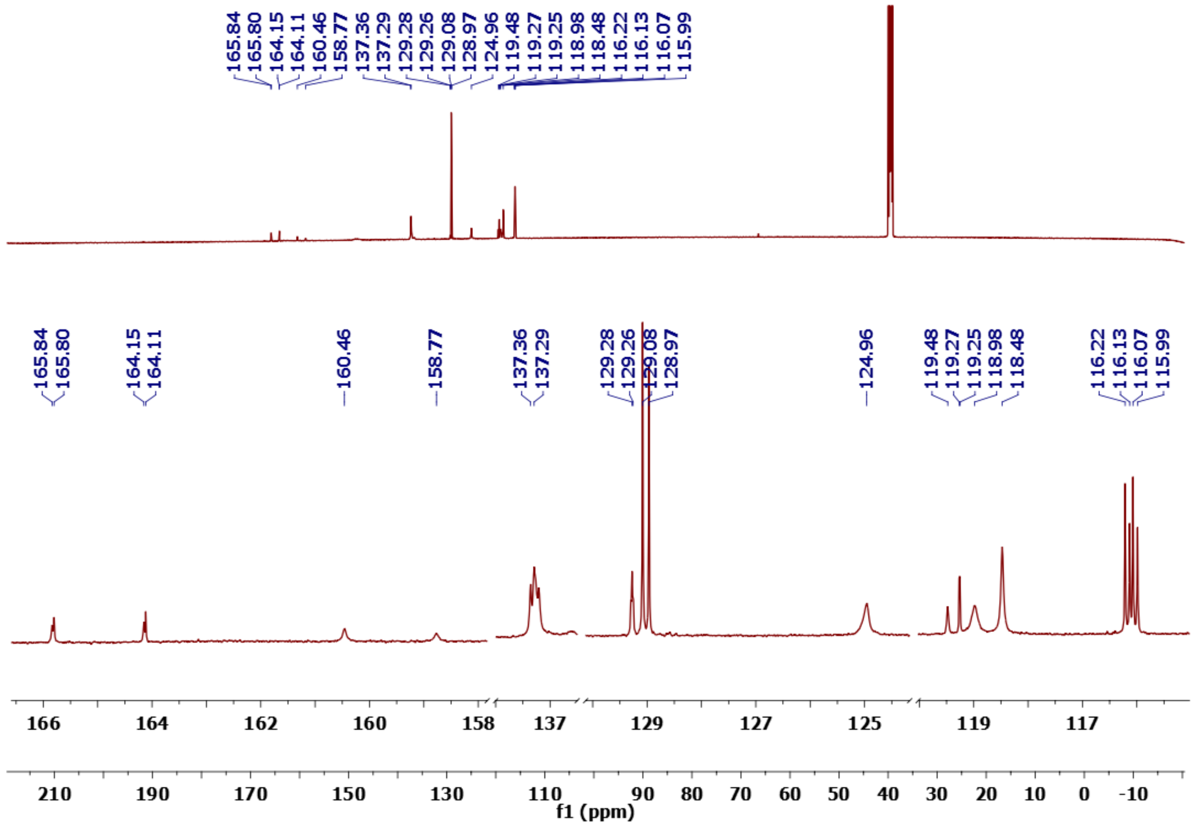

^{13}C DEPT-135 NMR spectrum of (E)/(Z)-4-(4-fluorobenzylidene)-1-phenylpyrazolidine-3,5-dione (3e)

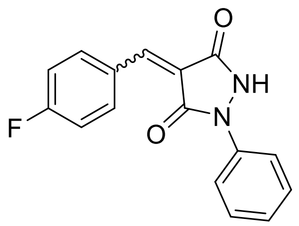

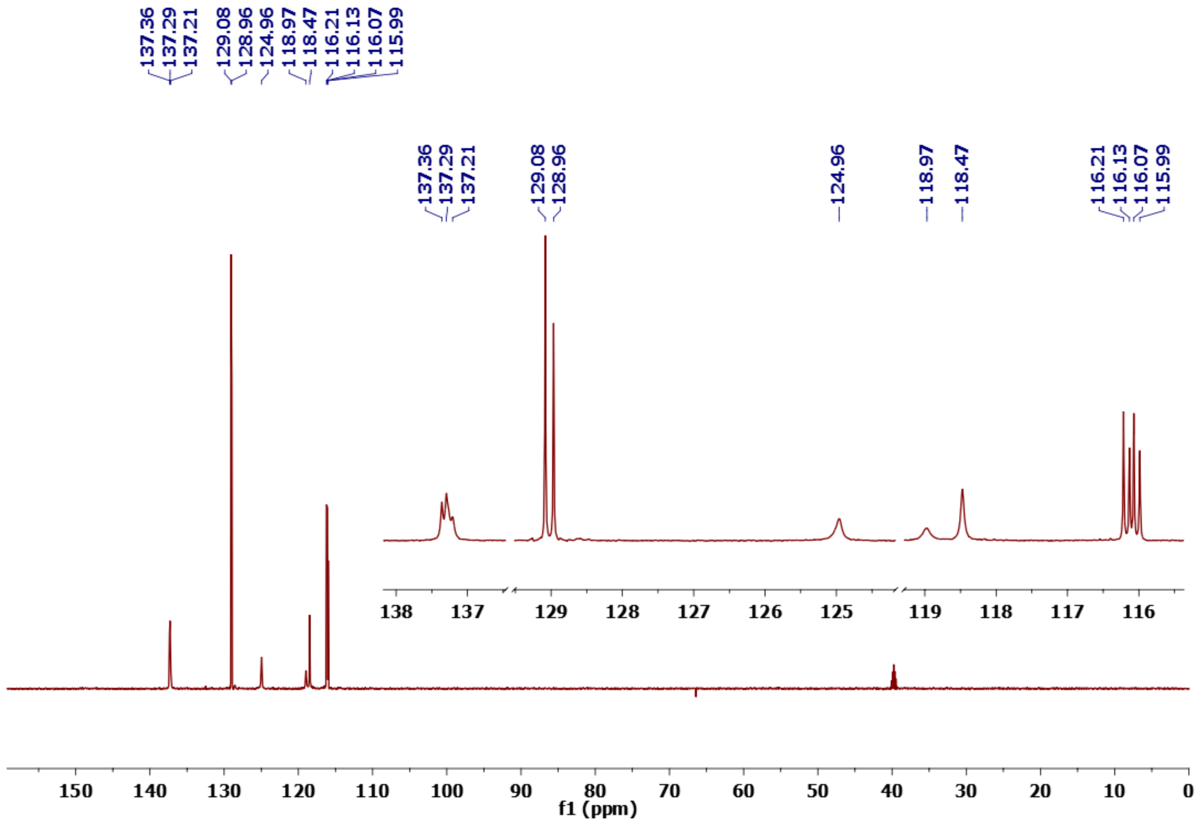

^{19}F NMR spectrum of (E)/(Z)-4-(4-fluorobenzylidene)-1-phenylpyrazolidine-3,5-dione (3e)

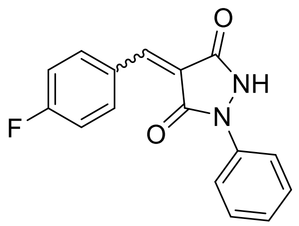

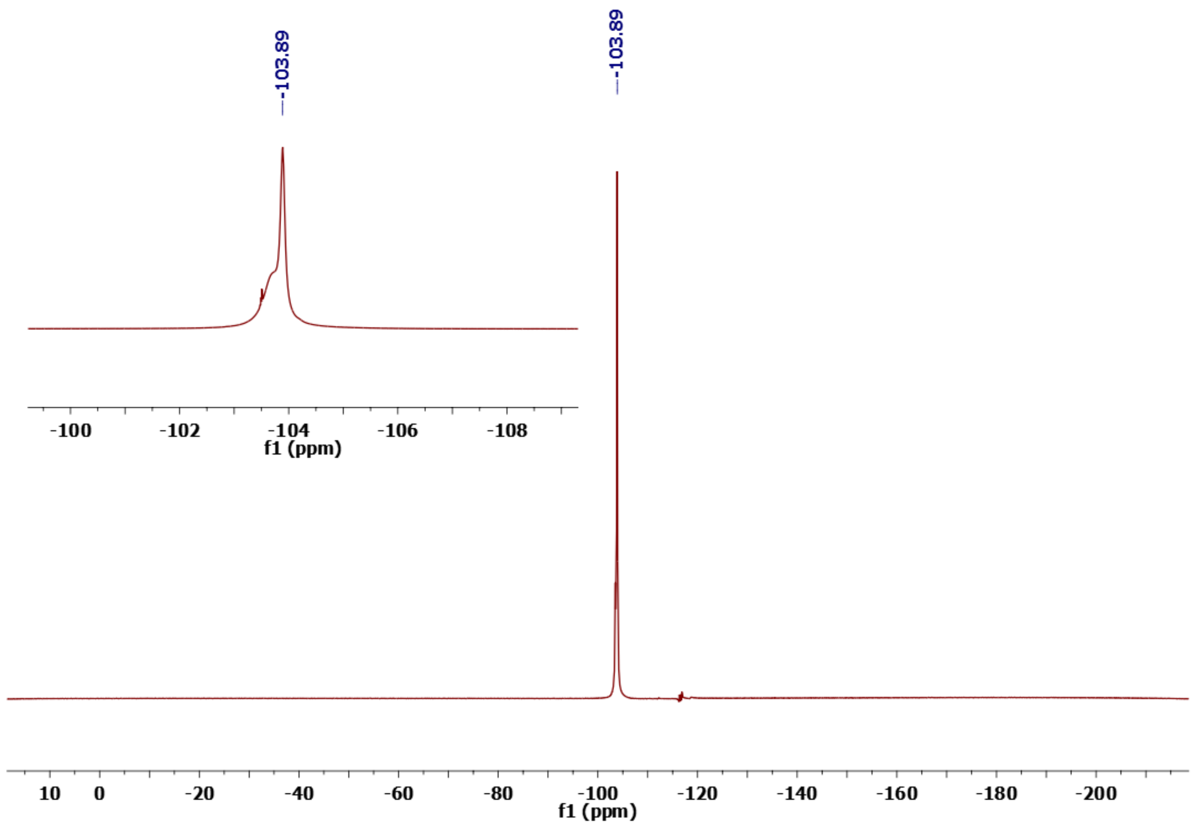

^{1}H NMR spectrum of (E)/(Z)-4-(4-cyanobenzylidene)-1-phenylpyrazolidine-3,5-dione (3f)

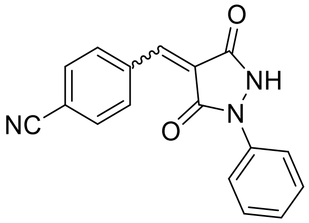

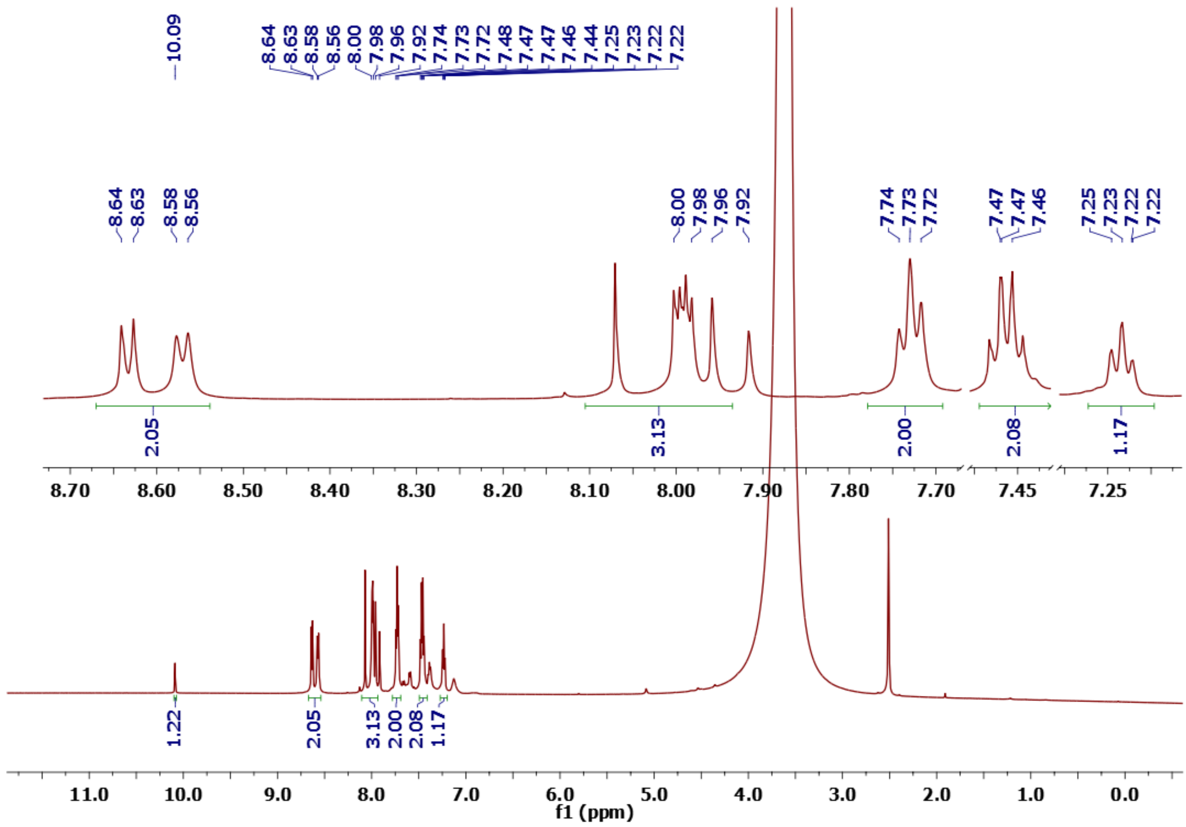

^{13}C NMR spectrum of (E)/(Z)-4-(4-cyanobenzylidene)-1-phenylpyrazolidine-3,5-dione (3f)

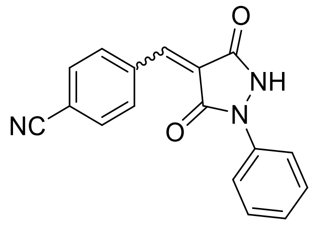

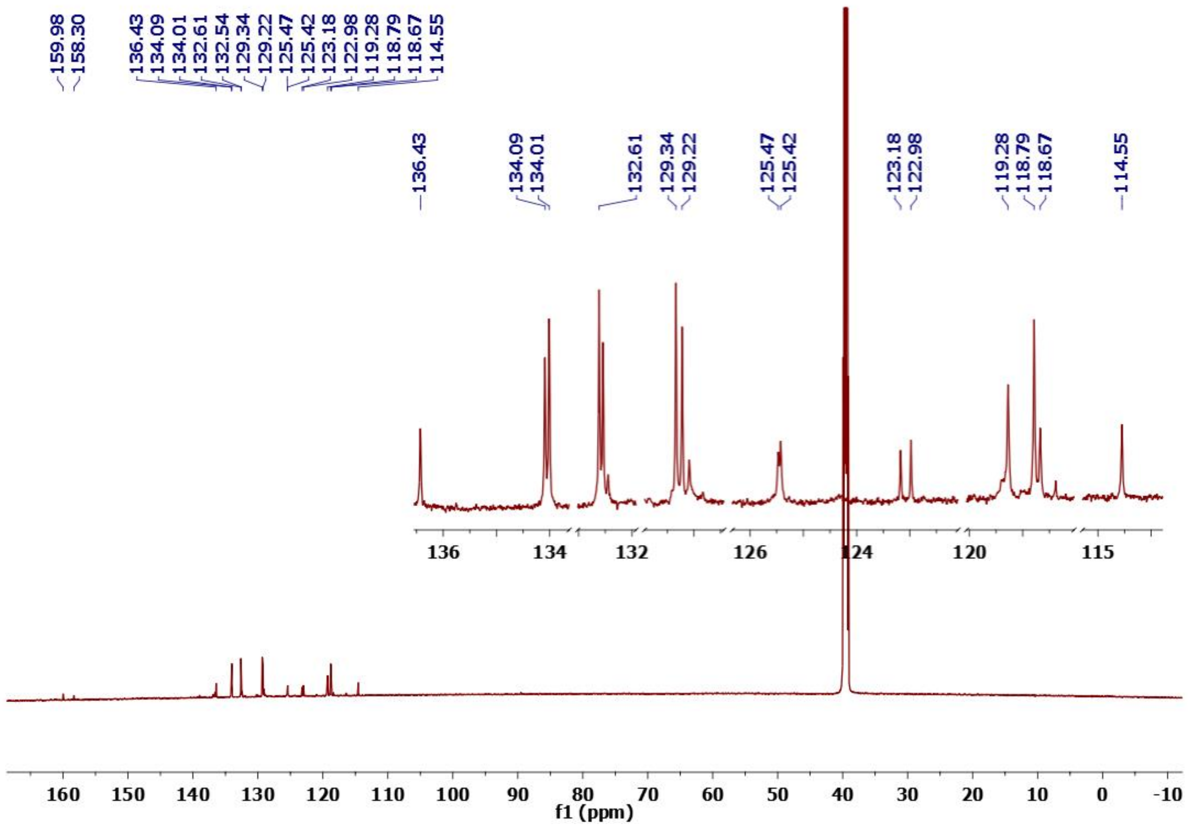

^{13}C DEPT-135 NMR spectrum of (E)/(Z)-4-(4-cyanobenzylidene)-1-phenylpyrazolidine-3,5-dione (3f)

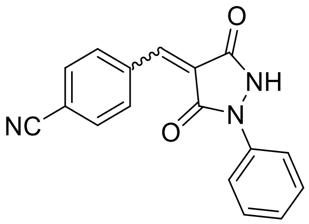

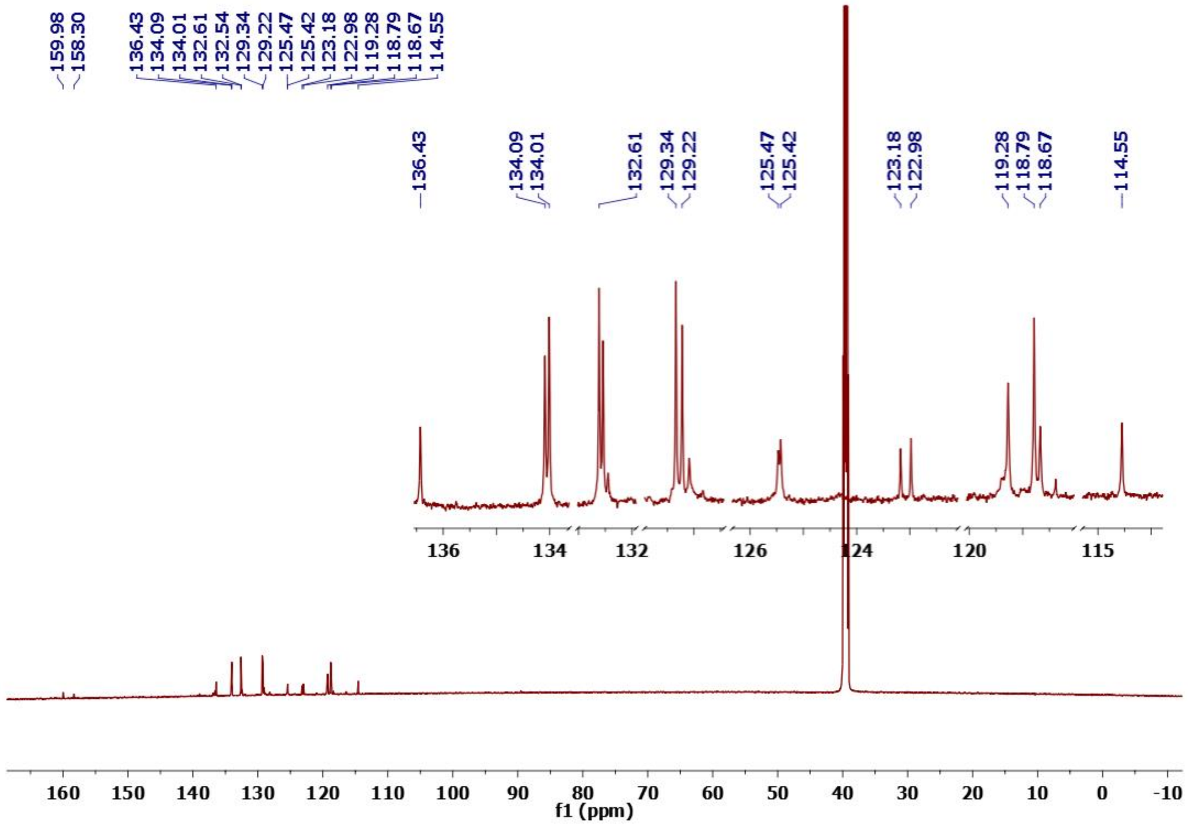

^{1}H NMR spectrum of (E)/(Z)-4-(4-nitrobenzylidene)-1-phenylpyrazolidine-3,5-dione (3g)

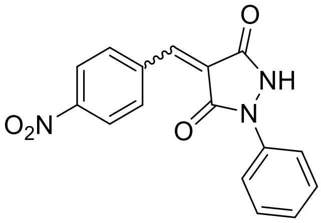

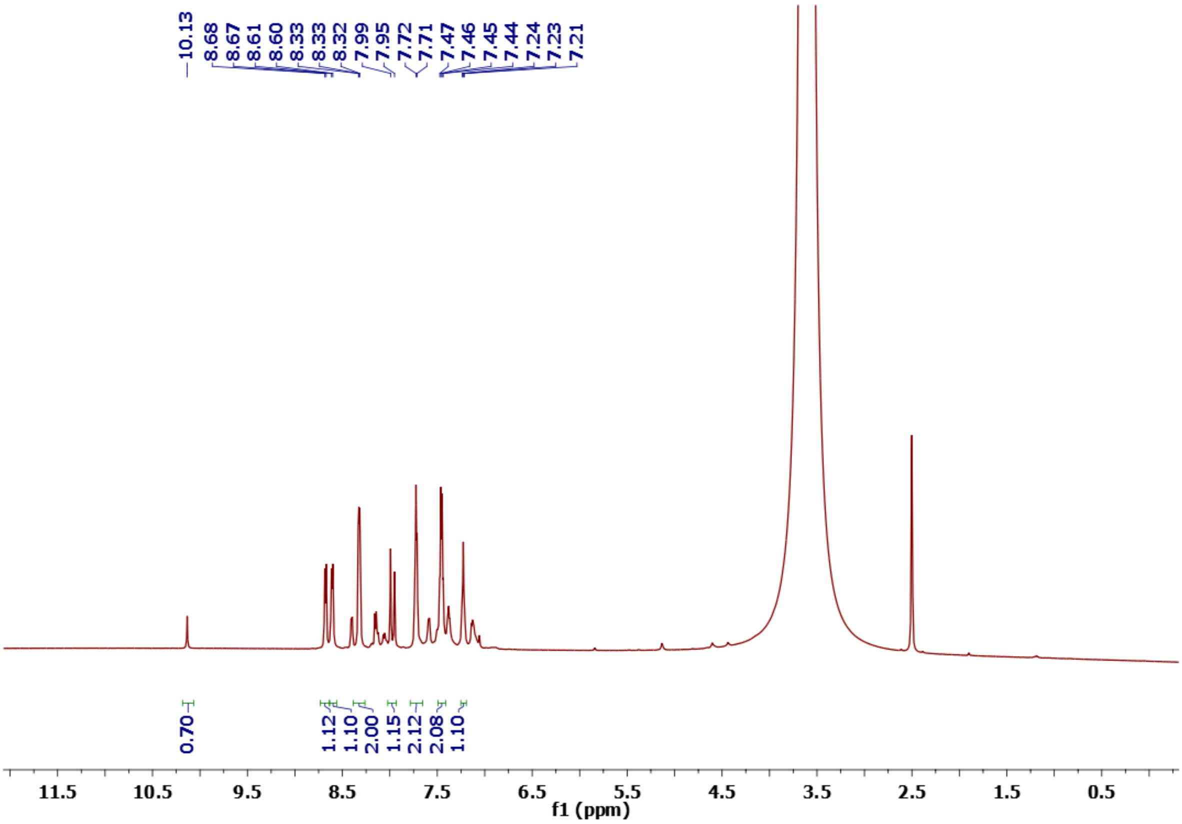

^{13}C NMR spectrum of (E)/(Z)-4-(4-nitrobenzylidene)-1-phenylpyrazolidine-3,5-dione (3g)

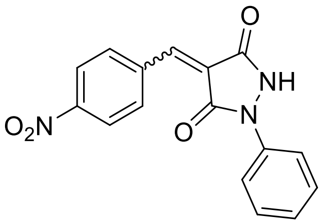

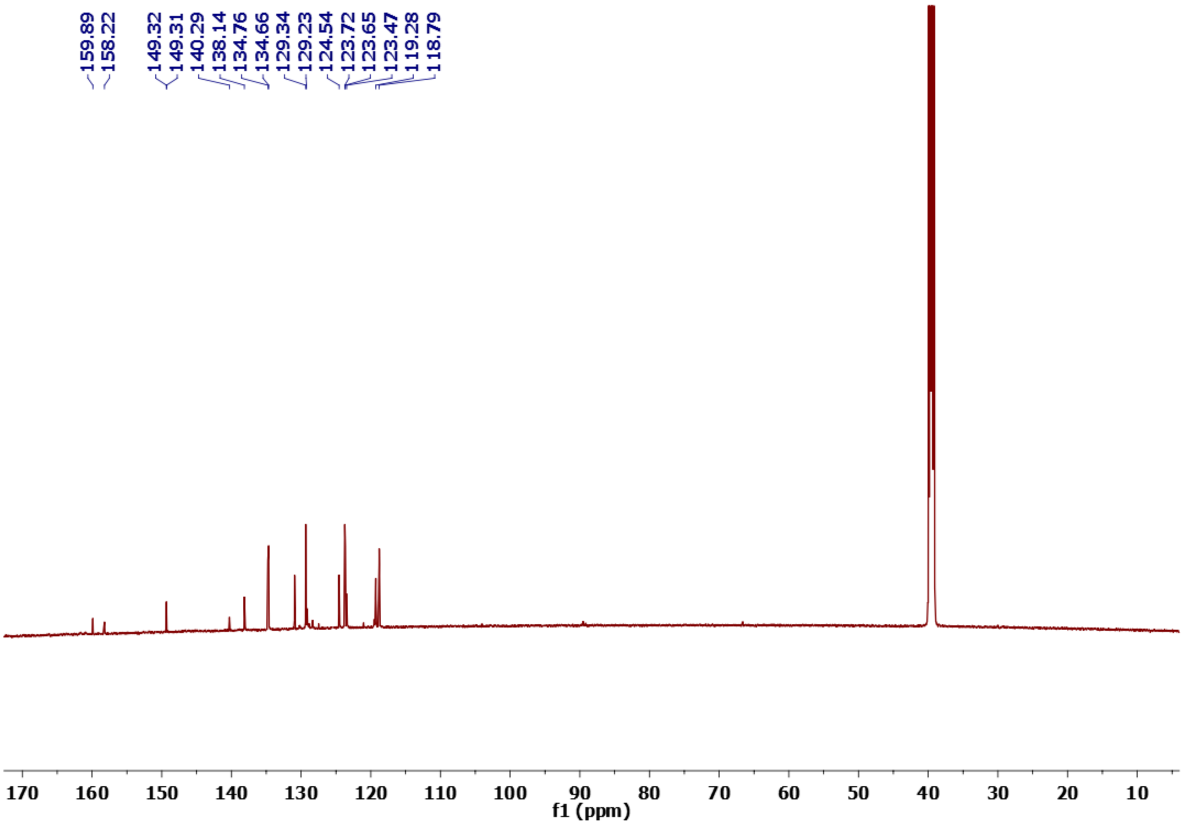

^{13}C DEPT-135 NMR spectrum of (E)/(Z)-4-(4-nitrobenzylidene)-1-phenylpyrazolidine-3,5-dione (3g)

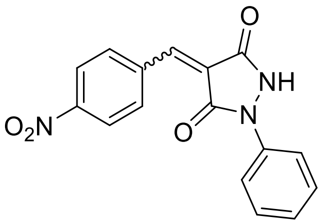

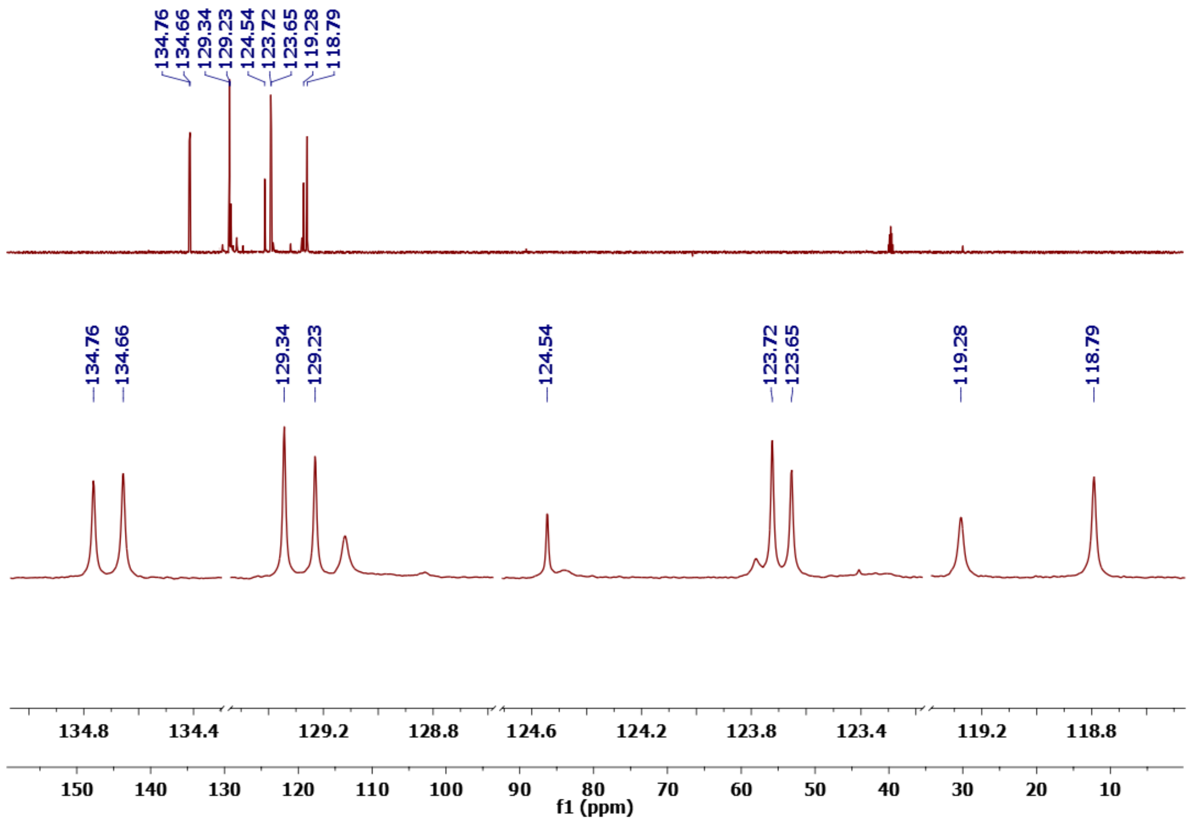

^{1}H NMR spectrum of (4'R,5'R)-1'',4'-diphenyl-4'H-dispiro[fluorene-9,3'-pyrazole-5',4''-pyrazolidine]-3'',5''-dione (5a) and (4'S,5'R)-1'',4'-diphenyl-4'H-dispiro[fluorene-9,3'-pyrazole-5',4''-pyrazolidine]-3'',5''-dione (5'a)

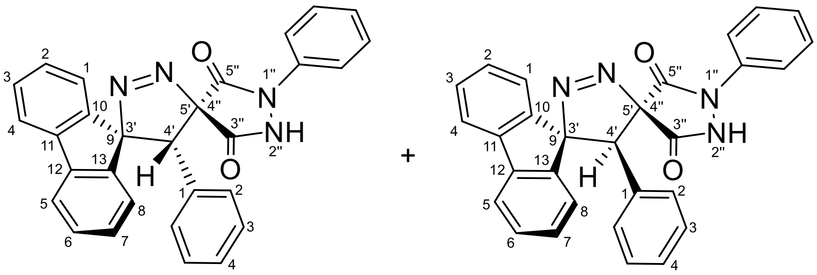

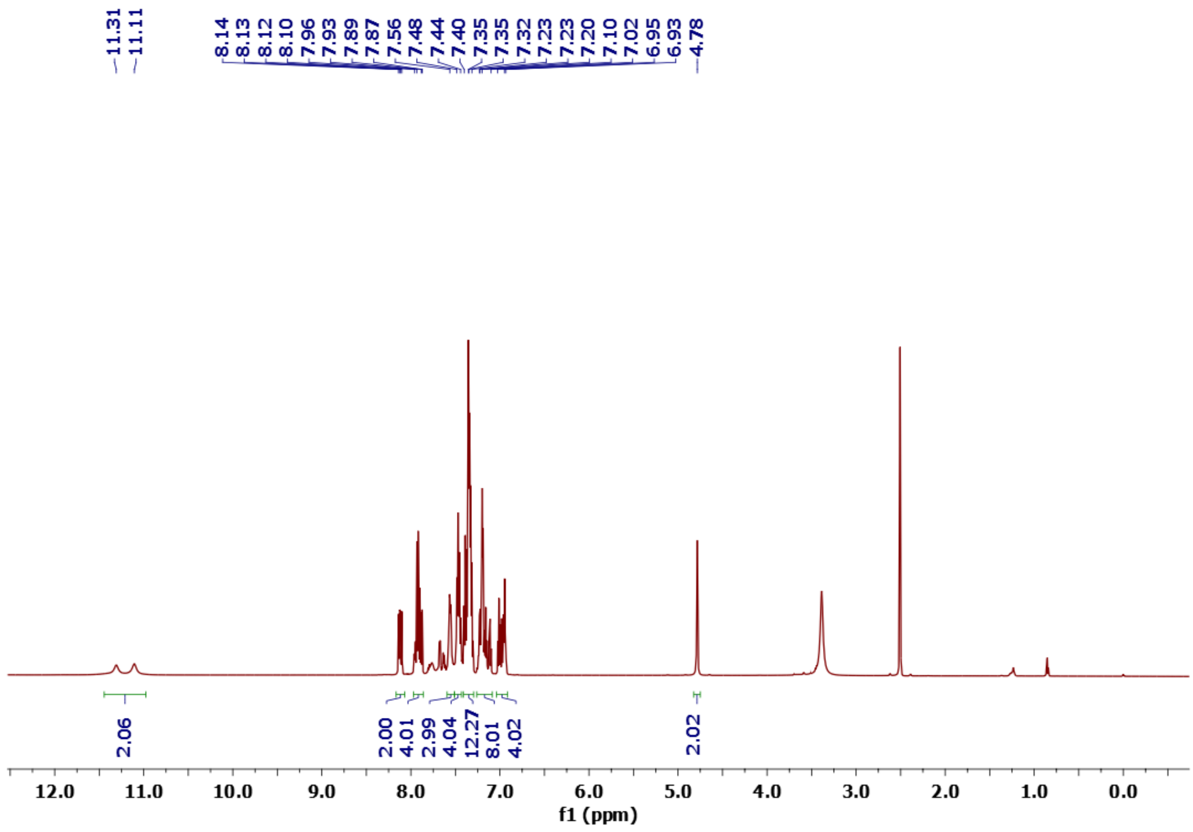

^{13}C NMR spectrum of (4'R,5'R)-1'',4'-diphenyl-4'H-dispiro[fluorene-9,3'-pyrazole-5',4''-pyrazolidine]-3'',5''-dione (5a) and (4'S,5'R)-1'',4'-diphenyl-4'H-dispiro[fluorene-9,3'-pyrazole-5',4''-pyrazolidine]-3'',5''-dione (5'a)

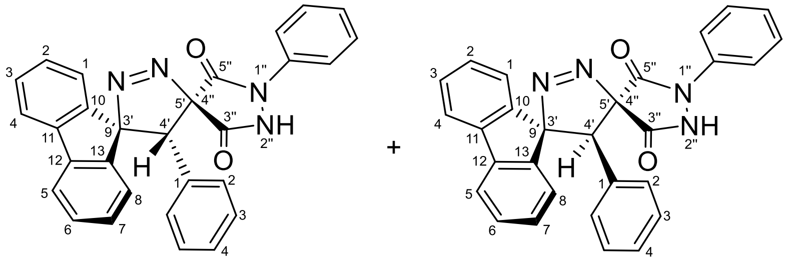

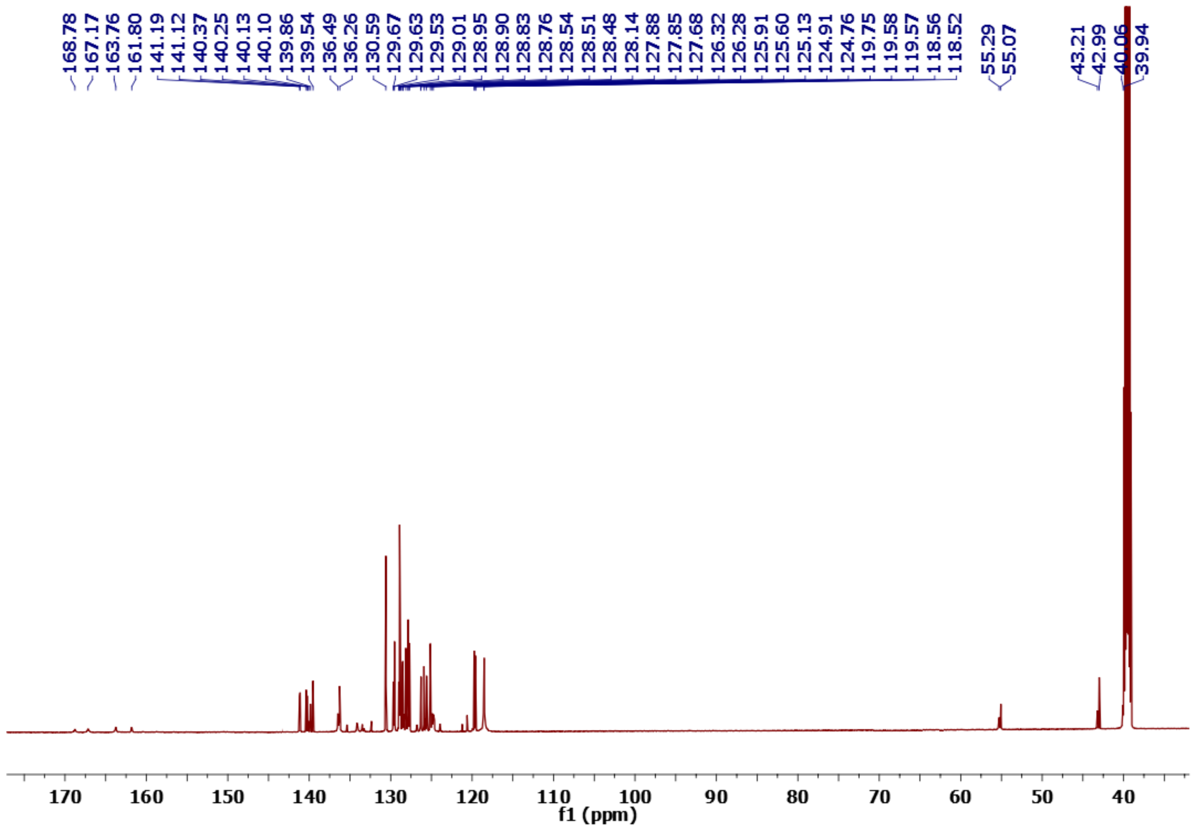

^{13}C DEPT-135 NMR spectrum of (4'R,5'R)-1'',4'-diphenyl-4'H-dispiro[fluorene-9,3'-pyrazole-5',4''-pyrazolidine]-3'',5''-dione (5a) and (4'S,5'R)-1'',4'-diphenyl-4'H-dispiro[fluorene-9,3'-pyrazole-5',4''-pyrazolidine]-3'',5''-dione (5'a)

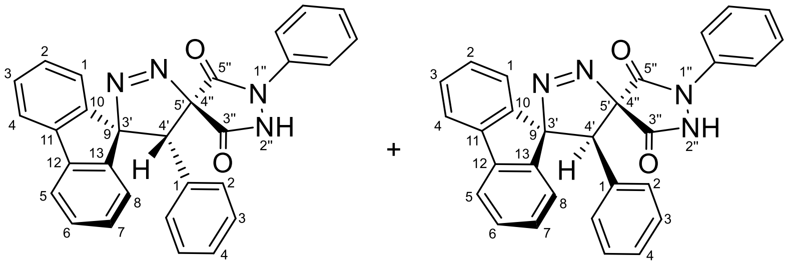

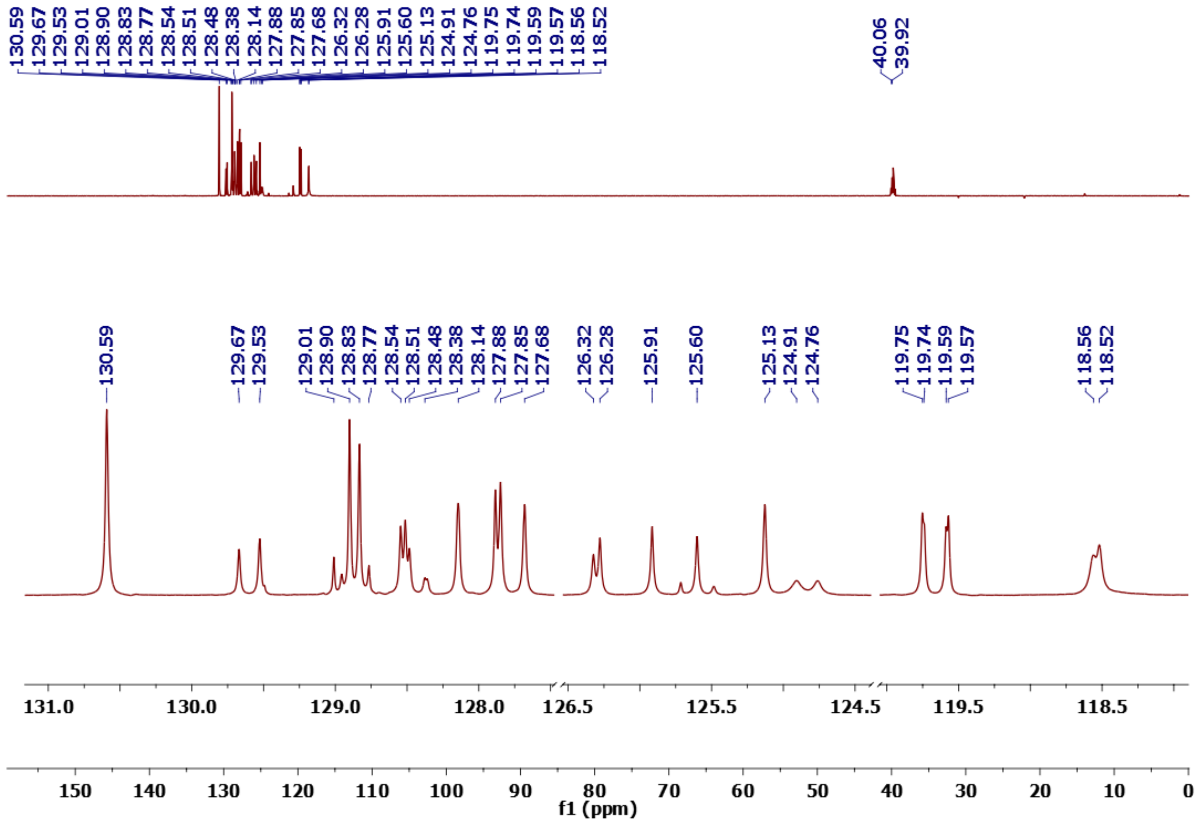

^{1}H-^{1}H gDQCOSY NMR spectrum of (4'R,5'R)-1'',4'-diphenyl-4'H-dispiro[fluorene-9,3'-pyrazole-5',4''-pyrazolidine]-3'',5''-dione (5a) and (4'S,5'R)-1'',4'-diphenyl-4'H-dispiro[fluorene-9,3'-pyrazole-5',4''-pyrazolidine]-3'',5''-dione (5'a)

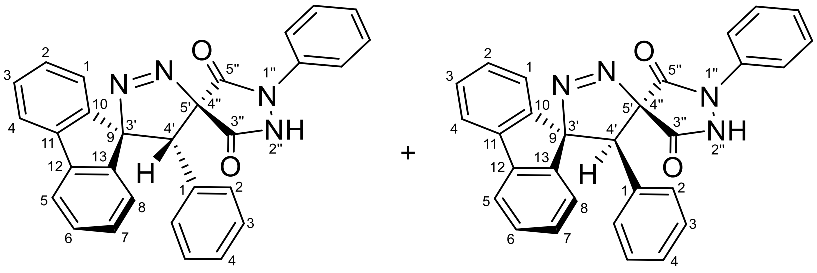

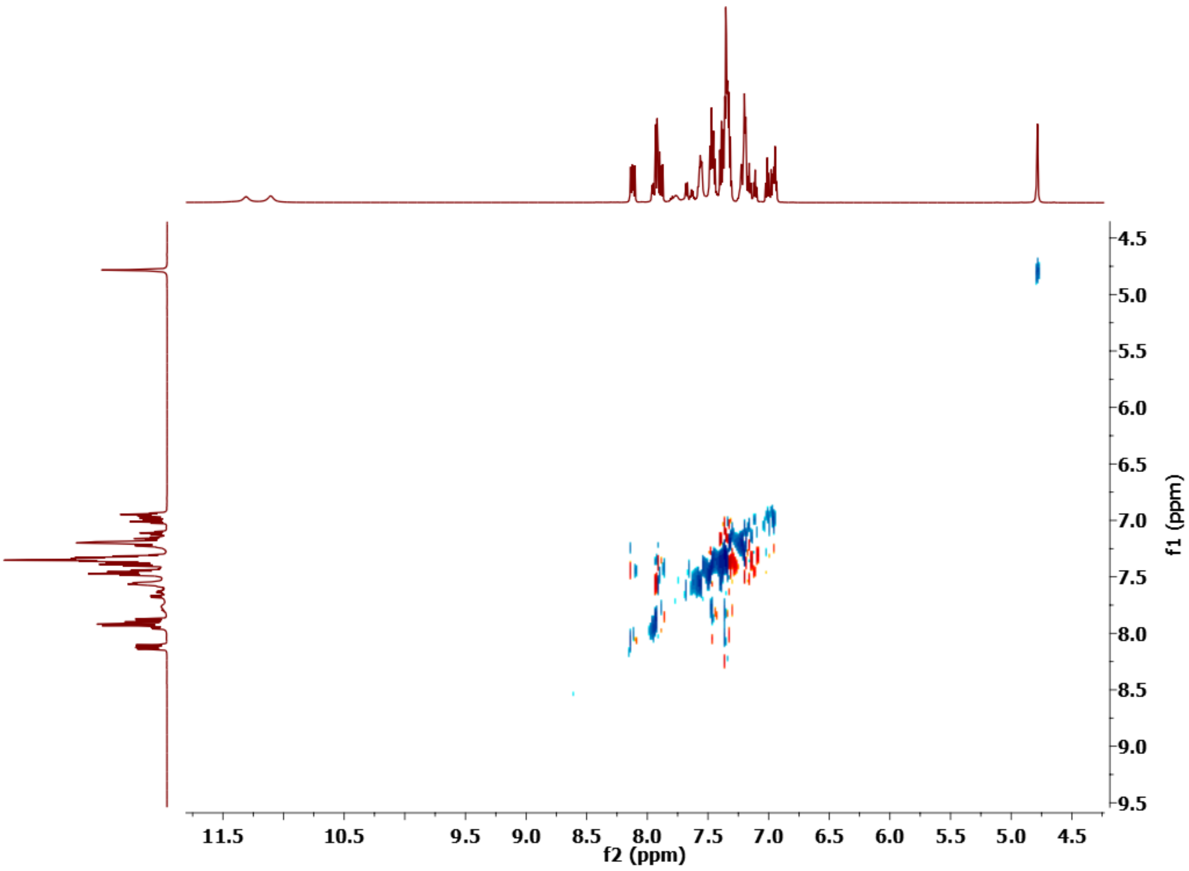

^{1}H-^{13}C-HSQC NMR spectrum of (4'R,5'R)-1'',4'-diphenyl-4'H-dispiro[fluorene-9,3'-pyrazole-5',4''-pyrazolidine]-3'',5''-dione (5a) and (4'S,5'R)-1'',4'-diphenyl-4'H-dispiro[fluorene-9,3'-pyrazole-5',4''-pyrazolidine]-3'',5''-dione (5'a)

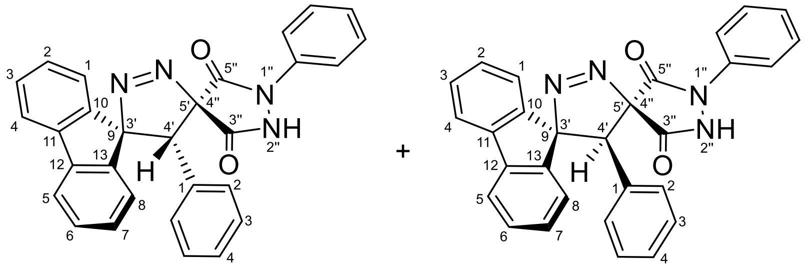

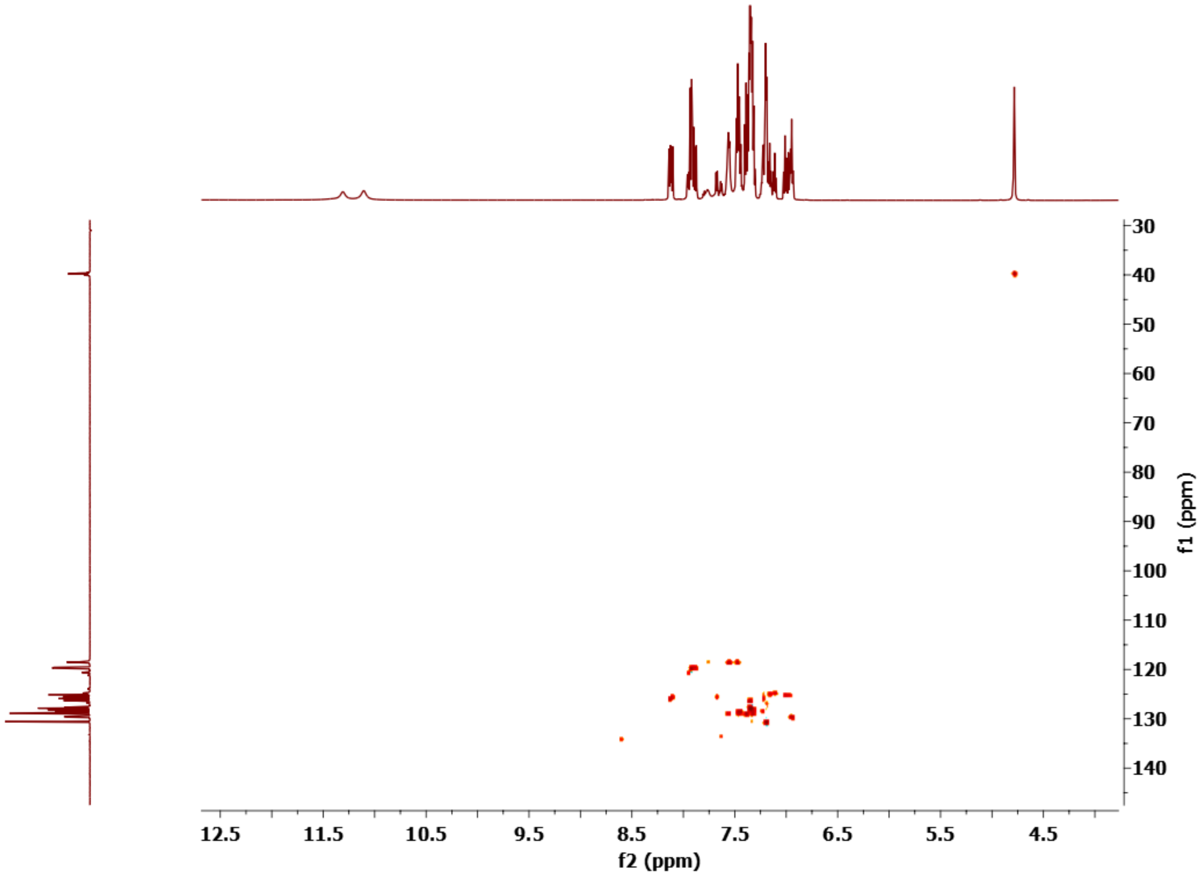

^{1}H-^{13}C-gHMBC NMR spectrum of (4'R,5'R)-1'',4'-diphenyl-4'H-dispiro[fluorene-9,3'-pyrazole-5',4''-pyrazolidine]-3'',5''-dione (5a) and (4'S,5'R)-1'',4'-diphenyl-4'H-dispiro[fluorene-9,3'-pyrazole-5',4''-pyrazolidine]-3'',5''-dione (5'a)

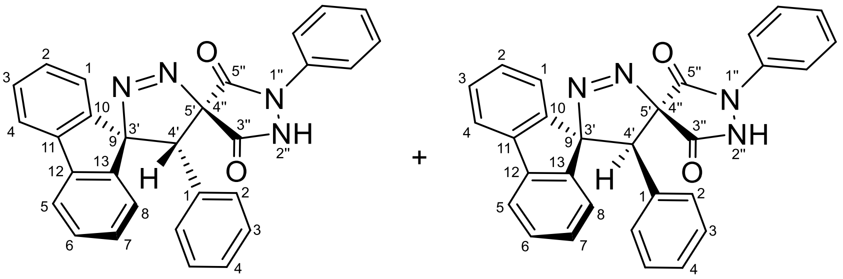

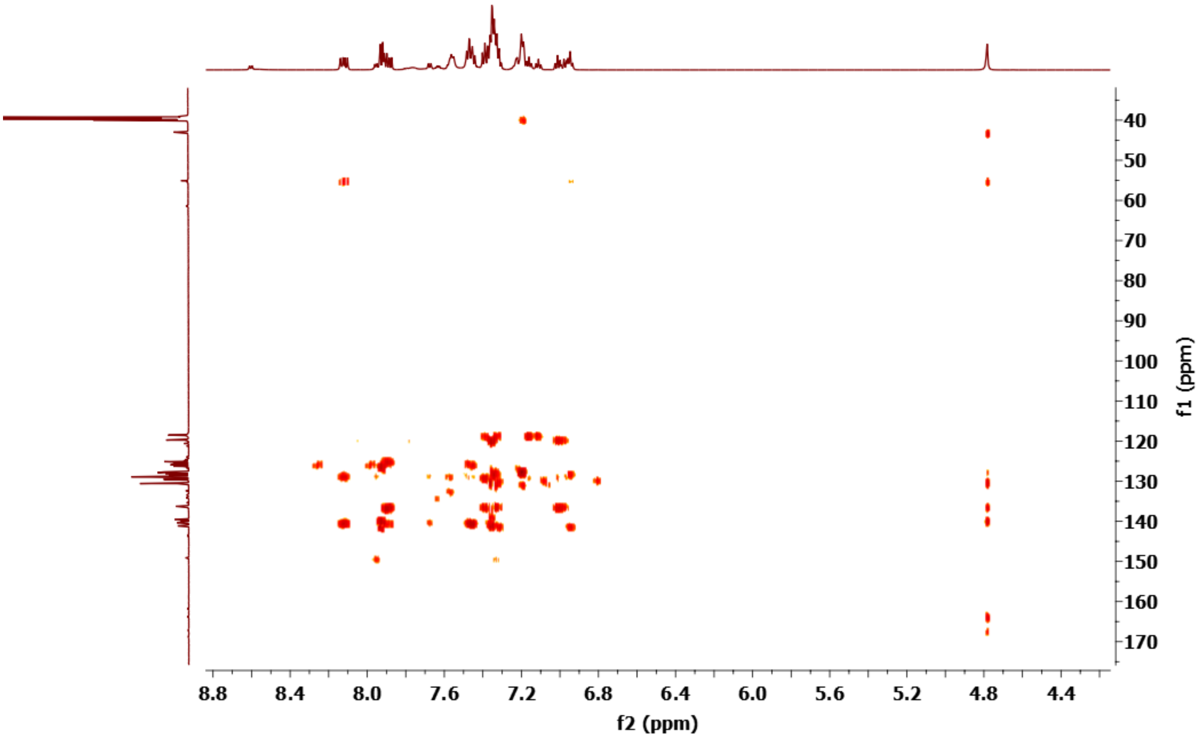

^{1}H NMR spectrum of (4'R,5'R)-1''-phenyl-4'-(4-methoxyphenyl)-4'H-dispiro[fluorene-9,3'-pyrazole-5',4''-pyrazolidine]-3'',5''-dione (5b) and (4'S,5'R)-1''-phenyl-4'-(4-methoxyphenyl)-4'H-dispiro[fluorene-9,3'-pyrazole-5',4''-pyrazolidine]-3'',5''-dione (5'b)

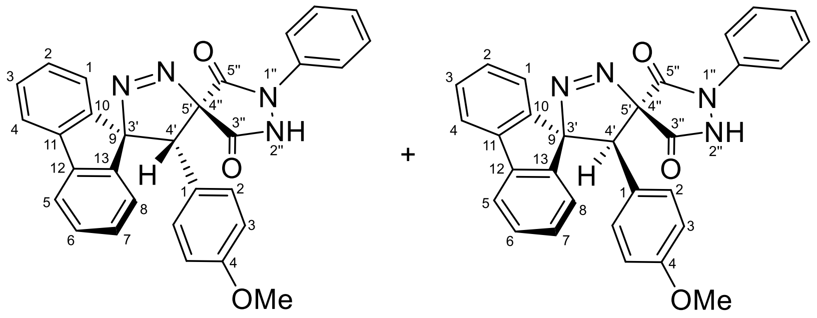

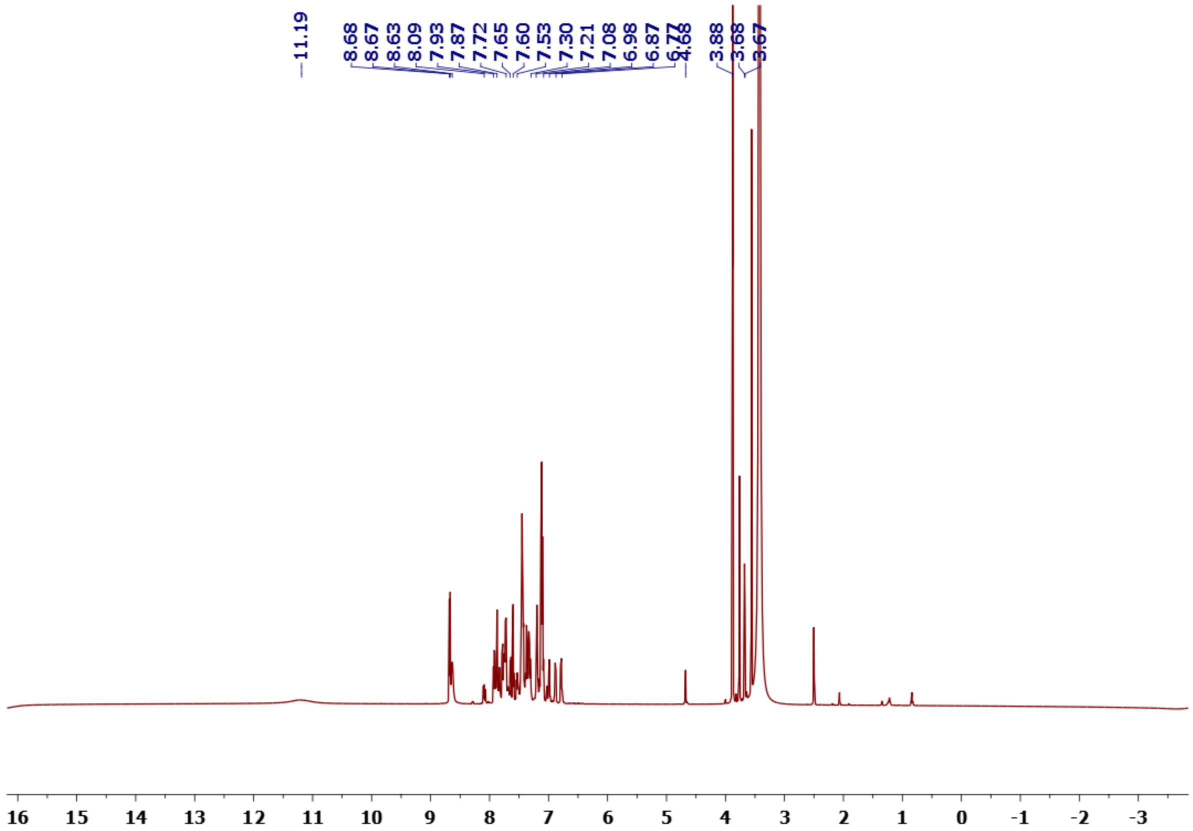

^{13}C NMR spectrum of (4'R,5'R)-1''-phenyl-4'-(4-methoxyphenyl)-4'H-dispiro[fluorene-9,3'-pyrazole-5',4''-pyrazolidine]-3'',5''-dione (5b) and (4'S,5'R)-1''-phenyl-4'-(4-methoxyphenyl)-4'H-dispiro[fluorene-9,3'-pyrazole-5',4''-pyrazolidine]-3'',5''-dione (5'b)

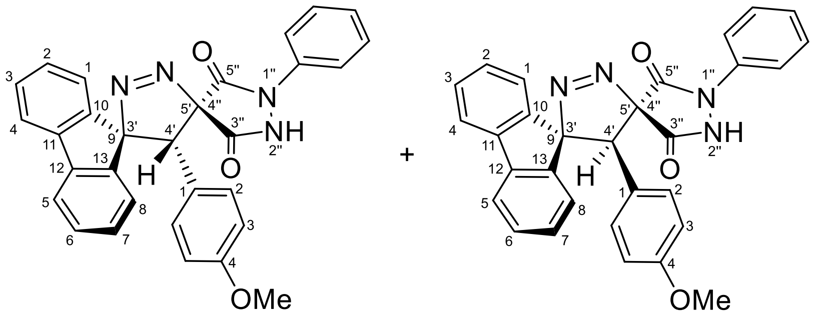

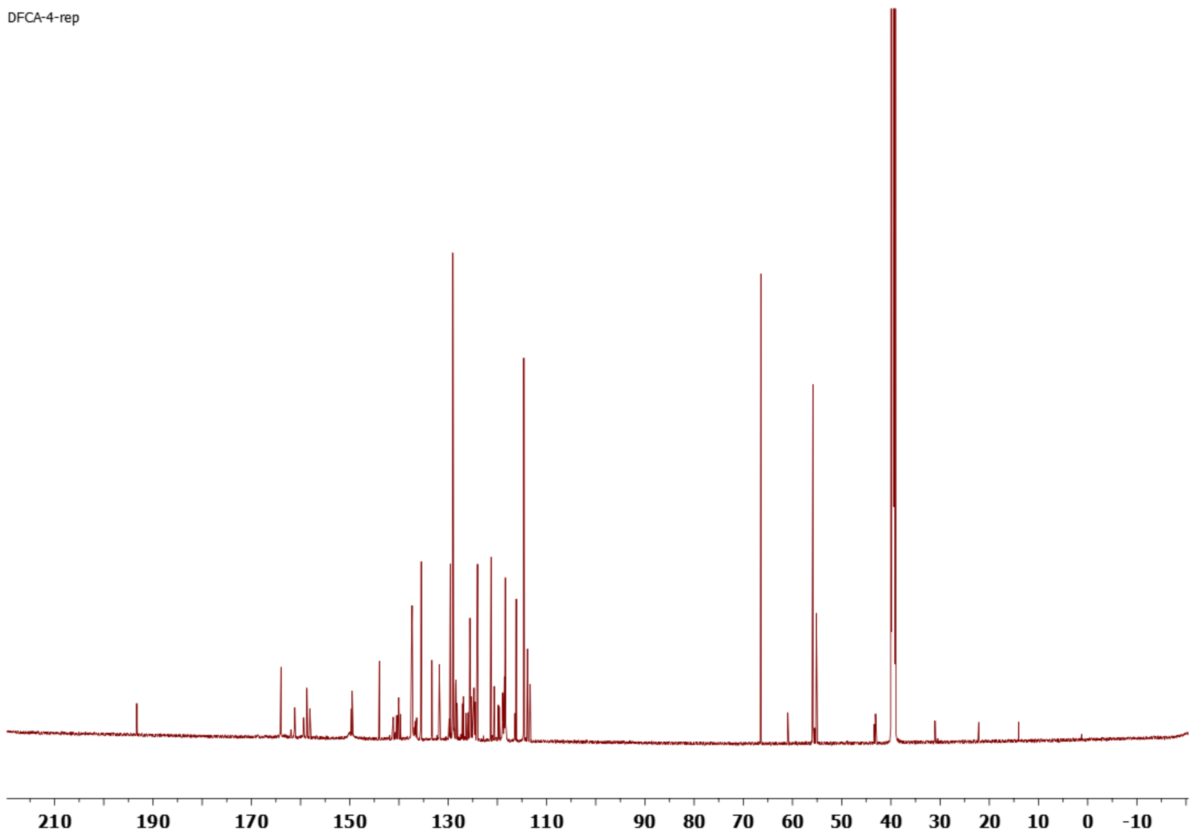

^{1}H NMR spectrum of (4'R,5'R)-1''-phenyl-4'-(p-tolyl)-4'H-dispiro[fluorene-9,3'-pyrazole-5',4''-pyrazolidine]-3'',5''-dione (5c) and (4'S,5'R)-1''-phenyl-4'-(p-tolyl)-4'H-dispiro[fluorene-9,3'-pyrazole-5',4''-pyrazolidine]-3'',5''-dione (5'c)

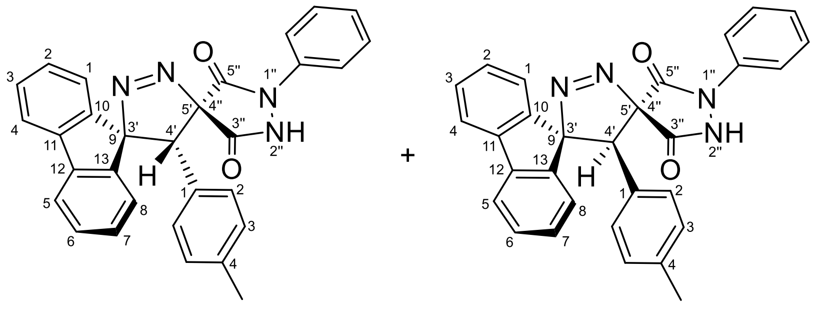

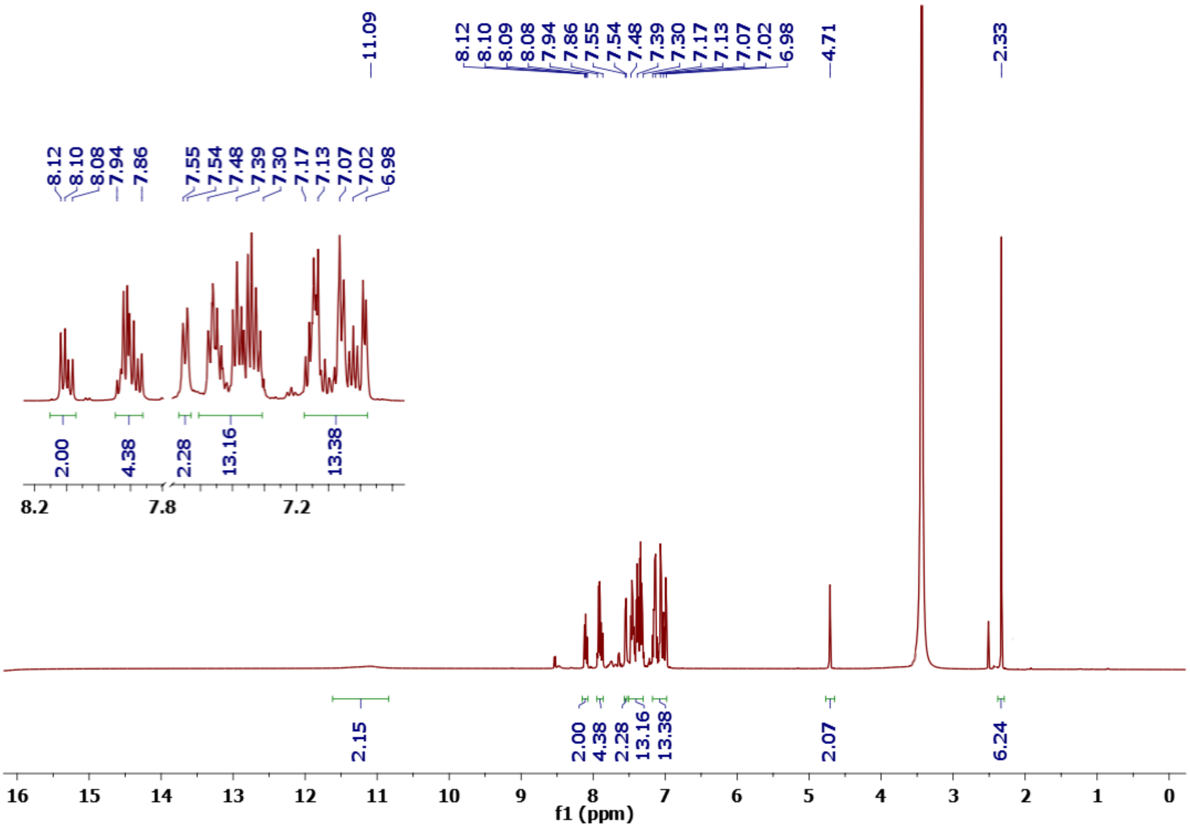

^{13}C NMR spectrum of (4'R,5'R)-1''-phenyl-4'-(p-tolyl)-4'H-dispiro[fluorene-9,3'-pyrazole-5',4''-pyrazolidine]-3'',5''-dione (5c) and (4'S,5'R)-1''-phenyl-4'-(p-tolyl)-4'H-dispiro[fluorene-9,3'-pyrazole-5',4''-pyrazolidine]-3'',5''-dione (5'c)

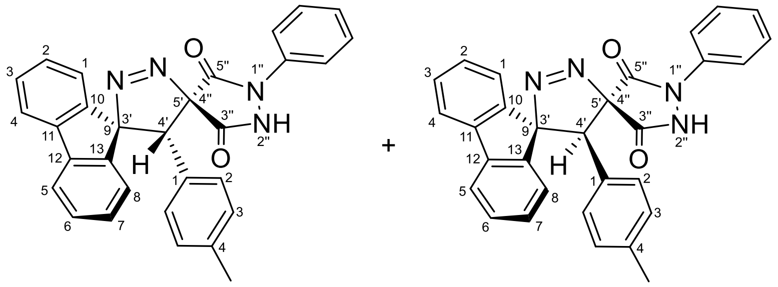

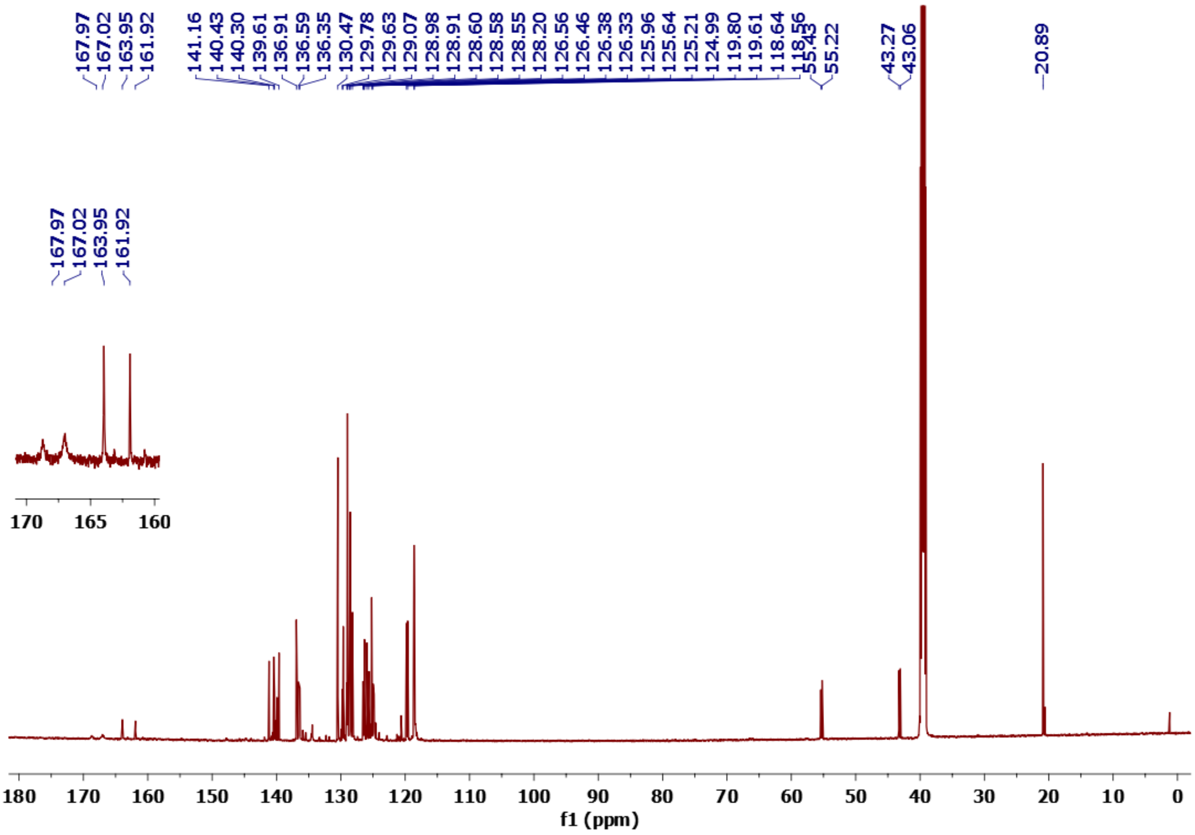

^{13}C DEPT-135 NMR spectrum of (4'R,5'R)-1''-phenyl-4'-(p-tolyl)-4'H-dispiro[fluorene-9,3'-pyrazole-5',4''-pyrazolidine]-3'',5''-dione (5c) and (4'S,5'R)-1''-phenyl-4'-(p-tolyl)-4'H-dispiro[fluorene-9,3'-pyrazole-5',4''-pyrazolidine]-3'',5''-dione (5'c)

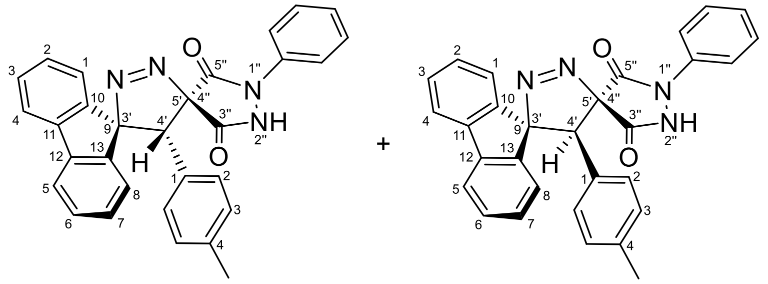

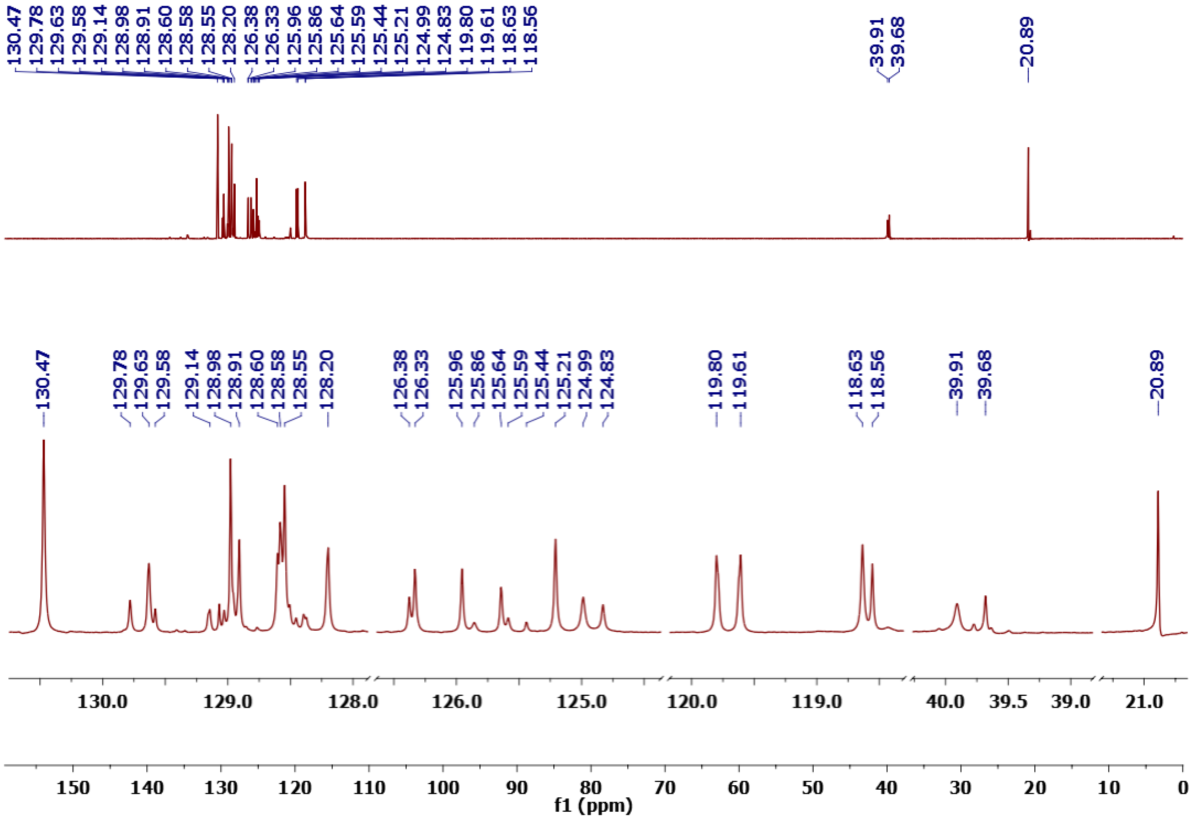

^{1}H-^{1}H gDQCOSY NMR spectrum of (4'R,5'R)-1''-phenyl-4'-(p-tolyl)-4'H-dispiro[fluorene-9,3'-pyrazole-5',4''-pyrazolidine]-3'',5''-dione (5c) and (4'S,5'R)-1''-phenyl-4'-(p-tolyl)-4'H-dispiro[fluorene-9,3'-pyrazole-5',4''-pyrazolidine]-3'',5''-dione (5'c)

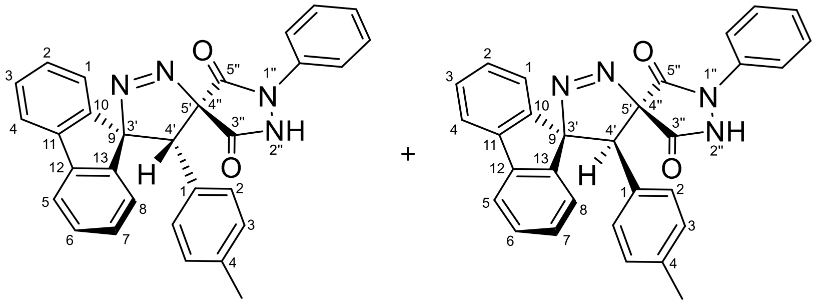

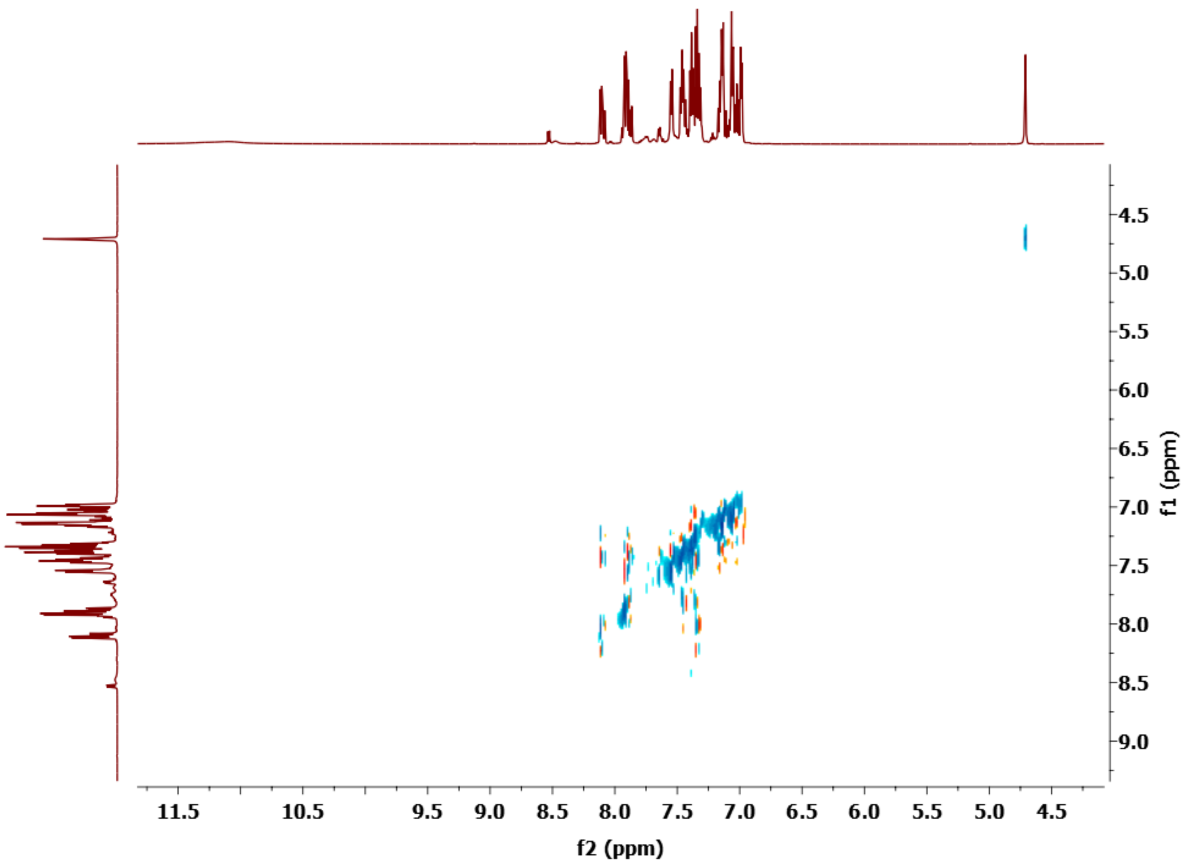

^{1}H-^{13}C-HSQC NMR spectrum of (4'R,5'R)-1''-phenyl-4'-(p-tolyl)-4'H-dispiro[fluorene-9,3'-pyrazole-5',4''-pyrazolidine]-3'',5''-dione (5c) and (4'S,5'R)-1''-phenyl-4'-(p-tolyl)-4'H-dispiro[fluorene-9,3'-pyrazole-5',4''-pyrazolidine]-3'',5''-dione (5'c)

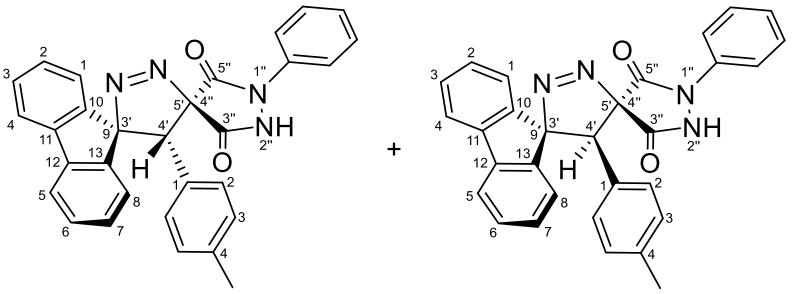

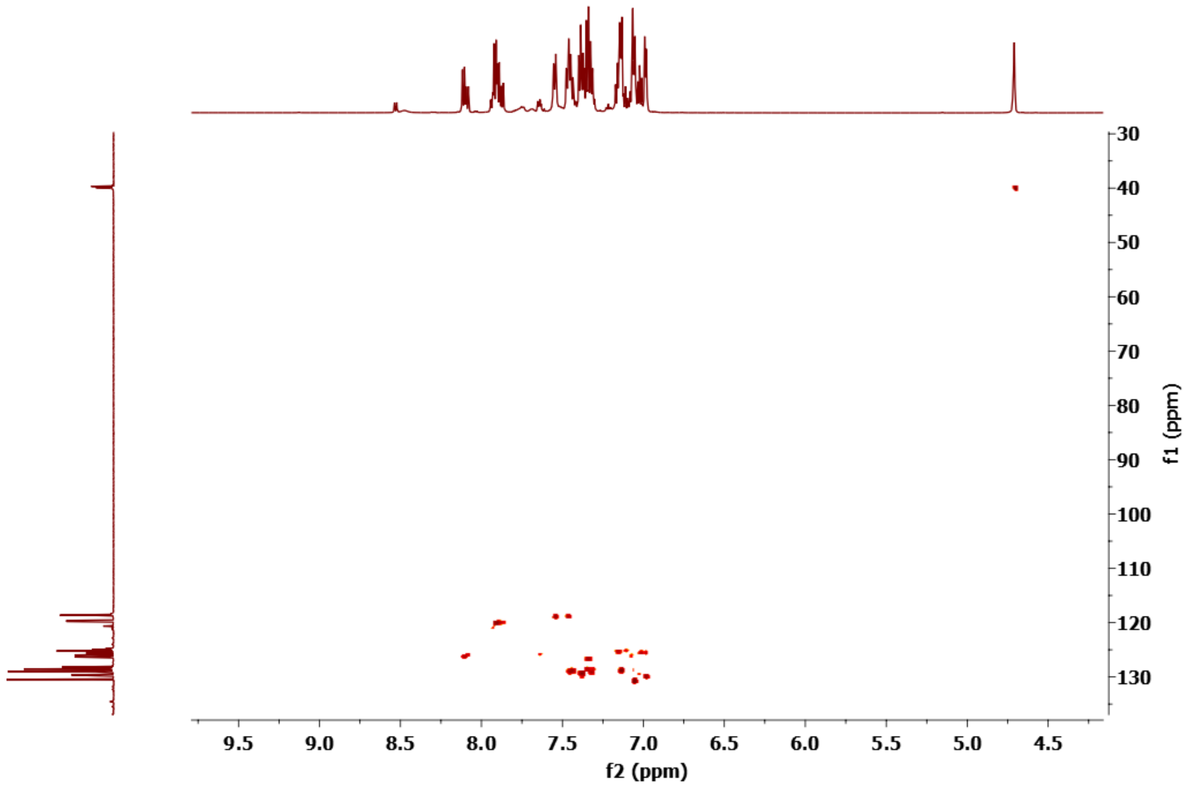

^{1}H-^{13}C-gHMBC spectrum of (4'R,5'R)-1''-phenyl-4'-(p-tolyl)-4'H-dispiro[fluorene-9,3'-pyrazole-5',4''-pyrazolidine]-3'',5''-dione (5c) and (4'S,5'R)-1''-phenyl-4'-(p-tolyl)-4'H-dispiro[fluorene-9,3'-pyrazole-5',4''-pyrazolidine]-3'',5''-dione (5'c)

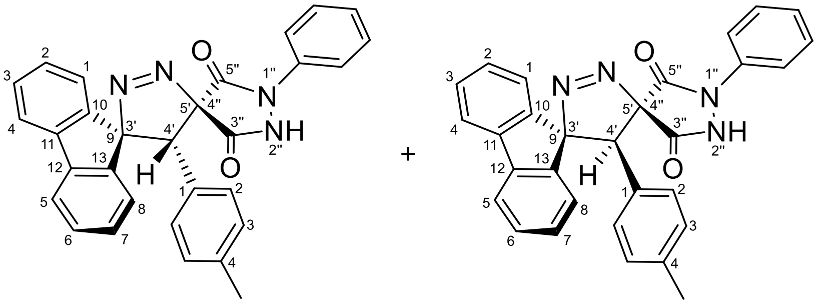

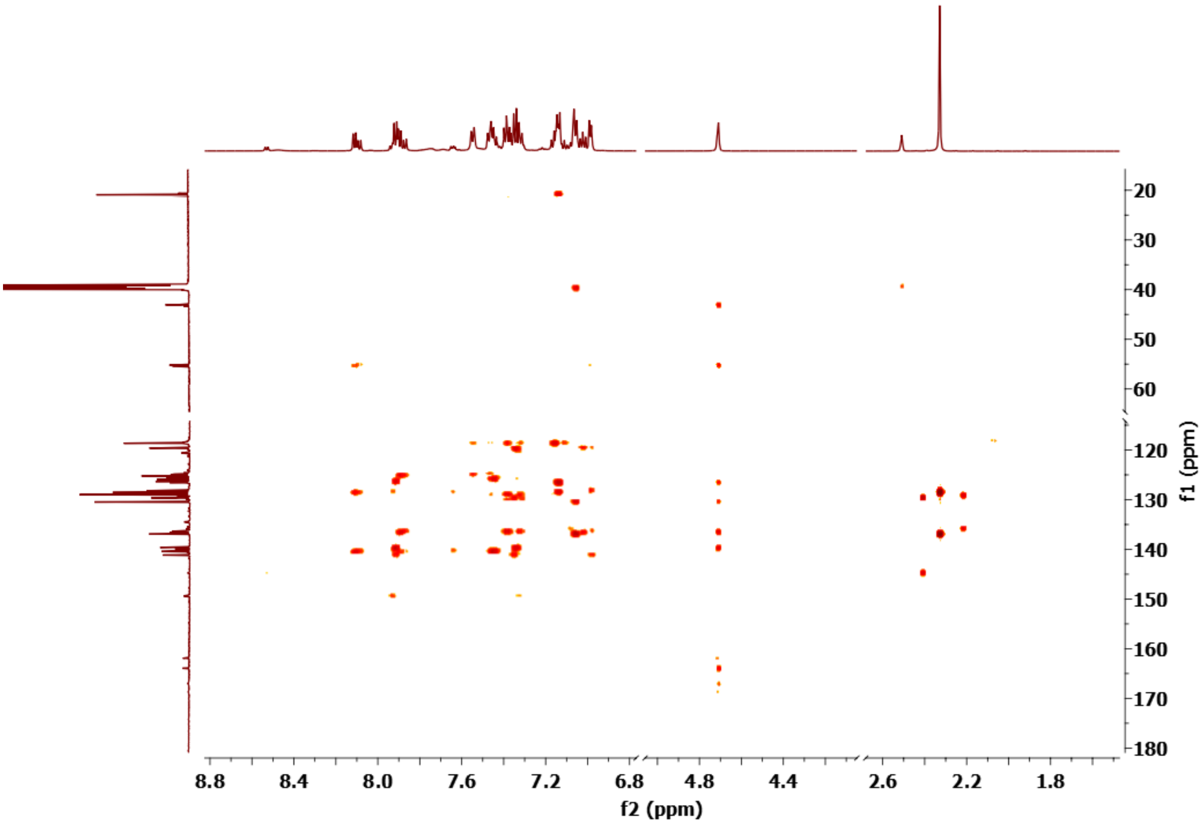

^{1}H NMR spectrum of (4'R,5'R)-4'-(4-chlorophenyl)-1''-phenyl-4'H-dispiro[fluorene-9,3'-pyrazole-5',4''-pyrazolidine]-3'',5''-dione (5d) and (4'S,5'R)-4'-(4-chlorophenyl)-1''-phenyl-4'H-dispiro[fluorene-9,3'-pyrazole-5',4''-pyrazolidine]-3'',5''-dione (5'd)

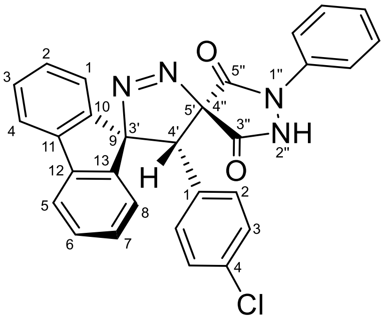

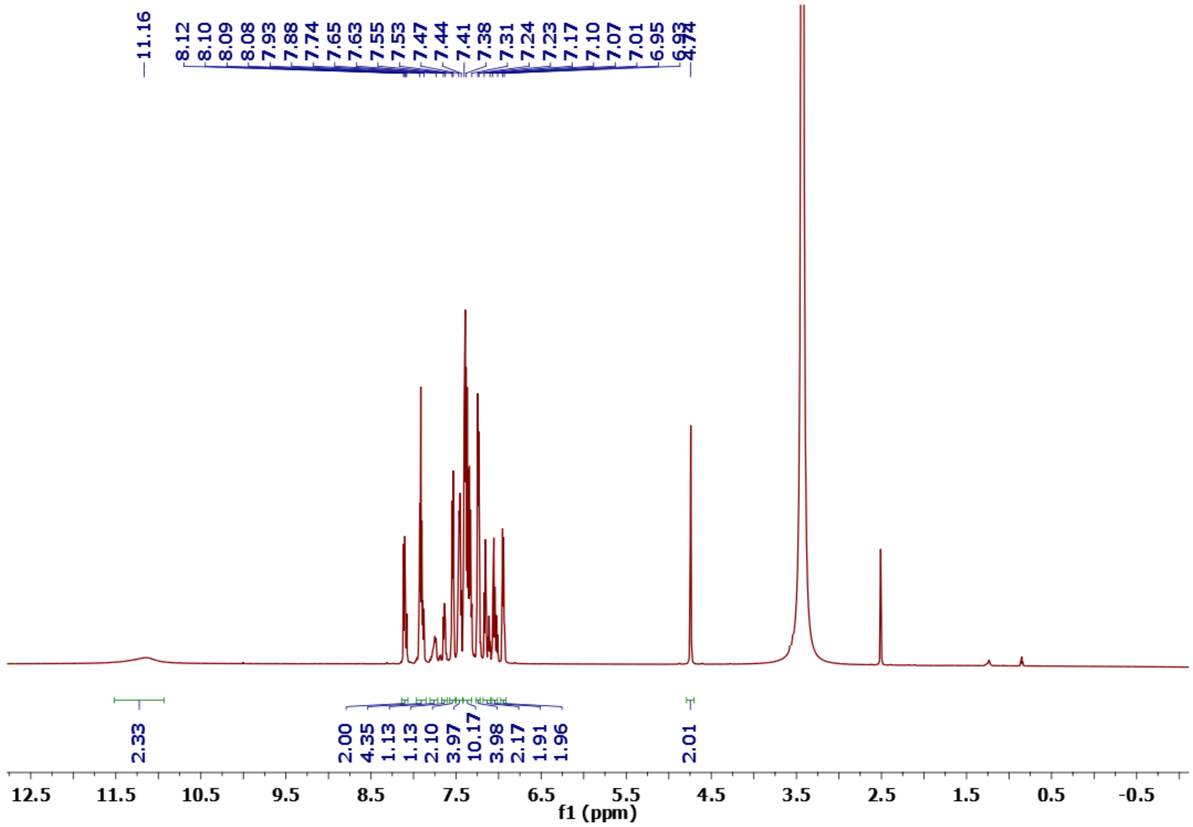

^{13}C NMR spectrum of (4'R,5'R)-4'-(4-chlorophenyl)-1''-phenyl-4'H-dispiro[fluorene-9,3'-pyrazole-5',4''-pyrazolidine]-3'',5''-dione (5d) and (4'S,5'R)-4'-(4-chlorophenyl)-1''-phenyl-4'H-dispiro[fluorene-9,3'-pyrazole-5',4''-pyrazolidine]-3'',5''-dione (5'd)

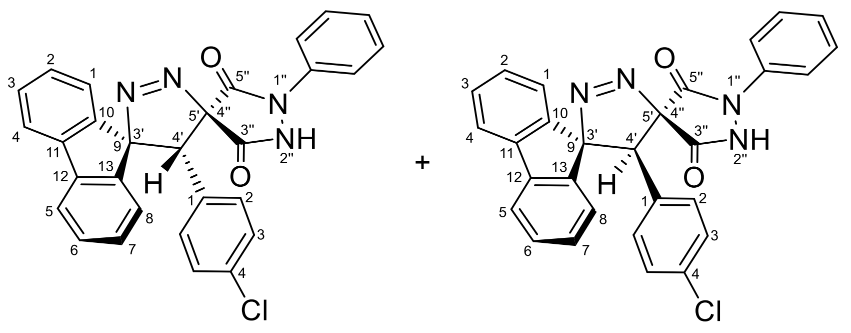

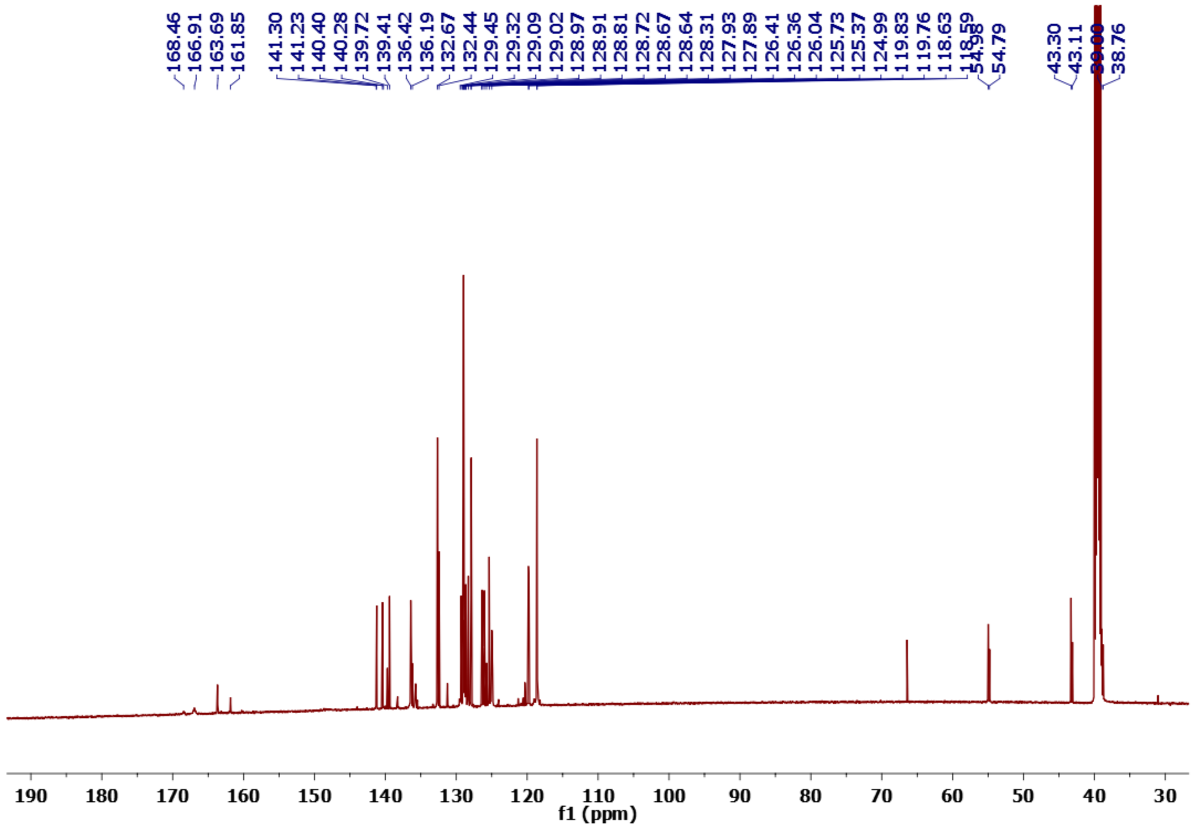

^{13}C DEPT-135 NMR spectrum of (4'R,5'R)-4'-(4-chlorophenyl)-1''-phenyl-4'H-dispiro[fluorene-9,3'-pyrazole-5',4''-pyrazolidine]-3'',5''-dione (5d) and (4'S,5'R)-4'-(4-chlorophenyl)-1''-phenyl-4'H-dispiro[fluorene-9,3'-pyrazole-5',4''-pyrazolidine]-3'',5''-dione (5'd)

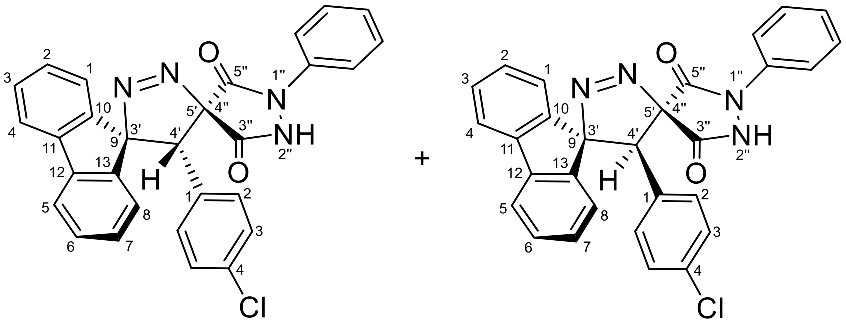

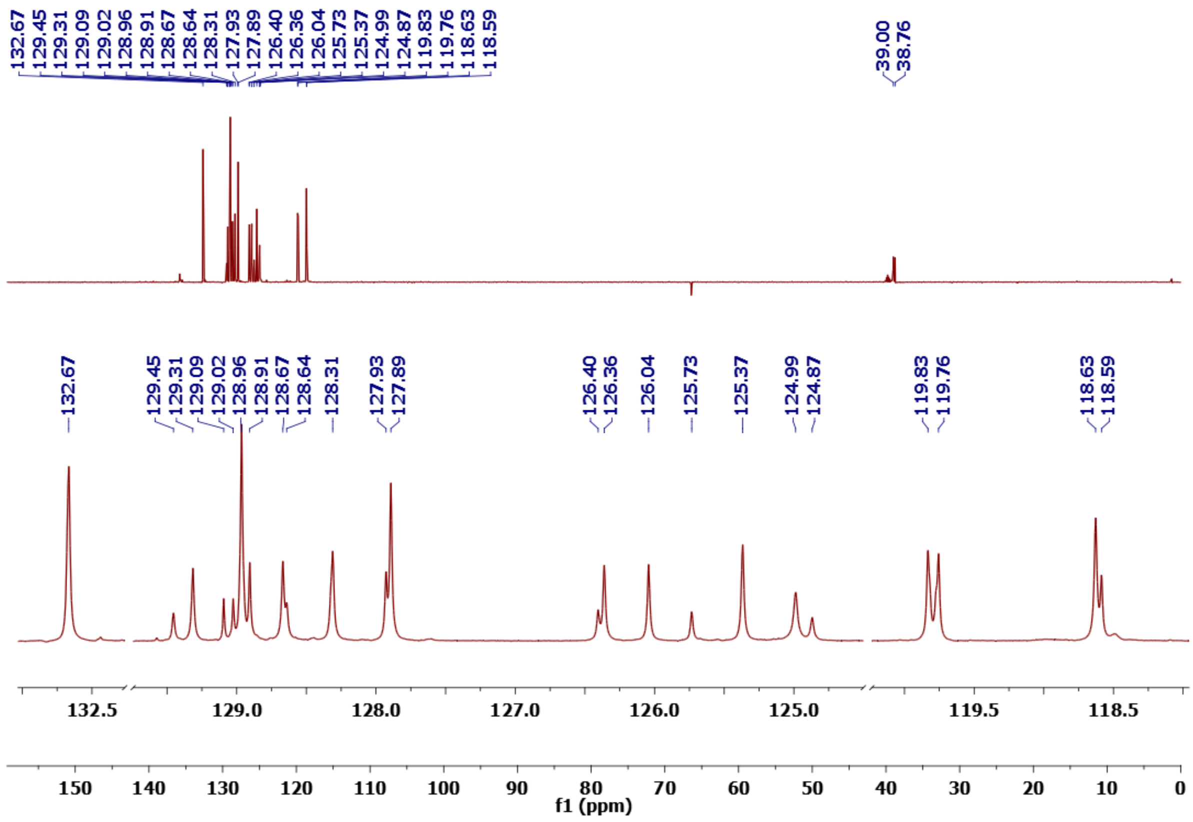

^{1}H-^{1}H gDQCOSY NMR spectrum of (4'R,5'R)-4'-(4-chlorophenyl)-1''-phenyl-4'H-dispiro[fluorene-9,3'-pyrazole-5',4''-pyrazolidine]-3'',5''-dione (5d) and (4'S,5'R)-4'-(4-chlorophenyl)-1''-phenyl-4'H-dispiro[fluorene-9,3'-pyrazole-5',4''-pyrazolidine]-3'',5''-dione (5'd)

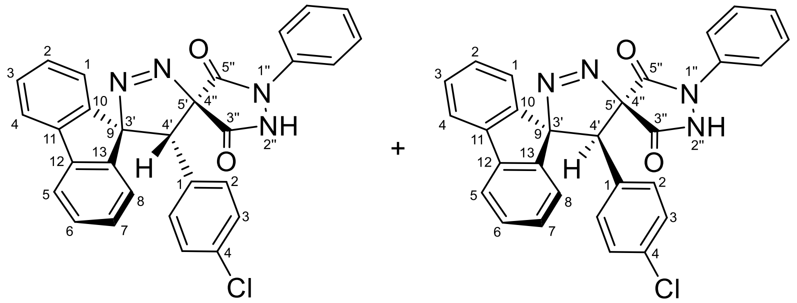

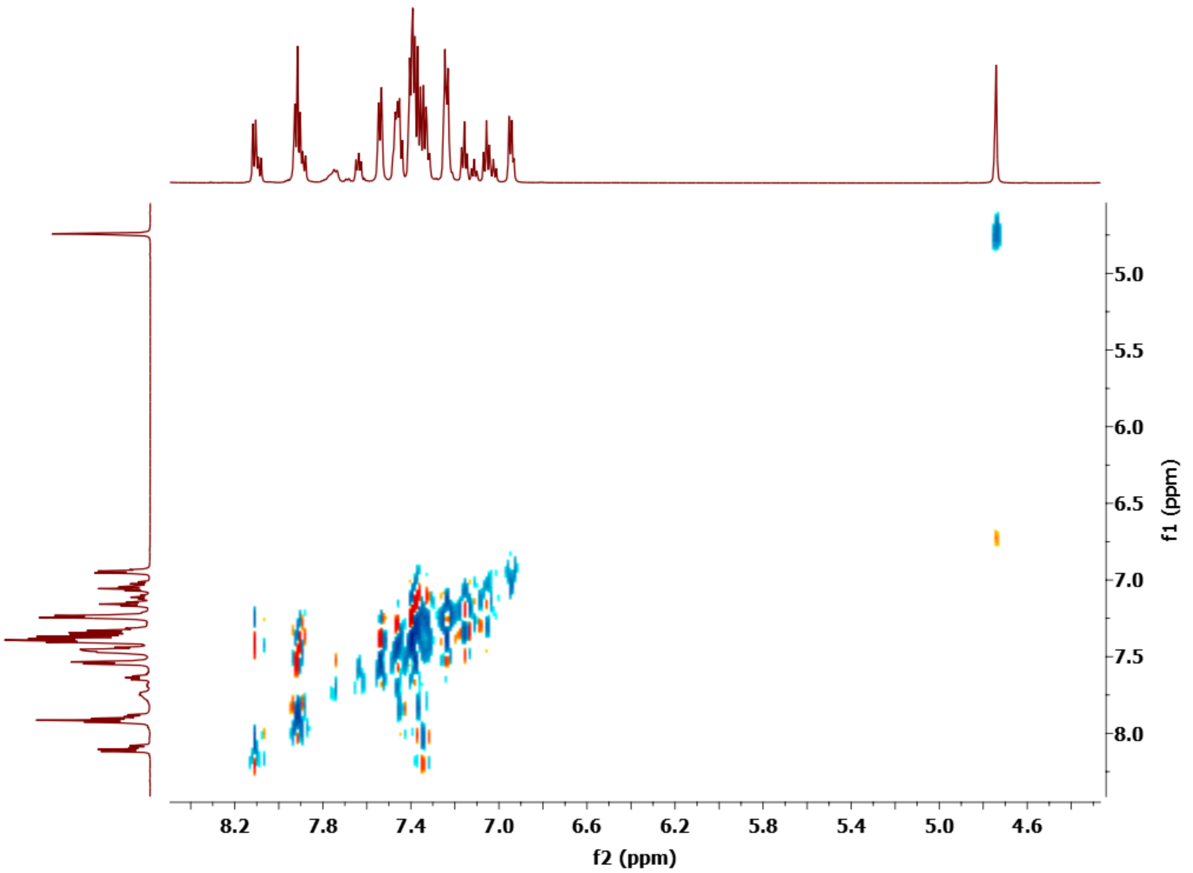

^{1}H-^{13}C-HSQC NMR spectrum of (4'R,5'R)-4'-(4-chlorophenyl)-1''-phenyl-4'H-dispiro[fluorene-9,3'-pyrazole-5',4''-pyrazolidine]-3'',5''-dione (5d) and (4'S,5'R)-4'-(4-chlorophenyl)-1''-phenyl-4'H-dispiro[fluorene-9,3'-pyrazole-5',4''-pyrazolidine]-3'',5''-dione (5'd)

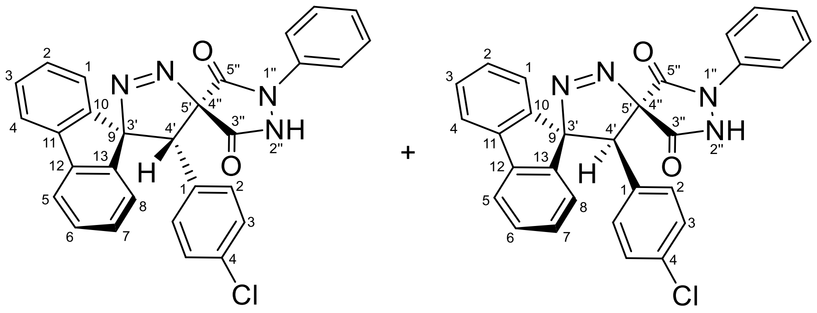

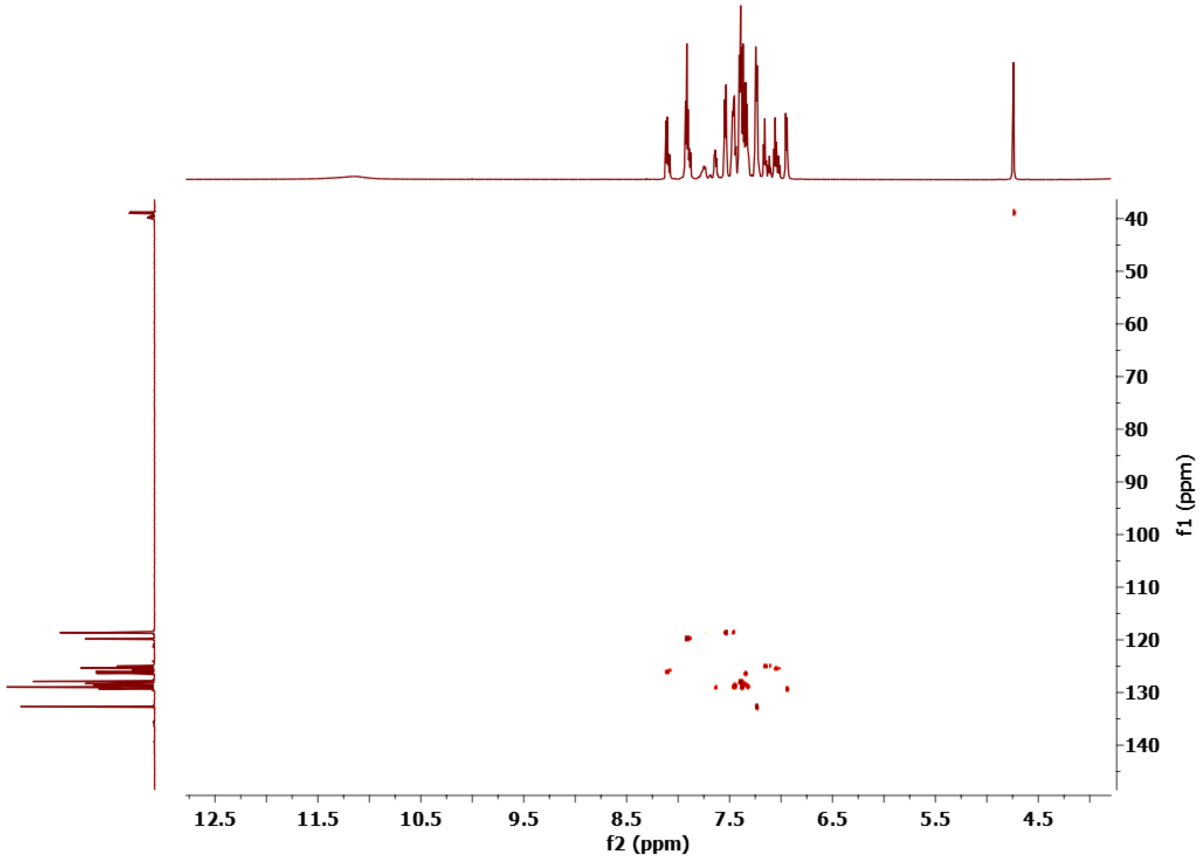

^{1}H-^{13}C-gHMBC NMR spectrum of (4'R,5'R)-4'-(4-chlorophenyl)-1''-phenyl-4'H-dispiro[fluorene-9,3'-pyrazole-5',4''-pyrazolidine]-3'',5''-dione (5d) and (4'S,5'R)-4'-(4-chlorophenyl)-1''-phenyl-4'H-dispiro[fluorene-9,3'-pyrazole-5',4''-pyrazolidine]-3'',5''-dione (5'd)

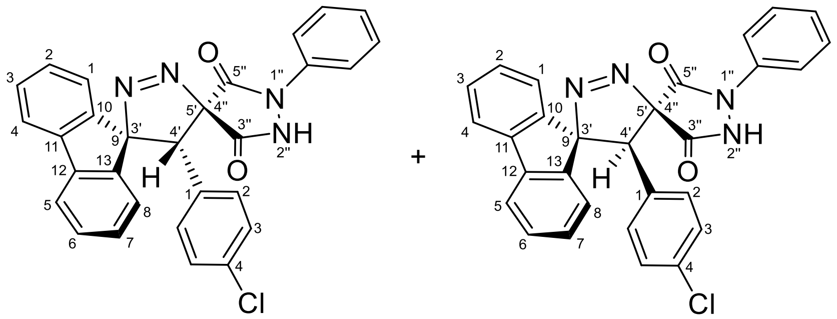

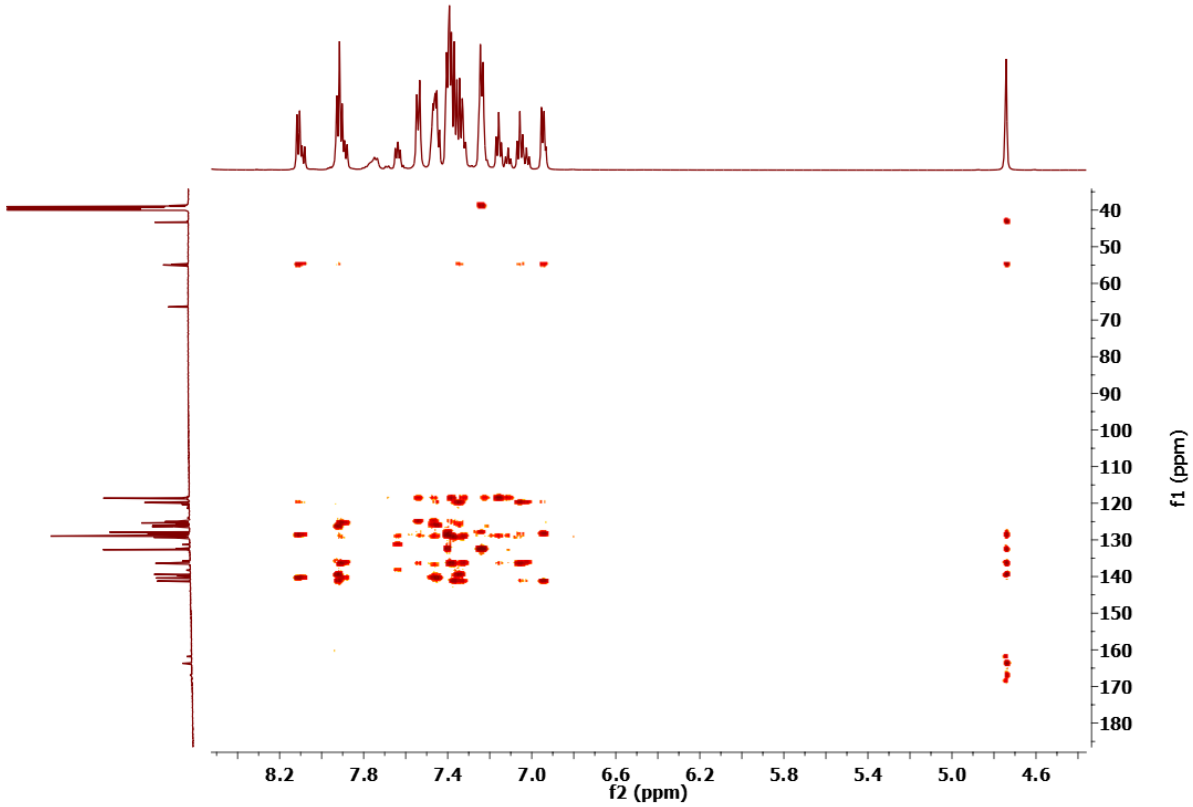

^{1}H-^{1}H-ROESY NMR spectrum of (4'R,5'R)-4'-(4-chlorophenyl)-1''-phenyl-4'H-dispiro[fluorene-9,3'-pyrazole-5',4''-pyrazolidine]-3'',5''-dione (5d) and (4'S,5'R)-4'-(4-chlorophenyl)-1''-phenyl-4'H-dispiro[fluorene-9,3'-pyrazole-5',4''-pyrazolidine]-3'',5''-dione (5'd)

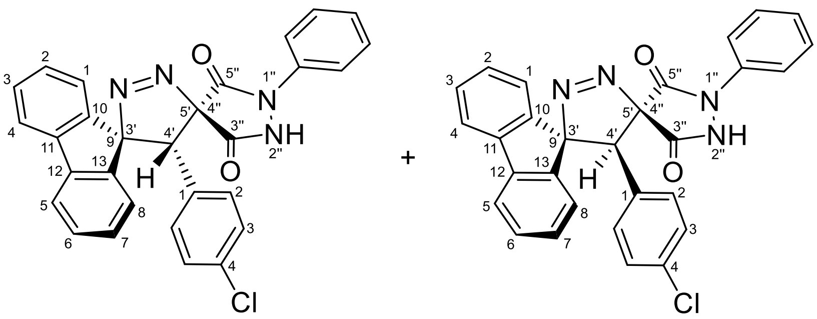

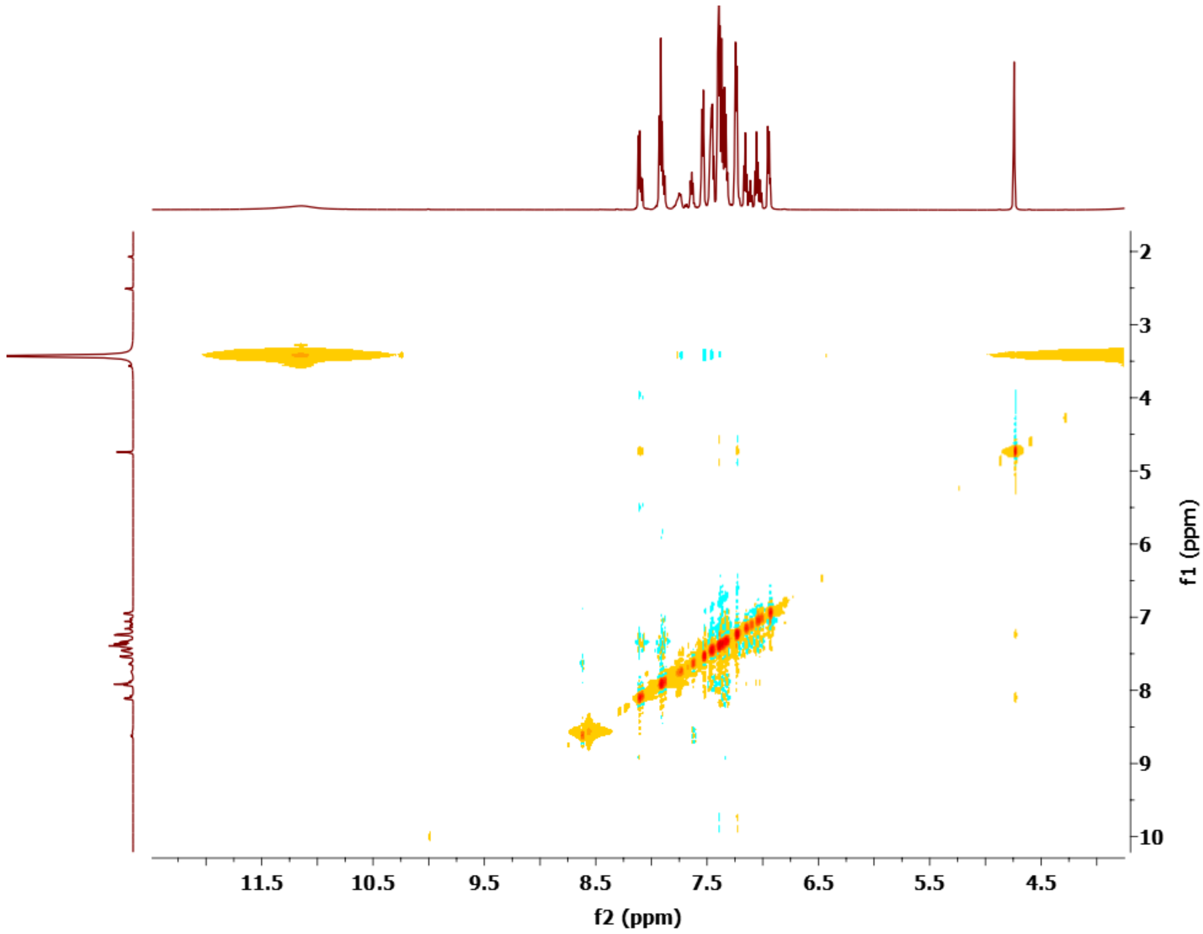

^{1}H NMR spectrum of (4'R,5'R)-4'-(4-fluorophenyl)-1''-phenyl-4'H-dispiro[fluorene-9,3'-pyrazole-5',4''-pyrazolidine]-3'',5''-dione (5e) and (4'S,5'R)-4'-(4-fluorophenyl)-1''-phenyl-4'H-dispiro[fluorene-9,3'-pyrazole-5',4''-pyrazolidine]-3'',5''-dione (5'e)

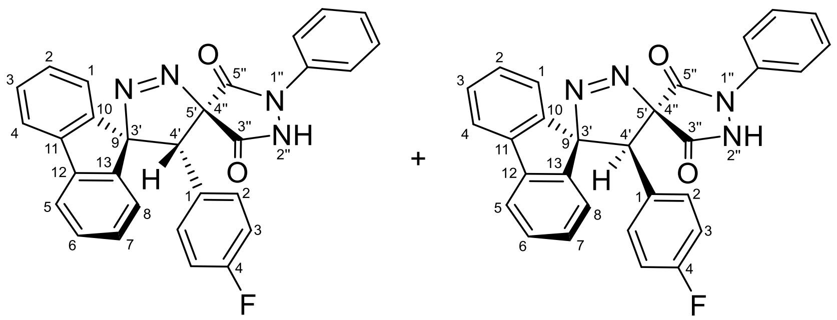

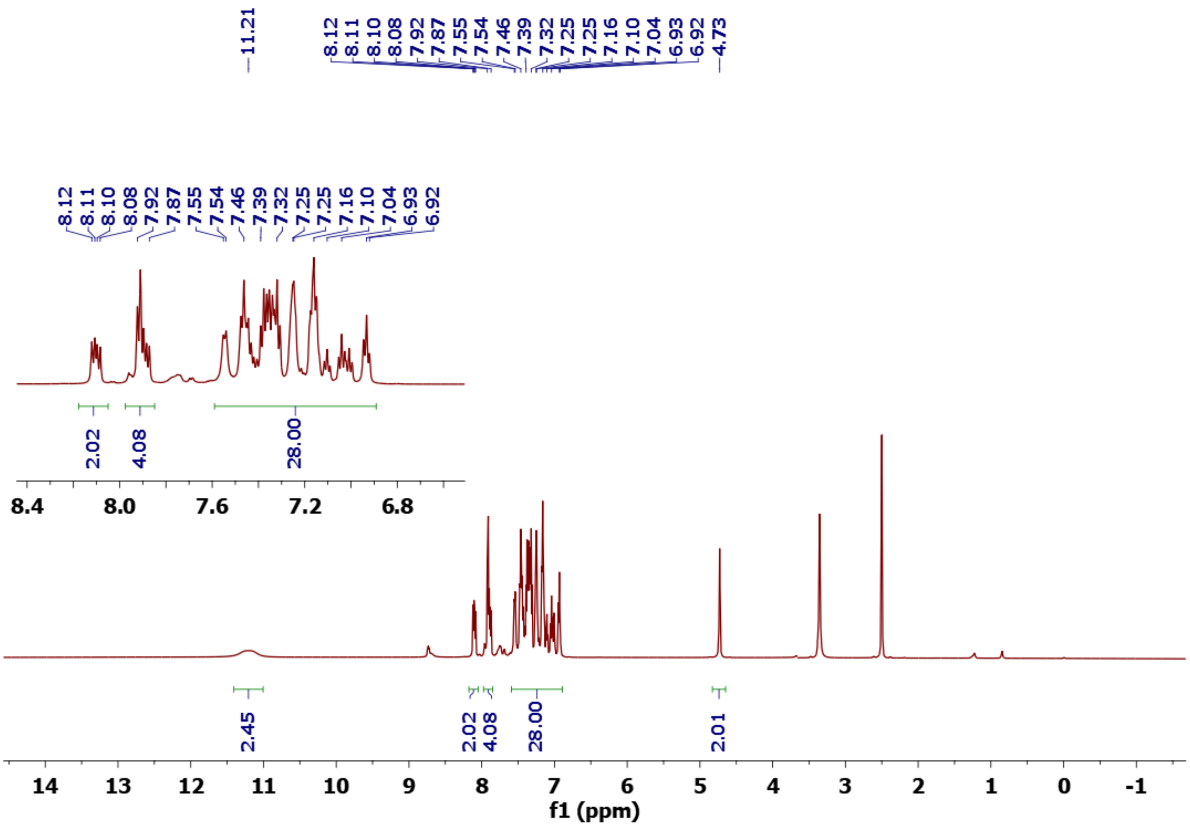

^{13}C NMR spectrum of (4'R,5'R)-4'-(4-fluorophenyl)-1''-phenyl-4'H-dispiro[fluorene-9,3'-pyrazole-5',4''-pyrazolidine]-3'',5''-dione (5e) and (4'S,5'R)-4'-(4-fluorophenyl)-1''-phenyl-4'H-dispiro[fluorene-9,3'-pyrazole-5',4''-pyrazolidine]-3'',5''-dione (5'e)

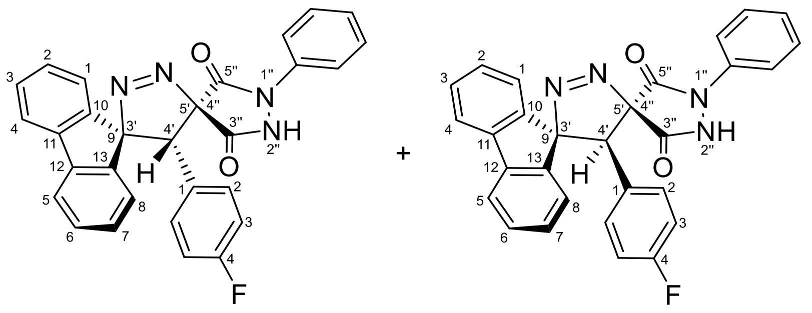

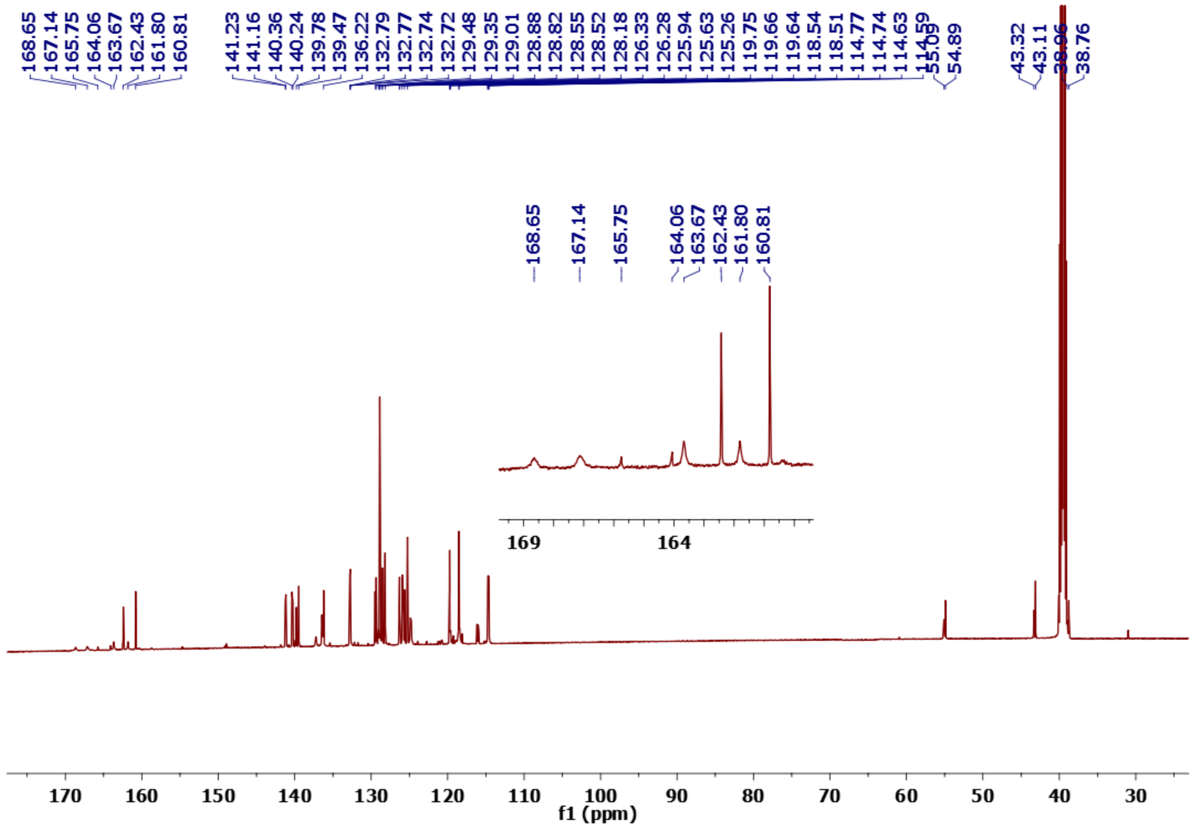

^{13}C DEPT-135 NMR spectrum of (4'R,5'R)-4'-(4-fluorophenyl)-1''-phenyl-4'H-dispiro[fluorene-9,3'-pyrazole-5',4''-pyrazolidine]-3'',5''-dione (5e) and (4'S,5'R)-4'-(4-fluorophenyl)-1''-phenyl-4'H-dispiro[fluorene-9,3'-pyrazole-5',4''-pyrazolidine]-3'',5''-dione (5'e)

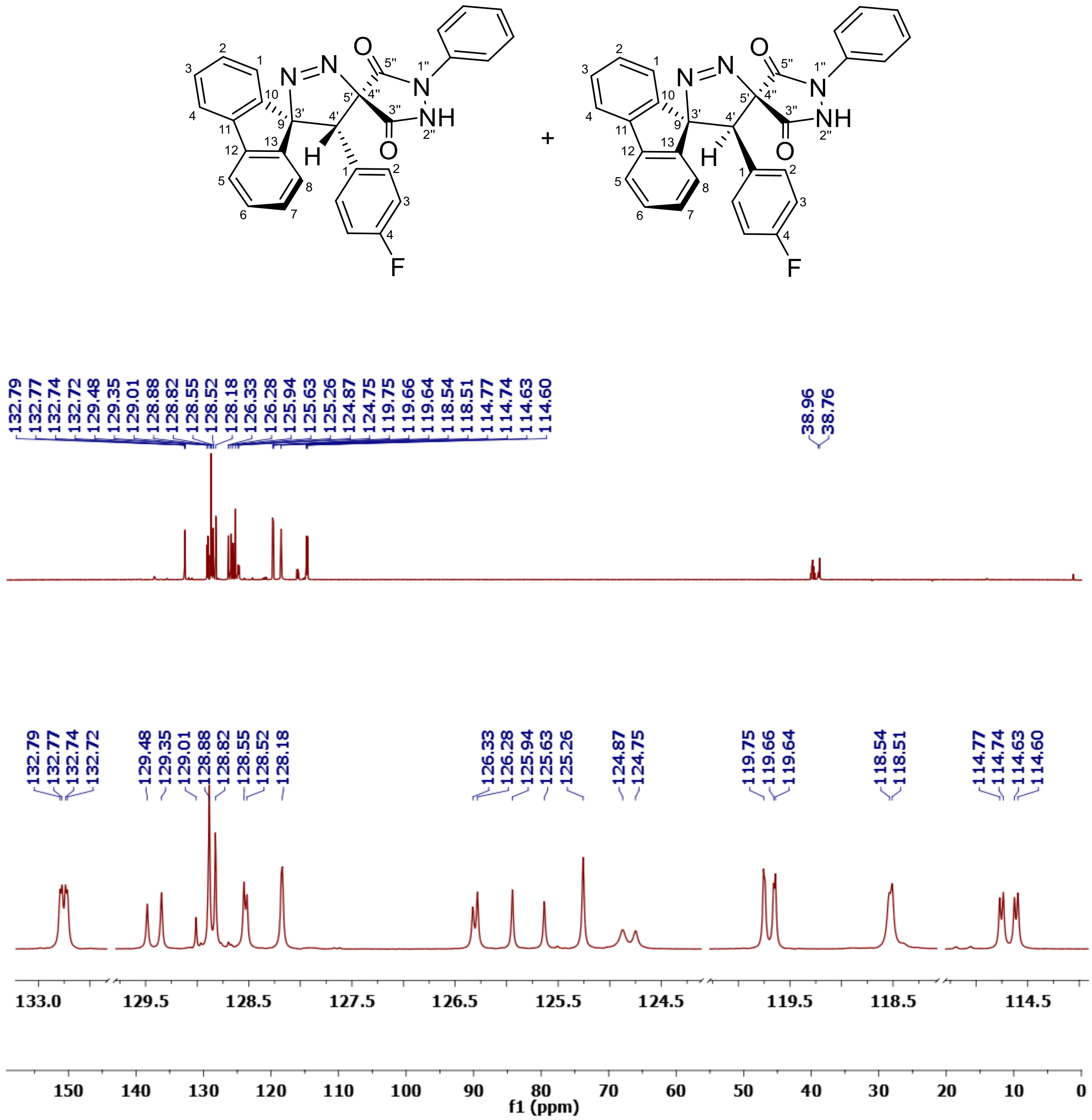

^{1}H-^{1}H gDQCOSY NMR spectrum of (4'R,5'R)-4'-(4-fluorophenyl)-1''-phenyl-4'H-dispiro[fluorene-9,3'-pyrazole-5',4''-pyrazolidine]-3'',5''-dione (5e) and (4'S,5'R)-4'-(4-fluorophenyl)-1''-phenyl-4'H-dispiro[fluorene-9,3'-pyrazole-5',4''-pyrazolidine]-3'',5''-dione (5'e)

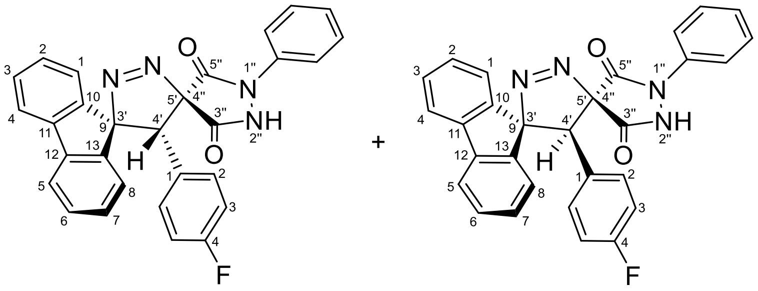

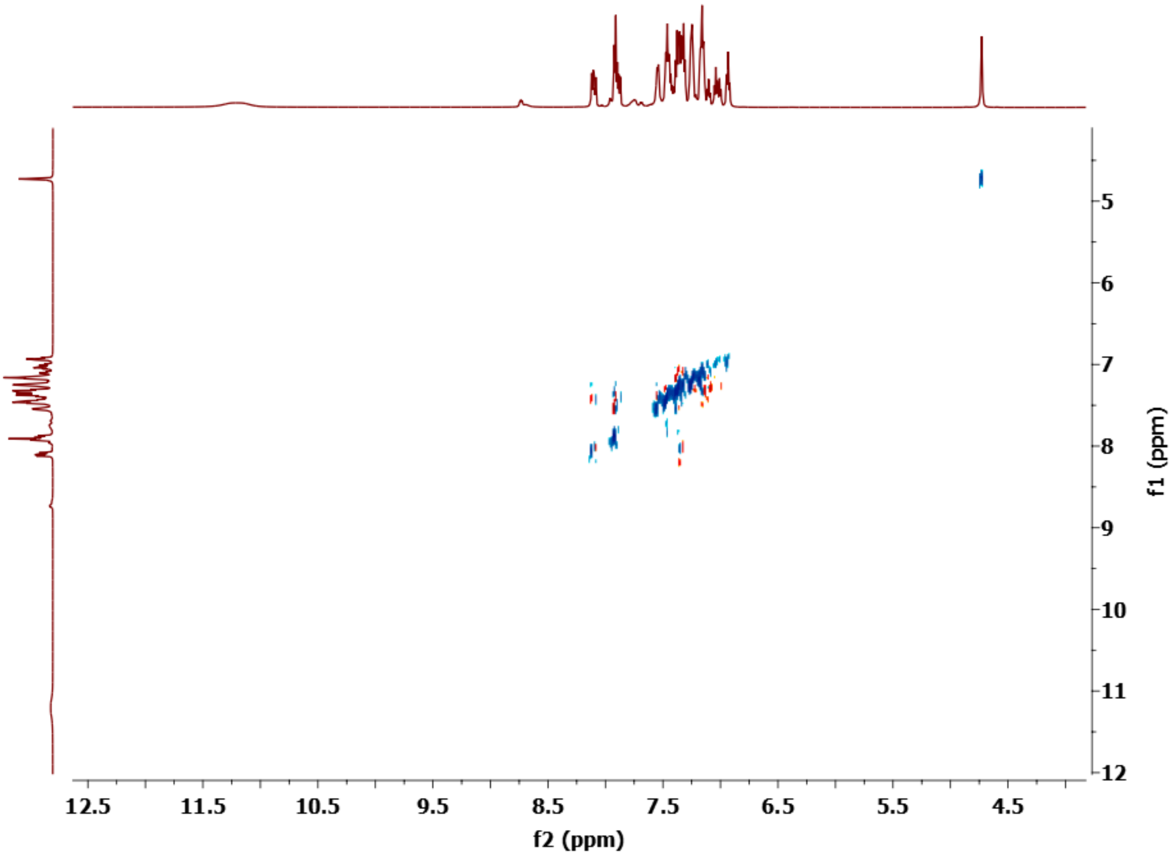

^{1}H-^{13}C-HSQC NMR spectrum of (4'R,5'R)-4'-(4-fluorophenyl)-1''-phenyl-4'H-dispiro[fluorene-9,3'-pyrazole-5',4''-pyrazolidine]-3'',5''-dione (5e) and (4'S,5'R)-4'-(4-fluorophenyl)-1''-phenyl-4'H-dispiro[fluorene-9,3'-pyrazole-5',4''-pyrazolidine]-3'',5''-dione (5'e)

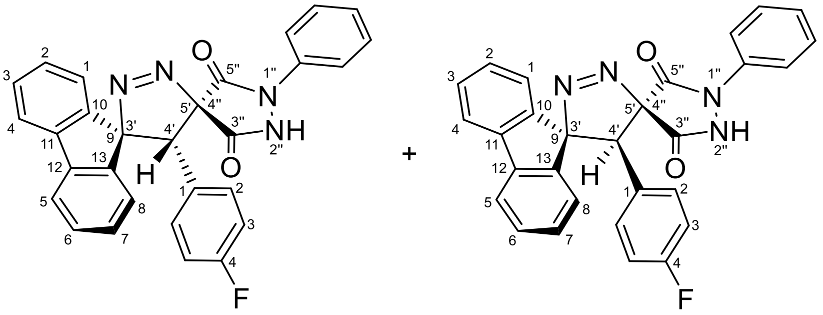

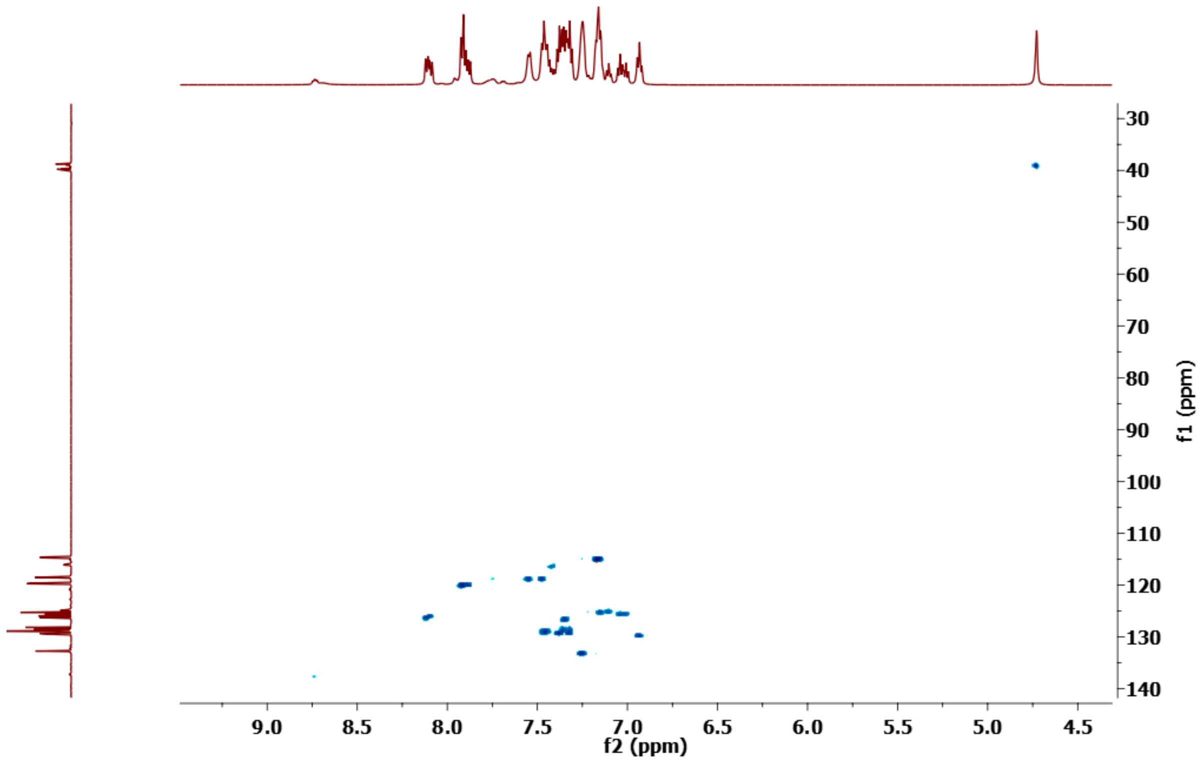

^{1}H-^{13}C-gHMBC NMR spectrum of (4'R,5'R)-4'-(4-fluorophenyl)-1''-phenyl-4'H-dispiro[fluorene-9,3'-pyrazole-5',4''-pyrazolidine]-3'',5''-dione (5e) and (4'S,5'R)-4'-(4-fluorophenyl)-1''-phenyl-4'H-dispiro[fluorene-9,3'-pyrazole-5',4''-pyrazolidine]-3'',5''-dione (5'e)

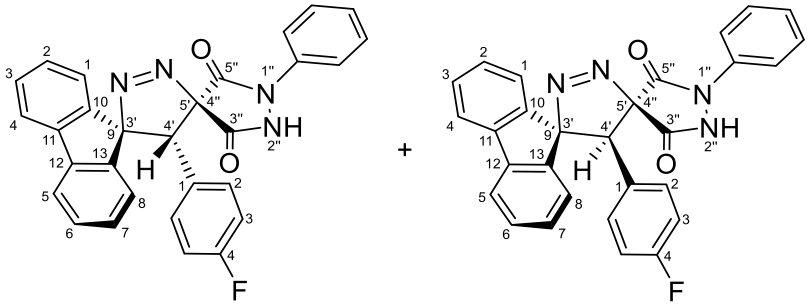

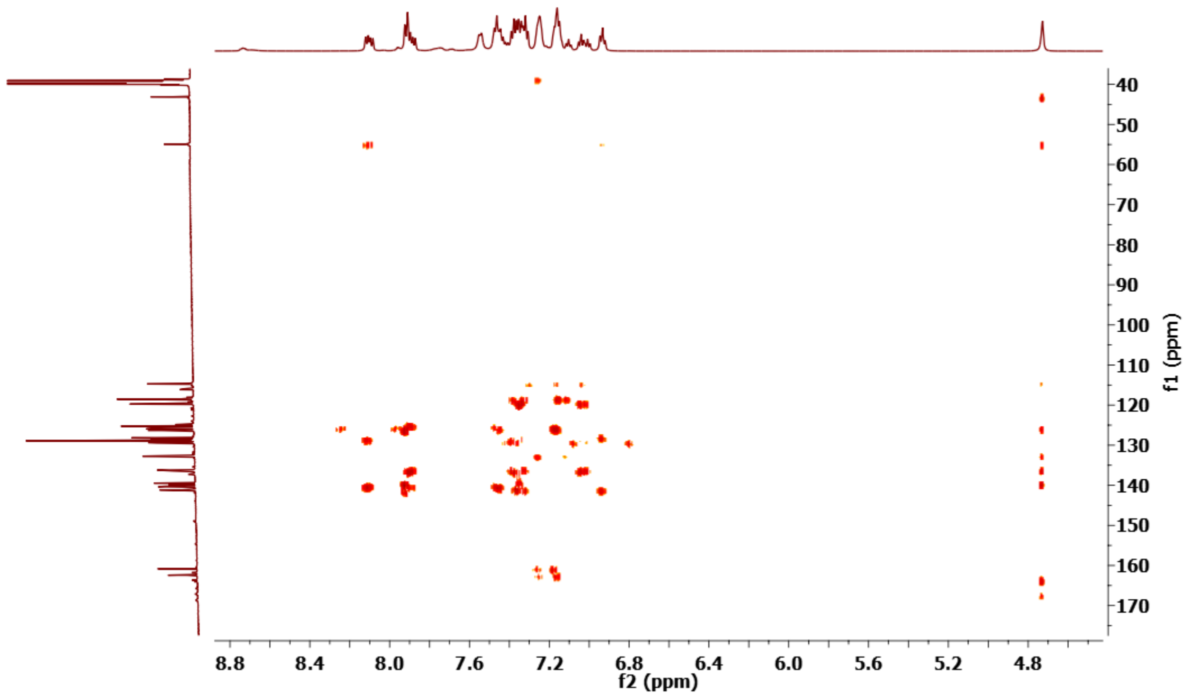

^{1}H NMR spectrum of (4'R,5'R)-4'-(4-cyanophenyl)-1''-phenyl-4'H-dispiro[fluorene-9,3'-pyrazole-5',4''-pyrazolidine]-3'',5''-dione (5f) and (4'S,5'R)-4'-(4-cyanophenyl)-1''-phenyl-4'H-dispiro[fluorene-9,3'-pyrazole-5',4''-pyrazolidine]-3'',5''-dione (5'f)

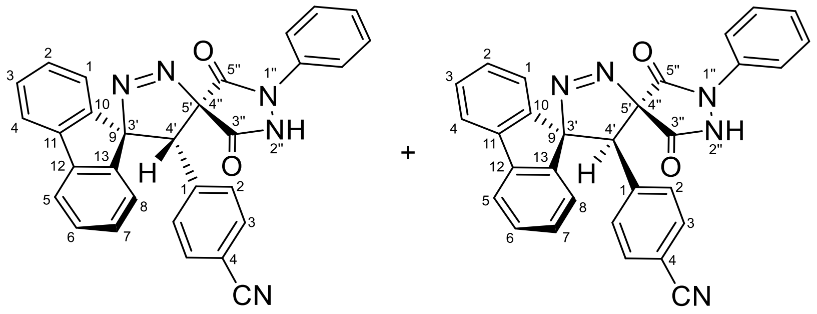

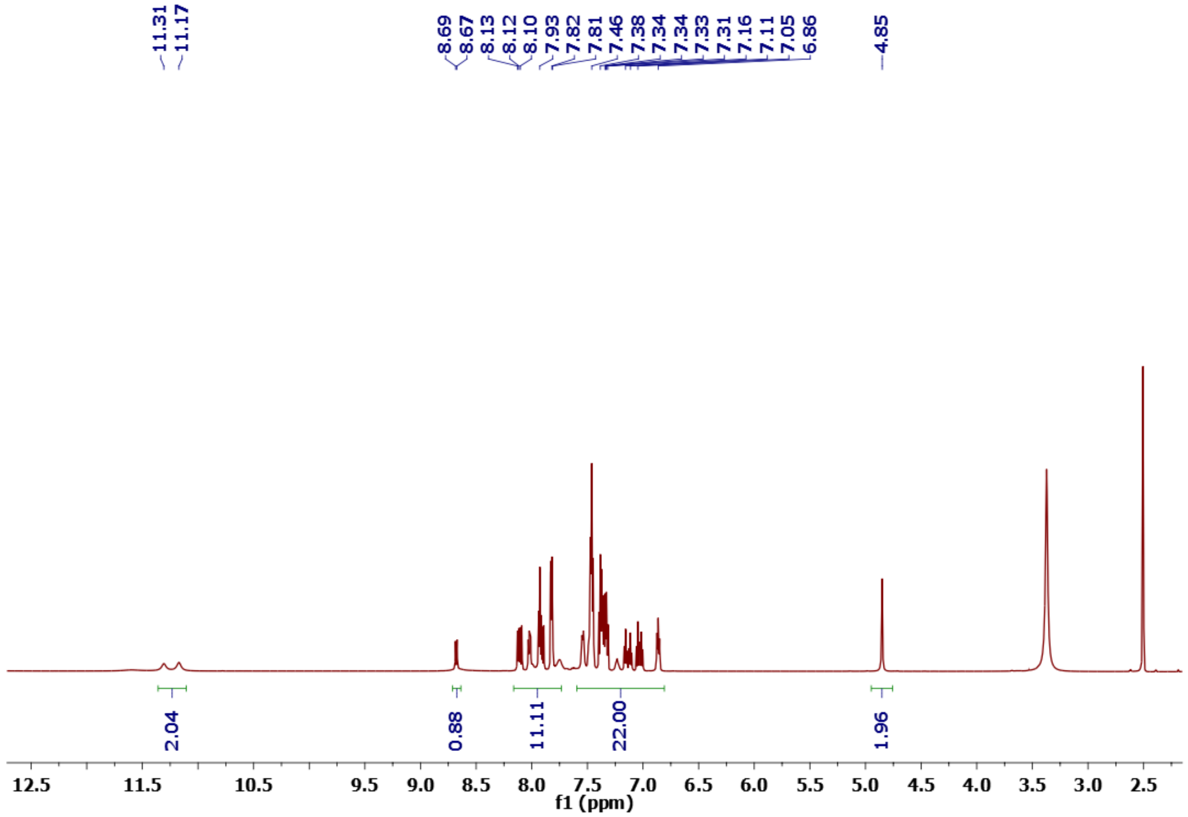

^{13}C NMR spectrum of (4'R,5'R)-4'-(4-cyanophenyl)-1''-phenyl-4'H-dispiro[fluorene-9,3'-pyrazole-5',4''-pyrazolidine]-3'',5''-dione (5f) and (4'S,5'R)-4'-(4-cyanophenyl)-1''-phenyl-4'H-dispiro[fluorene-9,3'-pyrazole-5',4''-pyrazolidine]-3'',5''-dione (5'f)

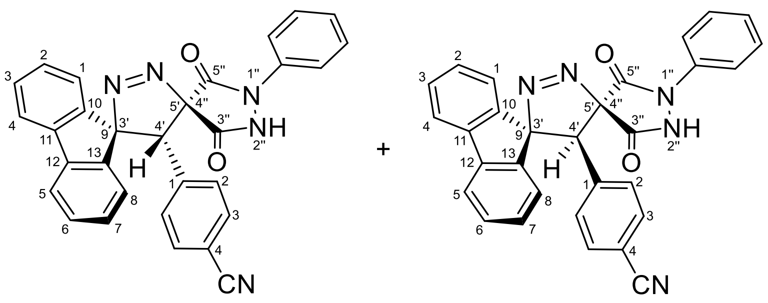

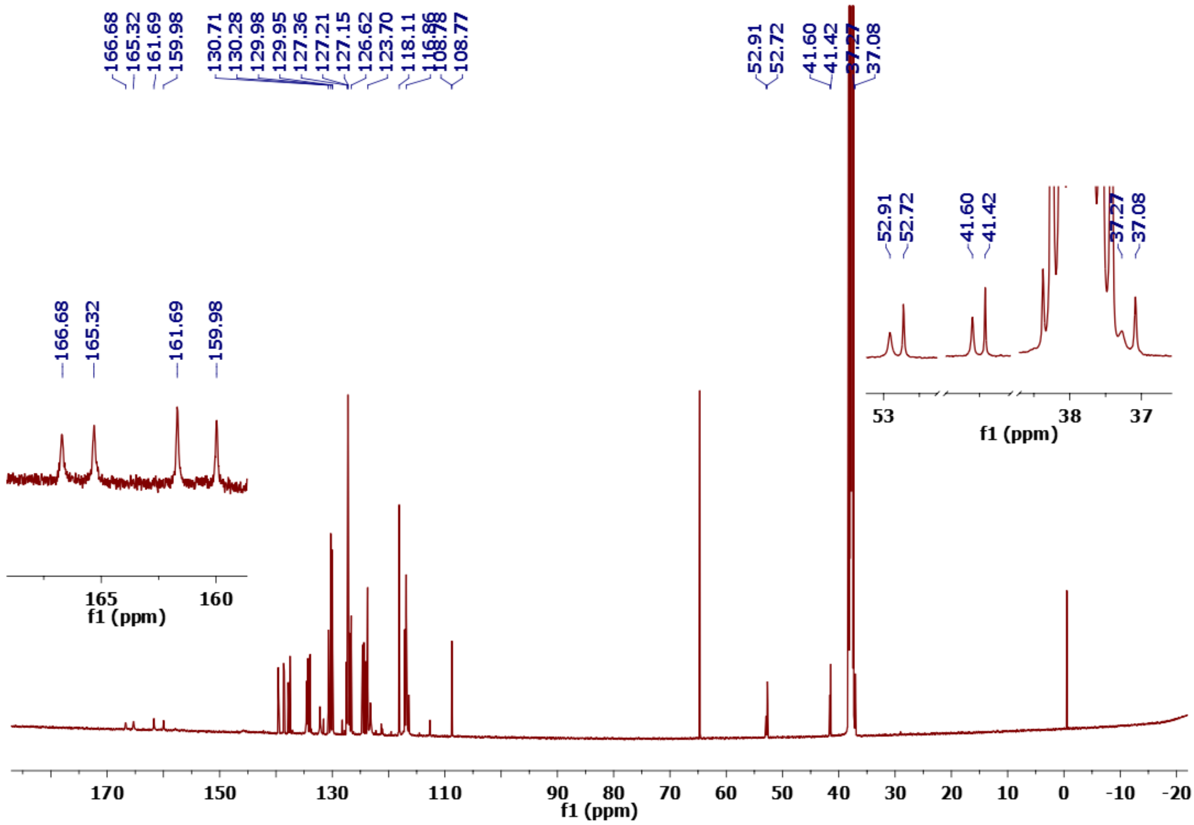

^{13}C DEPT-135 NMR spectrum of (4'R,5'R)-4'-(4-cyanophenyl)-1''-phenyl-4'H-dispiro[fluorene-9,3'-pyrazole-5',4''-pyrazolidine]-3'',5''-dione (5f) and (4'S,5'R)-4'-(4-cyanophenyl)-1''-phenyl-4'H-dispiro[fluorene-9,3'-pyrazole-5',4''-pyrazolidine]-3'',5''-dione (5'f)

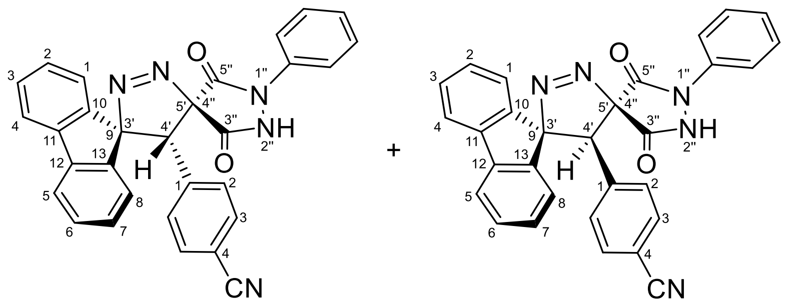

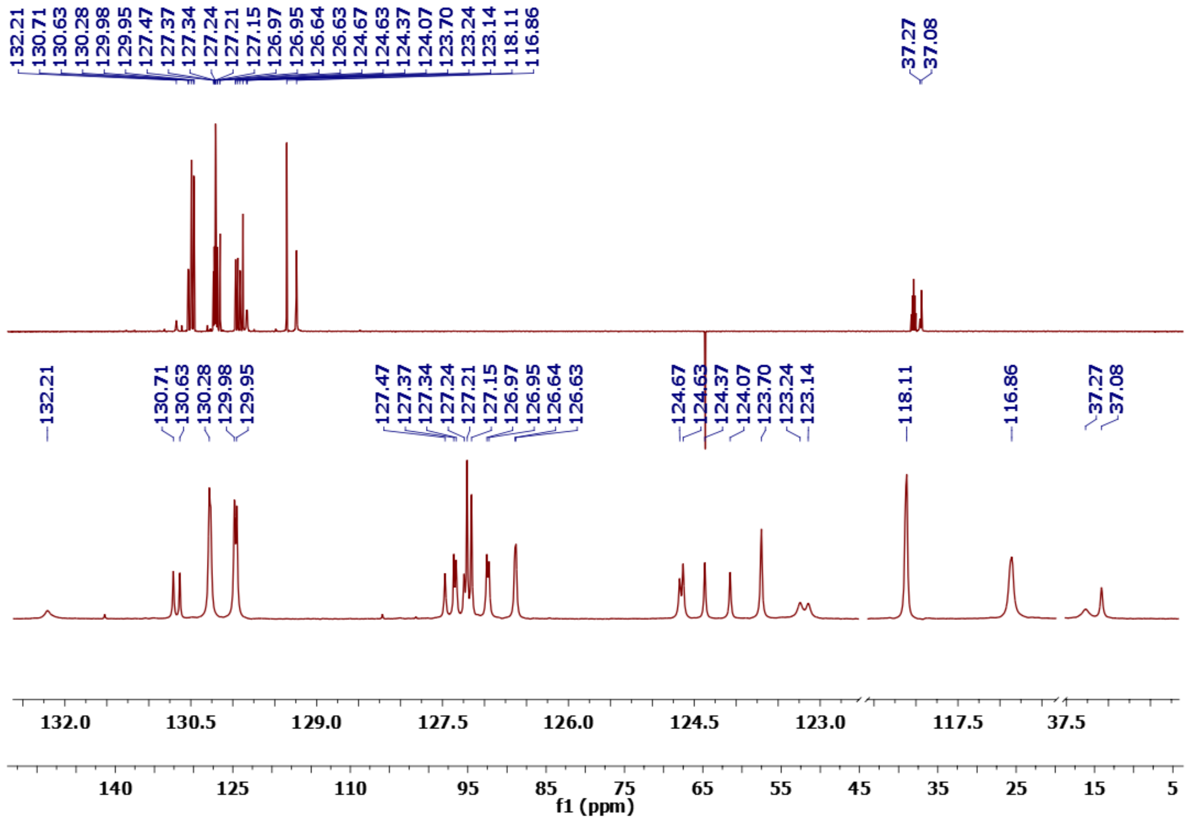

^{1}H-^{1}H gDQCOSY NMR spectrum of (4'R,5'R)-4'-(4-cyanophenyl)-1''-phenyl-4'H-dispiro[fluorene-9,3'-pyrazole-5',4''-pyrazolidine]-3'',5''-dione (5f) and (4'S,5'R)-4'-(4-cyanophenyl)-1''-phenyl-4'H-dispiro[fluorene-9,3'-pyrazole-5',4''-pyrazolidine]-3'',5''-dione (5'f)

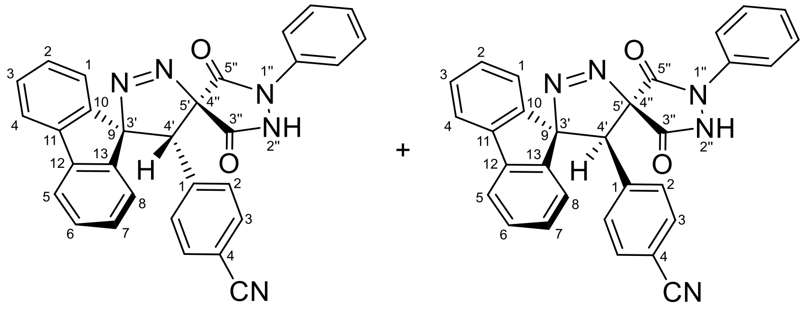

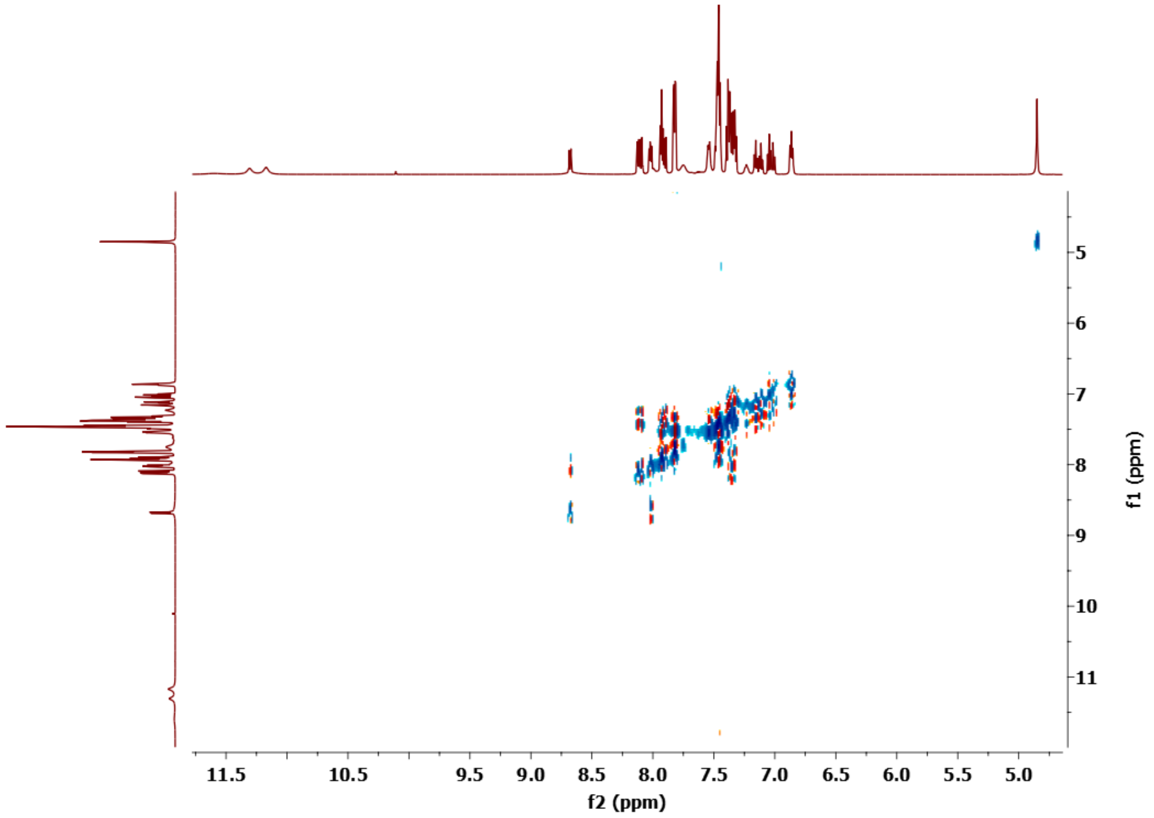

^{1}H-^{13}C-HSQC NMR spectrum of (4'R,5'R)-4'-(4-cyanophenyl)-1''-phenyl-4'H-dispiro[fluorene-9,3'-pyrazole-5',4''-pyrazolidine]-3'',5''-dione (5f) and (4'S,5'R)-4'-(4-cyanophenyl)-1''-phenyl-4'H-dispiro[fluorene-9,3'-pyrazole-5',4''-pyrazolidine]-3'',5''-dione (5'f)

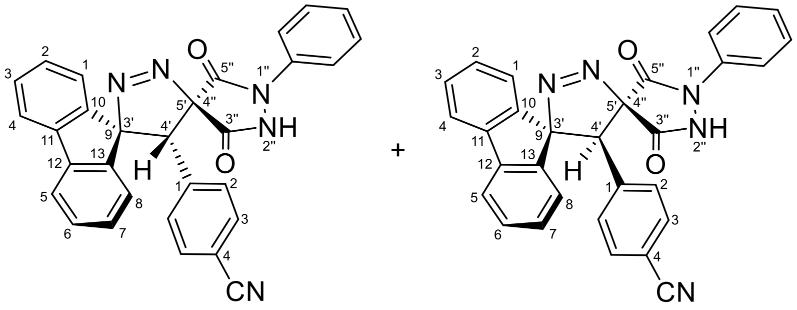

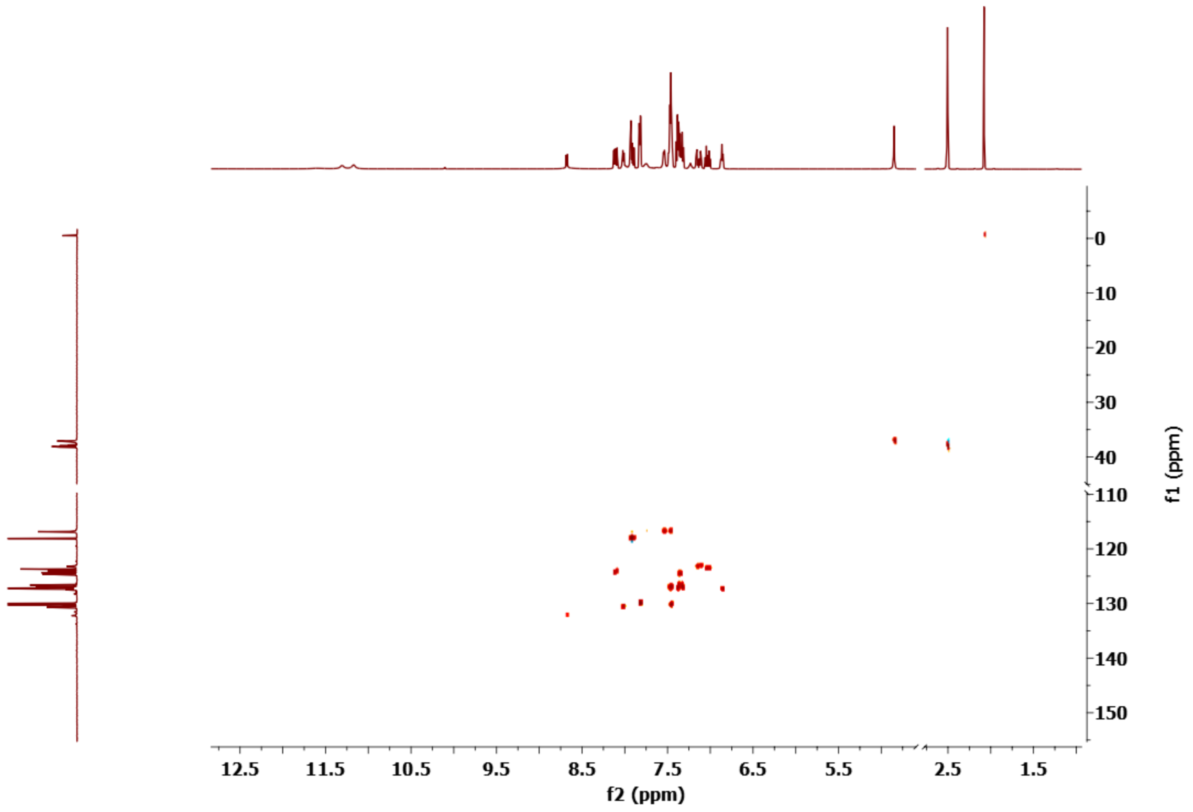

^{1}H-^{13}C-gHMBC NMR spectrum of (4'R,5'R)-4'-(4-cyanophenyl)-1''-phenyl-4'H-dispiro[fluorene-9,3'-pyrazole-5',4''-pyrazolidine]-3'',5''-dione (5f) and (4'S,5'R)-4'-(4-cyanophenyl)-1''-phenyl-4'H-dispiro[fluorene-9,3'-pyrazole-5',4''-pyrazolidine]-3'',5''-dione (5'f)

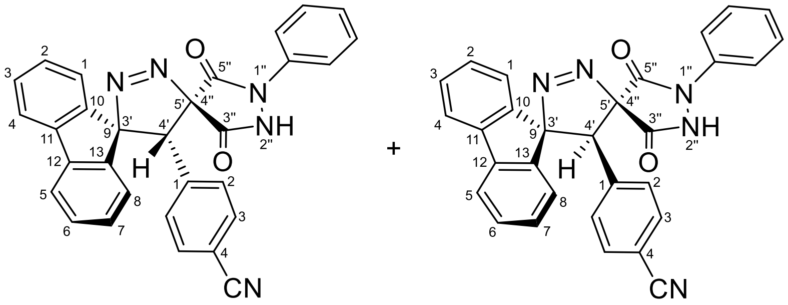

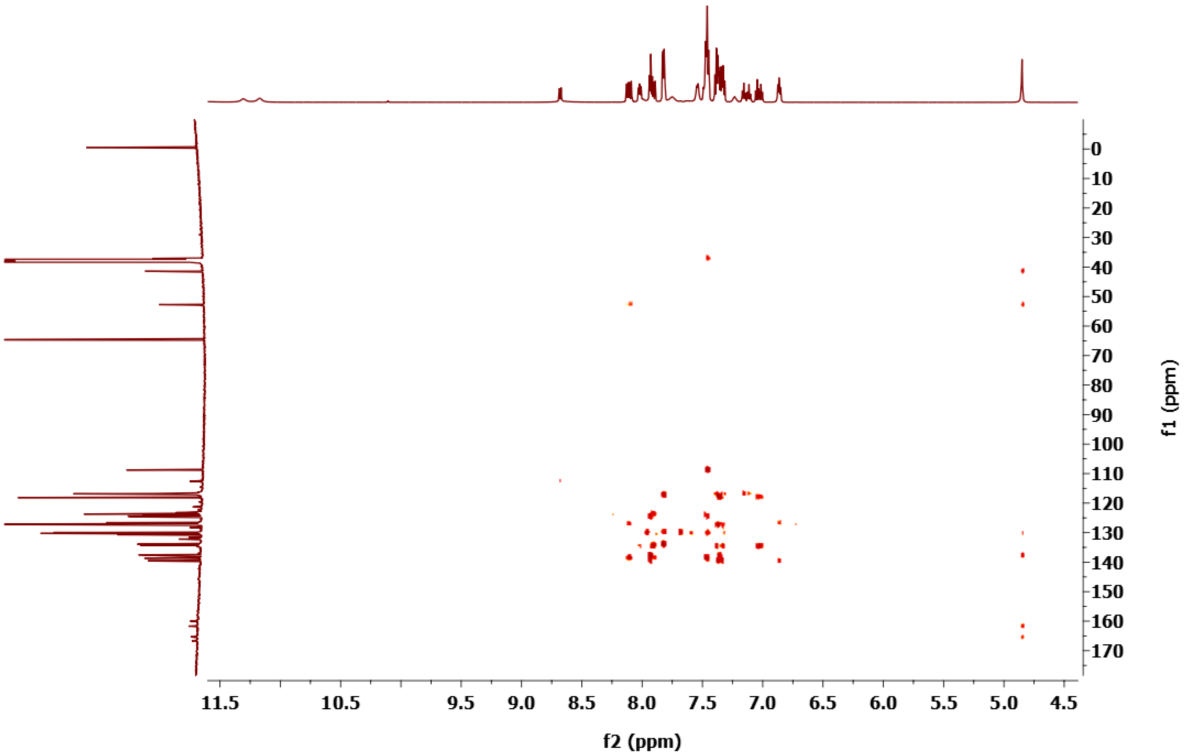

^{1}H NMR spectrum of (4'R,5'R)-4'-(4-nitrophenyl)-1''-phenyl-4'H-dispiro[fluorene-9,3'-pyrazole-5',4''-pyrazolidine]-3'',5''-dione (5g) and (4'S,5'R)-4'-(4-nitrophenyl)-1''-phenyl-4'H-dispiro[fluorene-9,3'-pyrazole-5',4''-pyrazolidine]-3'',5''-dione (5'g)

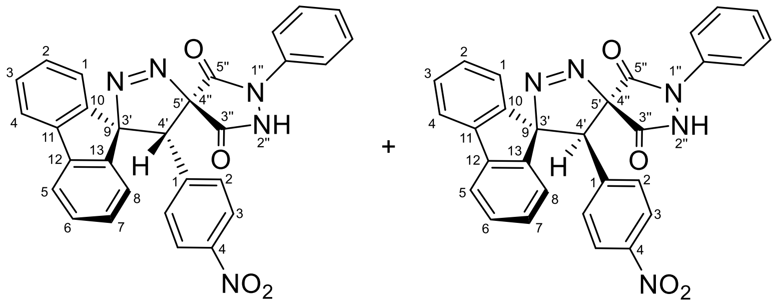

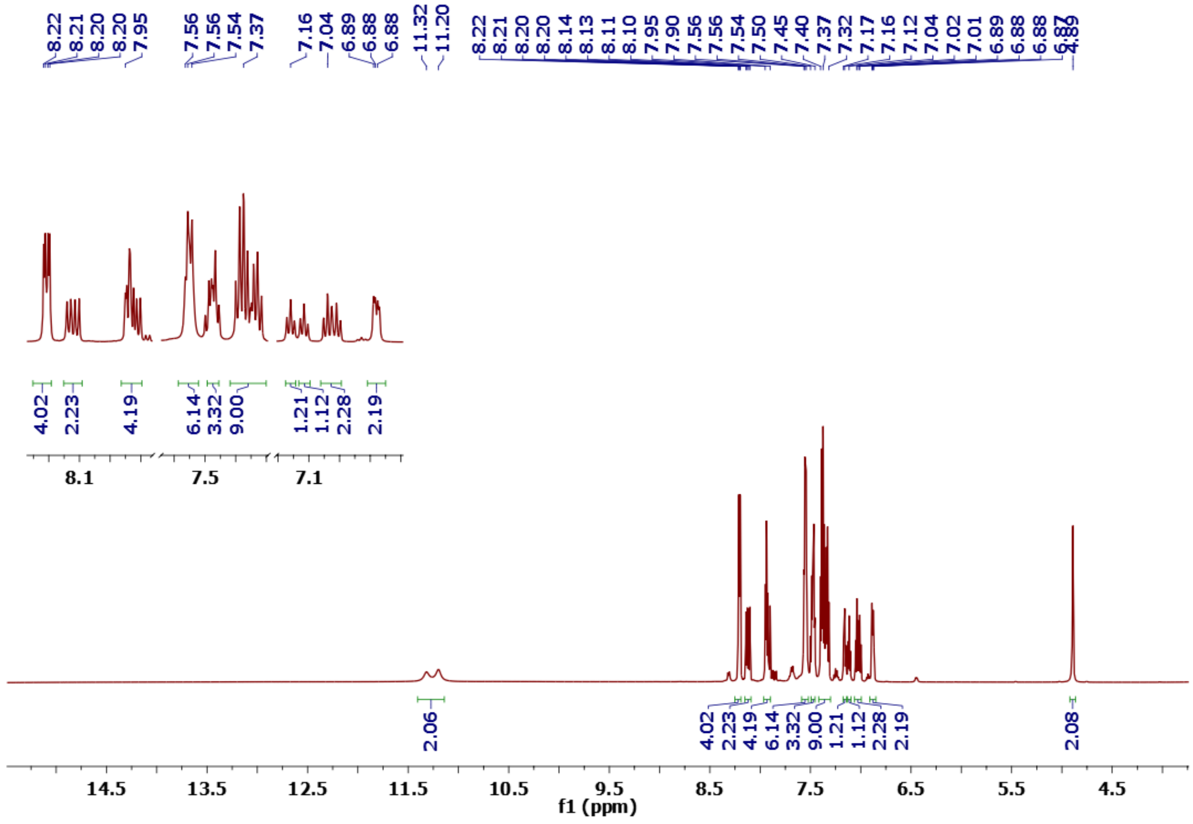

^{13}C NMR spectrum of (4'R,5'R)-4'-(4-nitrophenyl)-1''-phenyl-4'H-dispiro[fluorene-9,3'-pyrazole-5',4''-pyrazolidine]-3'',5''-dione (5g) and (4'S,5'R)-4'-(4-nitrophenyl)-1''-phenyl-4'H-dispiro[fluorene-9,3'-pyrazole-5',4''-pyrazolidine]-3'',5''-dione (5'g)

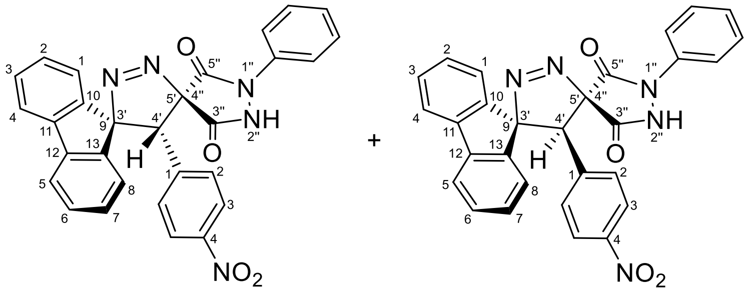

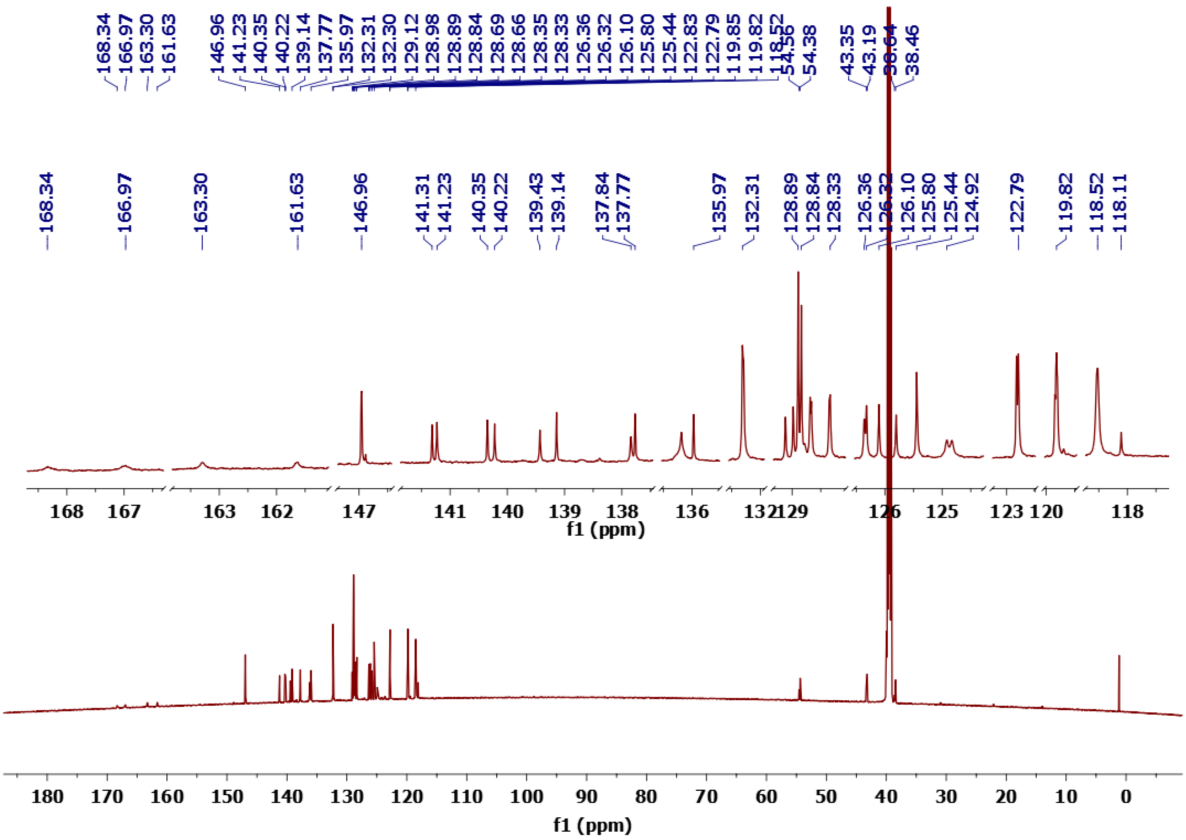

^{13}C DEPT-135 NMR spectrum of (4'R,5'R)-4'-(4-nitrophenyl)-1''-phenyl-4'H-dispiro[fluorene-9,3'-pyrazole-5',4''-pyrazolidine]-3'',5''-dione (5g) and (4'S,5'R)-4'-(4-nitrophenyl)-1''-phenyl-4'H-dispiro[fluorene-9,3'-pyrazole-5',4''-pyrazolidine]-3'',5''-dione (5'g)

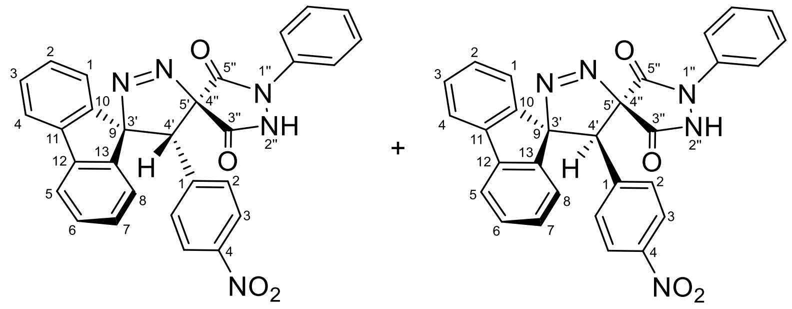

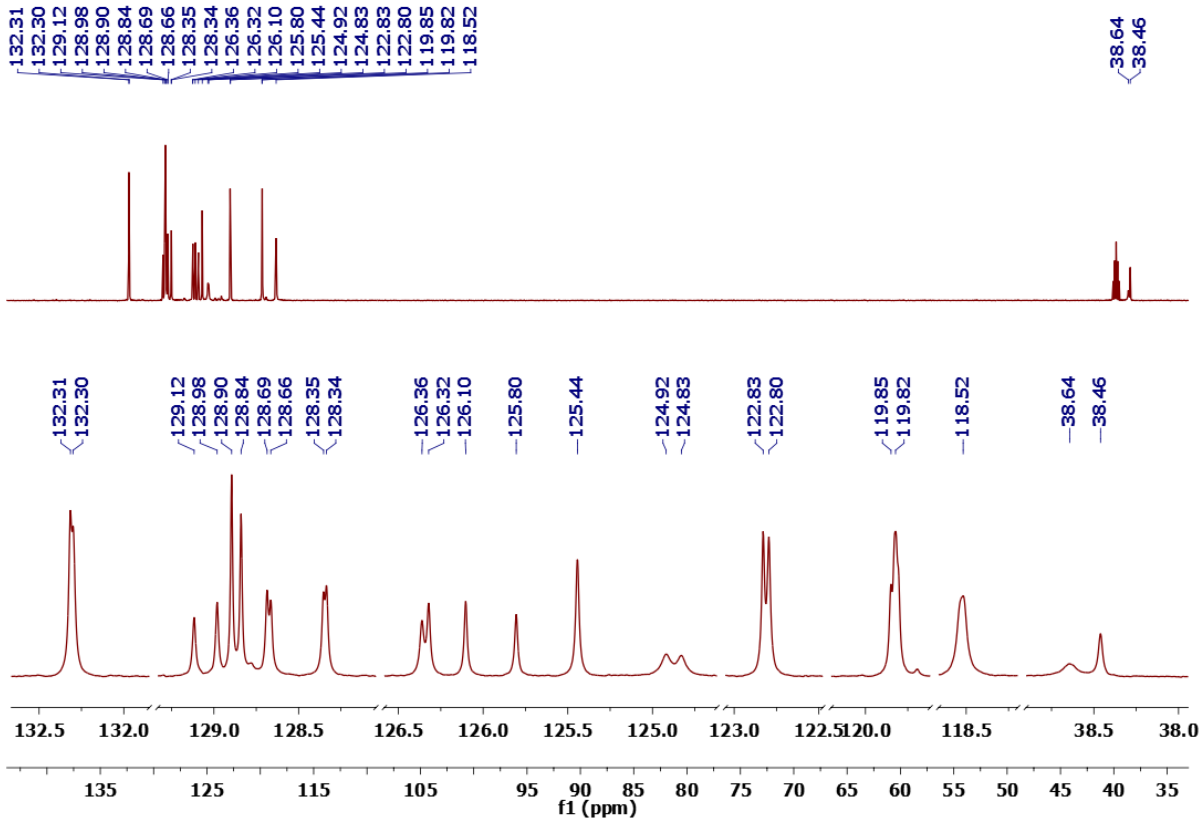

^{1}H-^{1}H gDQCOSY NMR spectrum of (4'R,5'R)-4'-(4-nitrophenyl)-1''-phenyl-4'H-dispiro[fluorene-9,3'-pyrazole-5',4''-pyrazolidine]-3'',5''-dione (5g) and (4'S,5'R)-4'-(4-nitrophenyl)-1''-phenyl-4'H-dispiro[fluorene-9,3'-pyrazole-5',4''-pyrazolidine]-3'',5''-dione (5'g)

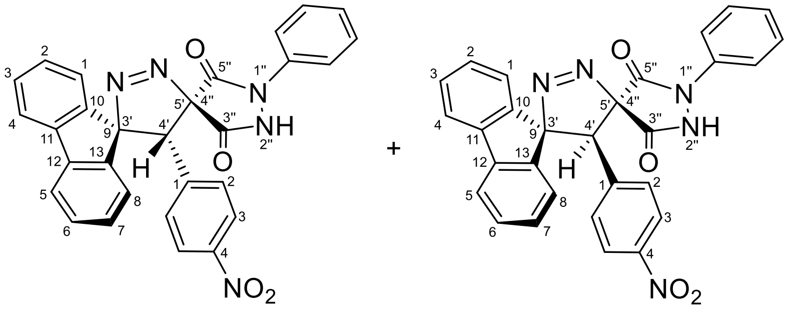

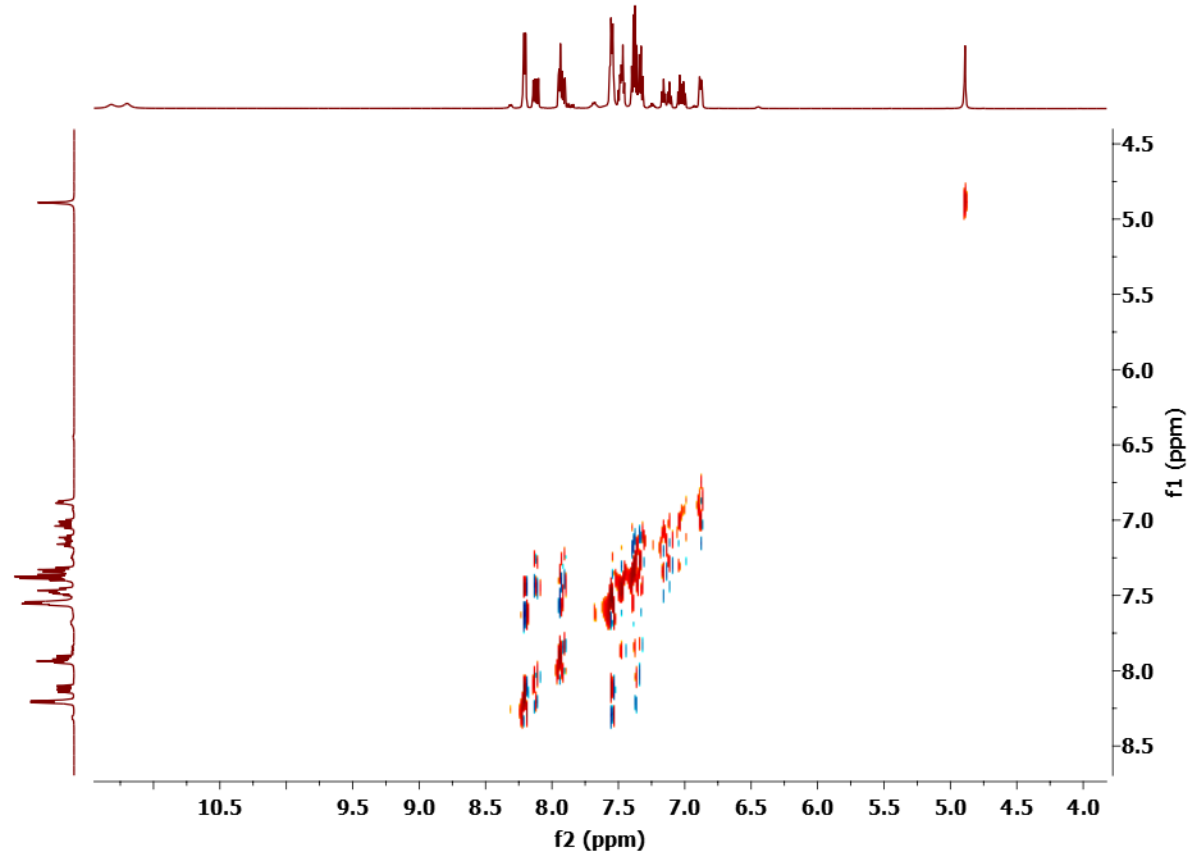

^{1}H-^{13}C-HSQC NMR spectrum of (4'R,5'R)-4'-(4-nitrophenyl)-1''-phenyl-4'H-dispiro[fluorene-9,3'-pyrazole-5',4''-pyrazolidine]-3'',5''-dione (5g) and (4'S,5'R)-4'-(4-nitrophenyl)-1''-phenyl-4'H-dispiro[fluorene-9,3'-pyrazole-5',4''-pyrazolidine]-3'',5''-dione (5'g)

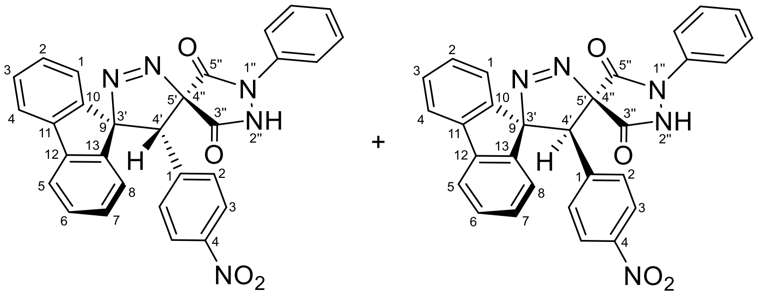

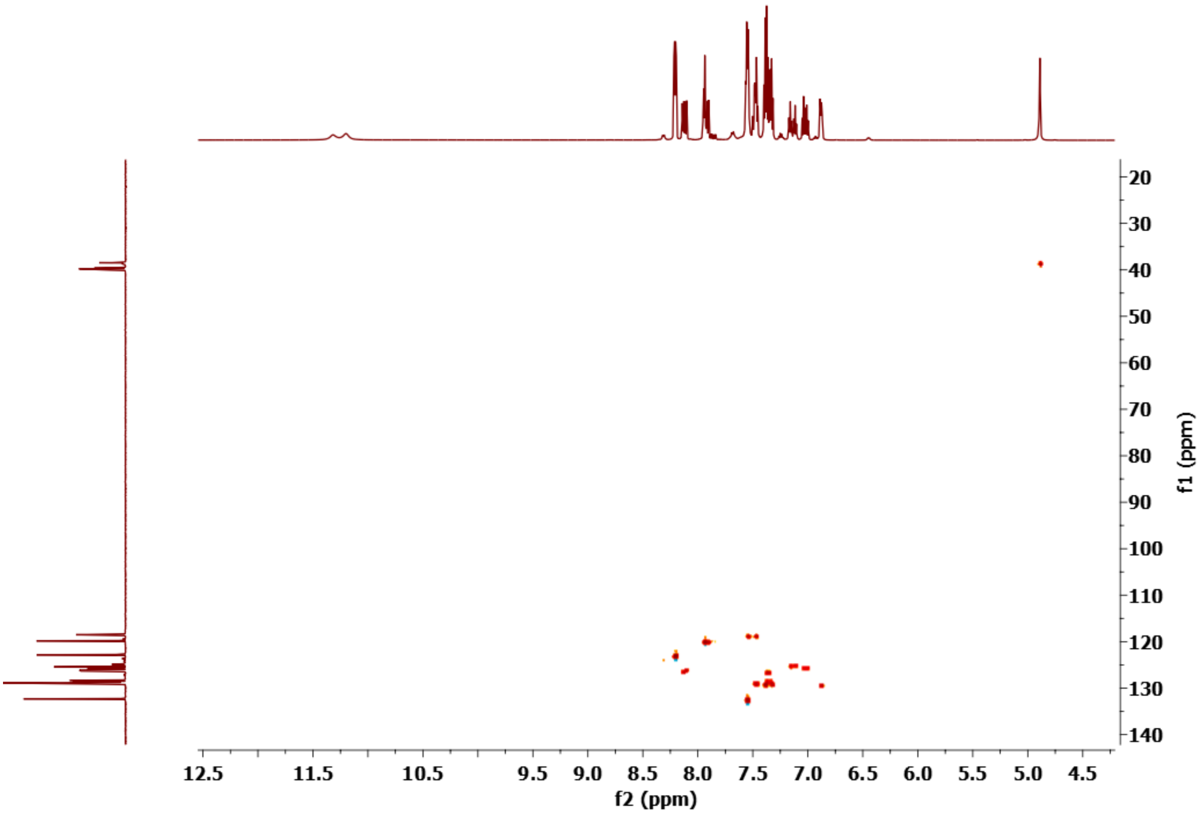

^{1}H-^{13}C-gHMBC NMR spectrum of (4'R,5'R)-4'-(4-nitrophenyl)-1''-phenyl-4'H-dispiro[fluorene-9,3'-pyrazole-5',4''-pyrazolidine]-3'',5''-dione (5g) and (4'S,5'R)-4'-(4-nitrophenyl)-1''-phenyl-4'H-dispiro[fluorene-9,3'-pyrazole-5',4''-pyrazolidine]-3'',5''-dione (5'g)

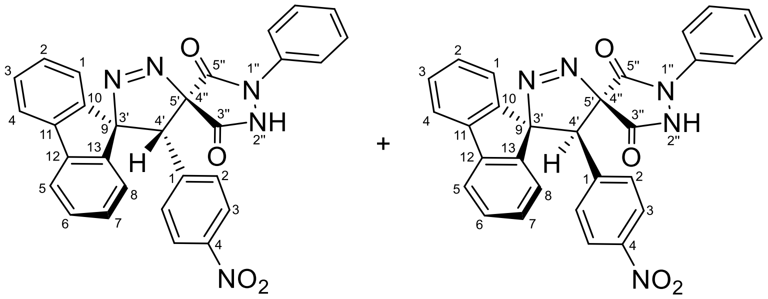

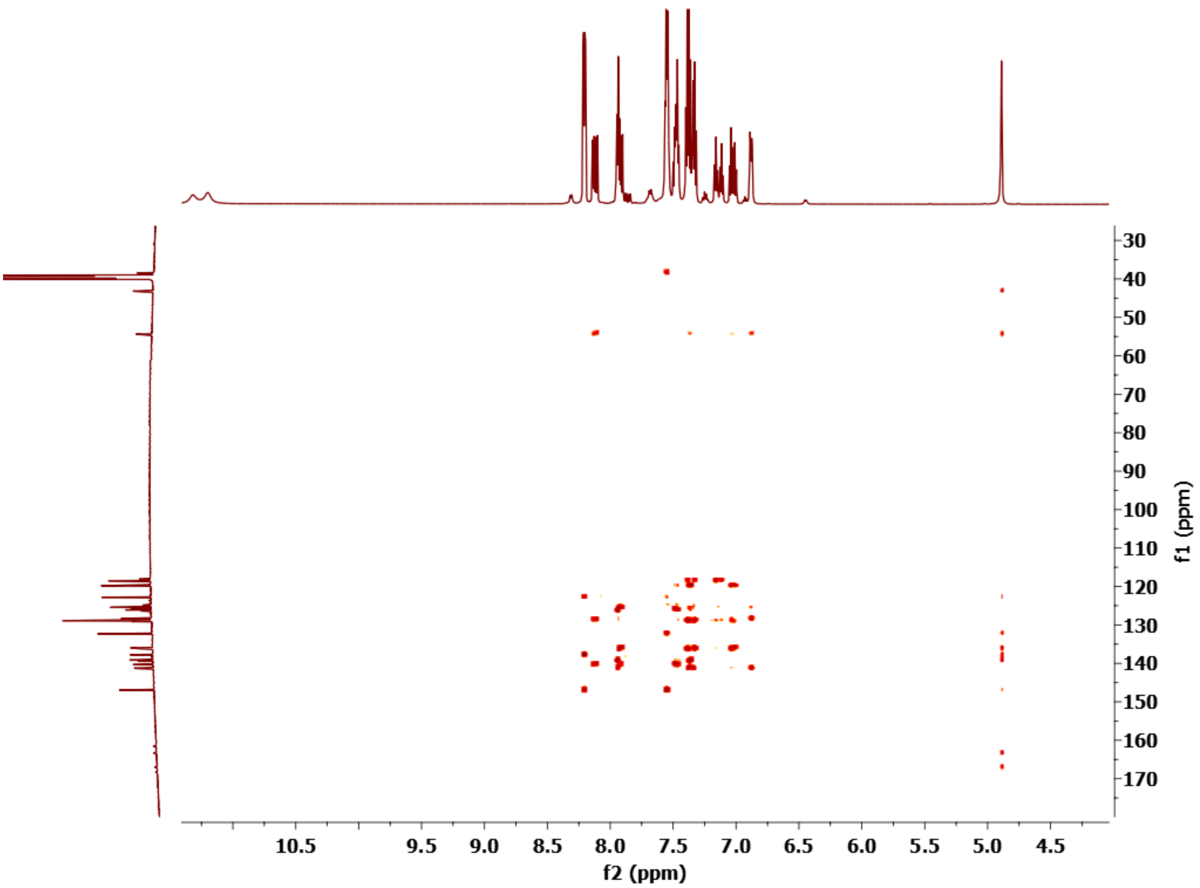

Supplement: RA-016-D6RA01559J-s001 [file RA-016-D6RA01559J-s001.pdf]
